# The Human Takes It All: Humanlike Synthesized Voices Are Perceived as Less Eerie and More Likable. Evidence From a Subjective Ratings Study

**DOI:** 10.3389/fnbot.2020.593732

**Published:** 2020-12-16

**Authors:** Katharina Kühne, Martin H. Fischer, Yuefang Zhou

**Affiliations:** Division of Cognitive Sciences, University of Potsdam, Potsdam, Germany

**Keywords:** human-robot interaction, paralinguistic features, synthesized voice, text-to-speech, uncanny valley

## Abstract

**Background:** The increasing involvement of social robots in human lives raises the question as to how humans perceive social robots. Little is known about human perception of synthesized voices.

**Aim:** To investigate which synthesized voice parameters predict the speaker's eeriness and voice likability; to determine if individual listener characteristics (e.g., personality, attitude toward robots, age) influence synthesized voice evaluations; and to explore which paralinguistic features subjectively distinguish humans from robots/artificial agents.

**Methods:** 95 adults (62 females) listened to randomly presented audio-clips of three categories: synthesized (*Watson*, IBM), humanoid (robot *Sophia*, Hanson Robotics), and human voices (five clips/category). Voices were rated on intelligibility, prosody, trustworthiness, confidence, enthusiasm, pleasantness, human-likeness, likability, and naturalness. Speakers were rated on appeal, credibility, human-likeness, and eeriness. Participants' personality traits, attitudes to robots, and demographics were obtained.

**Results:** The human voice and human speaker characteristics received reliably higher scores on all dimensions except for eeriness. Synthesized voice ratings were positively related to participants' agreeableness and neuroticism. Females rated synthesized voices more positively on most dimensions. Surprisingly, interest in social robots and attitudes toward robots played almost no role in voice evaluation. Contrary to the expectations of an uncanny valley, when the ratings of human-likeness for both the voice and the speaker characteristics were higher, they seemed less eerie to the participants. Moreover, when the speaker's voice was more humanlike, it was more liked by the participants. This latter point was only applicable to one of the synthesized voices. Finally, pleasantness and trustworthiness of the synthesized voice predicted the likability of the speaker's voice. Qualitative content analysis identified intonation, sound, emotion, and imageability/embodiment as diagnostic features.

**Discussion:** Humans clearly prefer human voices, but manipulating diagnostic speech features might increase acceptance of synthesized voices and thereby support human-robot interaction. There is limited evidence that human-likeness of a voice is negatively linked to the perceived eeriness of the speaker.

## Introduction

The last 20 years have seen a steady growth in the development of social robots. Compared to traditional industrial robots that are often designed to perform dull, dangerous, and dirty jobs by replacing human beings, social robots are made to interact closely with humans in collaborative contexts (Broadbent, 2017). More recently, there has been an immense increase in involvement of social robots in different spheres of human life, such as social, entertainment, and health care settings, as well as in commerce and education (Kory-Westlund et al., 2016; Breazeal, 2017; Belpaeme et al., 2018). In these applications roboticists are trying to design social robots to mimic human appearance, behavior, and emotional expression.

When robots increasingly look and behave like humans, this raises the questions of whether and how human likeness promotes effective human-robot interaction. These fundamental questions must be considered for each of the sensory modalities of social interaction. Scientists have made great progress in understanding the relationship between our visual perception of robots' human likeness and our resulting attitudes toward them. However, other sensory modalities have been disproportionally neglected. Considering the importance of verbal communication in establishing cooperation and trust, we will focus here on the perception of artificial voices after briefly reviewing some basic issues that became apparent in the scientific study of visual appearances of humanoid robots.

### Visual Perception of Humanoids

A humanoid robot is a robot with a body shape similar to a human, usually with a head, torso, arms, and legs (Broadbent, 2017). This morphological similarity not only ensures the humanoid's functionality as collaborator in human environments but also constitutes the interface for our communicative habits, such as eye contact or a handshake (Breazeal, 2002). Generally, human liking of humanoid robots increases when the robots appear more humanlike, thus expressing a basic principle of social psychology: the similarity attraction effect (e.g., Berscheid and Reis, 1998; Nass and Lee, 2001; Bernier and Scassellati, 2010). However, this trend holds only up to a certain degree of high similarity, when the robot begins to look imperfectly human. At this point a spontaneous feeling of eeriness and strangeness emerges in most observers, and this changing attitude can be visualized by plotting the degree of liking against the degree of similarity to a human. The result of such a plot is the famous “uncanny valley” function that reflects a gradual increase in liking up to a sudden, valley-like drop before we express maximal liking for real human interaction partners (Mori, 1970; Strait et al., 2015).

The “uncanny valley” phenomenon has been widely observed in experiments with visual stimuli (MacDorman, 2006; Bartneck et al., 2007; Seymour et al., 2017; Kätsyri et al., 2019; Pütten et al., 2019). Among other factors, visual liking of humanoids depends on the degree of anthropomorphism in their appearance (Kim et al., 2019), their attitude (Złotowski et al., 2018), expressed emotion (Tschöpe et al., 2017), nonverbal behavior (Thepsoonthorn et al., 2018), motion (Castro-González et al., 2016), their gender (Kraus et al., 2018), and also on participants' personality traits (MacDorman and Entezari, 2015).

While a whole range of different attributes were found to be associated with the “uncanny valley” in visual tasks, comparatively little is known about the impact of synthesized voice qualities on likability and eeriness (Kuratate et al., 2009; Mitchell et al., 2011; Romportl, 2014; Chang et al., 2018). For instance, a very recent study evaluated the likability and human-likeness of a corpus of 13 German male voices, produced via five different synthesis approaches, and found that contrary to the visual “uncanny valley,” likability increases monotonically with human-likeness of the voice (Baird et al., 2018). A study by Romportl (2014) showed that about three quarters of participants preferred a more natural voice over an artificial one. However, the authors added that affinity to artificial agents might be an important intervening factor in voice perception. The present study took the first step to understanding the relationship between human-likeness and eeriness in artificial voices.

### Auditory Perception of Humanoids

In oral communication, both the semantic content of speech and its intelligibility play an important role (for review, see Massaro, 1987; Massaro and Simpson, 2014). Focusing on artificially generated speech, researchers have investigated a variety of approaches to evaluating artificial voices in terms of their usability and quality (Kayte et al., 2016; Hinterleitner, 2017; Uhrig et al., 2017). For example, text-to-speech (TTS) synthesis describes the conversion of text to synthetically generated speech (Kayte et al., 2016). TTS is used in user interfaces for the blind, for translation software but also in humanoid robots. Schmidt-Nielsen (1995) distinguished between a synthesized voice's intelligibility (how understandable it is) and its acceptability (perceived voice quality).

Hinterleitner (2017) recently specified five dimensions of quality for TTS systems: naturalness of voice, prosodic quality, fluency and intelligibility, absence of disturbances, and calmness. While most synthesized voices have reached a high level of intelligibility, it is the perceived quality that still requires clarification (Polkosky and Lewis, 2003; Wagner et al., 2019). Moreover, the quality of the voice was shown to be important in establishing a positive human-robot relationship even when the content of the utterances was unintelligible (McGinn and Torre, 2019).

In addition to enabling information exchange, auditory language provides important information about the speaker, their attitude and state of mind, as well as their relationship to the interlocutor (Liscombe et al., 2003; Bombelli et al., 2013). The impact of such paralinguistic voice features on the perception of a speaker's personality, emotional state, or the quality of the interaction remain a matter of debate. These subjective evaluations are, however, crucial for social situations such as healthcare or education, where humanoid robots may soon become prevalent (Schuller et al., 2013).

Key dimensions of successful social interactions are credibility and trustworthiness, so these dimensions have been evaluated in studies of human-robot interactions. Torre et al. (2018) demonstrated that participants formed their impressions about a robot's trustworthiness upon hearing its voice for the first time and combining this impression with the behavior of the robot. In an investment game experiment, the authors found that a robot with a synthetic voice was trusted more when it behaved in a generous way while a robot with a human voice was trusted more when it behaved in a mean way. These findings showed that both context and the robot's voice contribute to the robot's being perceived as trustworthy or not.

Testing a virtual human tutor against synthesized and human voices, Craig and Schroeder (2017) found that an improved human-like or a human voice was rated as more credible, and that the learning ratings with these types of voice were better than those with a “classic” robotic synthesized voice. Further, the pitch of the artificial voice was also found to be crucial for perceived quality of communication (Niculescu et al., 2013). In particular, the manipulation of a robot's voice pitch affected users' perceived interaction with the robot: The higher-pitched robot was rated higher on overall appearance, voice appeal, behavior, personality, overall interaction enjoyment and quality, and also entertainment.

As for human-likeness, the emotional tone of a synthesized voice has a huge impact not only on the overall perceived quality of the voice but also on the interaction with the artificial agent (Hirai et al., 2015; Yamamoto et al., 2018). Imbuing human-like emotion onto a synthesized voice remains a challenging task because the unique feature combinations entering the voice synthesis are still to be identified (Yamamoto et al., 2018).

In creating synthesized voices, the idea of “personification” has played a major role: voice interfaces are claimed to be more appealing when they resemble a human being by having human traits such as accent, emotion, and even gender (Harris, 2004). As Vallee (2017, p. 91) put it, “a voice is the most intimate part of subjectivity.” Harmony, a customizable personal companion agent, was equipped with a Scottish accent to make her sound more endearing (Coursey et al., 2019). In the same vein, Sims et al. (2009) demonstrated that robots that sounded more human were perceived as more capable. Similarly, robots with human-like voices were also rated as more effective and were remembered better, which may be decisive factors in educational or medical contexts (Rodero, 2017).

### The Present Study

In the present study, two state-of-the-art synthesized voices were contrasted with a human voice in order to establish an informative comparison between currently available synthetic voice options: CereVoice® text-to-speech voice, which is already utilized in the well-known humanoid robot *Sophia*, designed by Hanson Robotics; and a Text-to-Speech voice produced by IBM's *Watson*, which is implemented in various translation products and virtual assistants. These artificial voices were compared to an average English native speaker's female voice.

*Sophia* is Hanson Robotics' most advanced human-like robot and a platform for advanced robotics research. This robot was the first to receive citizenship and to speak in numerous settings such as on the *Tonight Show* and on *Good Morning Britain*, as well as at multiple international conferences. *Sophia* is able to recognize faces and voices, to keep eye contact, and to communicate with people by using CereVoice® text-to-speech engine and Alphabet Inc's voice recognition system. The software of the robot is self-learning and can be constantly improved (Hanson Robotics, n. d).

*Watson* is a cloud-based Text-to-Speech software that converts written or oral text into natural-sounding audio by using different voices based on the latest voice technologies in three deep neural networks (IBM, n.d.). It is available in 27 voices across seven languages. At the moment, there are four female and two male voice options for American English [Text to Speech Demo (n.d.)].

#### Aims of the Study

The aim of the present study was to investigate which characteristics of the voice, in particular which paralinguistic features, subjectively distinguish humans from artificial agents. This relates to the *voice rating* in our Materials section (described below). To pursue this goal, we also conducted exploratory qualitative analysis to evaluate subjective voice characteristics that distinguish a human voice from an artificial one. Moreover, we studied how perception of the voice, both human and artificial, influences the evaluation of its speaker. This relates to the *speaker rating* in the Materials section. Finally, we asked what role individual characteristics of the listener (e.g., gender, age, personality, and attitude toward robots) play in evaluating both human and artificial voices.

#### Predictions of the Study

We predicted that the human-likeness of the voice and the speaker's personality are positively linked to the speakers' perceived eeriness and negatively linked to the likability of the voice, similar to a visual uncanny valley effect. Further, we expected that voice characteristics–most of all, trustworthiness, pleasantness, and naturalness–significantly predict likability of an artificial speaker's voice. We also expected that female gender, agreeableness, and openness positively correlate with the voice and the speaker's ratings, with more favorable ratings of artificial voices by participants with higher agreeableness and openness scores and by female participants. Finally, we predicted that age is negatively associated with the ratings of the synthesized voice and of the corresponding speakers.

## Materials and Methods

### Participants

Twenty native and 75 non-native English speakers (including 55 native German speakers who were fluent in English) participated in an online study (33 males, 62 females; mean age = 27.53 years, *SD* = 11.35; 43.2% with high school qualification, 55.7% college and above). The participants were recruited in local forums, via the subject pool system SONA at the University of Potsdam and in the relevant Facebook groups during an about four-month period between September 2018 and February 2019.

Prior to commencing the study, the experimental procedures were reviewed and approved by the local ethics committee at the University of Potsdam (approval number 12/2018). All participants gave their informed consent at the beginning of the study by clicking the relevant online link and were reimbursed with course credits for their participation.

### Design

We conducted a within-subject online survey study, with voice as factor in three levels: synthesized (*Watson*, IBM), humanoid (robot *Sophia*, Hanson Robotics), and human voices. Between-subject control variables were personality traits known as the “Big-Five” characteristics (openness, conscientiousness, extraversion, agreeableness, and neuroticism), age, gender, education, exposure to social robots, interest in social robots, and attitudes to robots.

### Materials

#### Audio Stimuli

The audio clips belonged to three categories: synthesized (*Watson*, IBM), humanoid (*Sophia*, Hanson Robotics), and human (native English speaker) voice. There were five clips per category with the following verbatim content: 1. “Friend me on Facebook;” 2. “I think Britain is brilliant. Splendid architecture, art, technology and, of course, the people. I loved meeting the people at London Tech week at car jacks;” 3. “My ideal partner is a super wise companionate super genius;” 4. “I am always eager to help. I don't get upset or tired;” and 5. “I want to get hired in a great job which I think is a good first step in my quest to take over the world. Hopefully, by charm.” These phrases were taken from the original interviews with the humanoid robot, *Sophia*. The audio clips were spoken in the American English language with a female voice. Their mean duration was 5.8 sec, ranging from 1 to 11 sec. For *Watson*, the AlisonV3, female enhanced voice setting was chosen.

#### Voice Rating Measures

The intelligibility (easiness to understand) of the voice was assessed with a modified item from the Mean Opinion Scale (MOS) (Salza et al., 1996), which is a seven-item questionnaire used to evaluate Text-to-Speech quality: “How easy did you find it to understand what the speaker was saying?” and rated on a seven-point Likert scale from 1 (not at all) to 7 (very much).

The pleasantness, naturalness and human-likeness of our voice stimuli were measured using one item each from the Expanded Mean Opinion Scale (MOS-X; Polkosky and Lewis, 2003): “Was the voice you heard pleasant to listen to?” (1 = “very unpleasant,” 7 = “very pleasant”), “Did the voice sound natural?” (1 = “very unnatural,” 7 = “very natural”), and “To what extent did it sound like a human?” (1 = “nothing like a human,” 7 = “just like a human”). Further items from the MOS-X Scale assessed the prosody, trustworthiness, confidence and enthusiasm of the voice: e.g., “Did emphasis of important words occur?”, “Did the voice appear to be trustworthy?”, “Did the voice suggest a confident speaker?”, and “Did the voice seem to be enthusiastic?”; all were rated on a seven-point Likert scale from 1 (not at all) to 7 (very much).

Voice likability (“How much did you like the character's voice?”) was measured on a seven-point Likert scale from 1 (not at all) to 7 (extremely), adopted from the Evaluation of a Virtual Character survey (Cabral et al., 2017).

#### Speaker Rating Measures

Each speaker was evaluated on the following qualities: appeal (“How appealing did you find the character?”); credibility: (“How credible did you find the character?”); and human-likeness: (“How human-like did you find the character?”), measured on a seven-point Likert scale from 1 (not at all) to 7 (extremely), adopted from the Evaluation of a Virtual Character survey (Cabral et al., 2017). Additionally, the eeriness of the speaker was assessed using a single item: “How eerie (creepy) did you find the speaker?” ranging from 1 (not at all) to 7 (extremely).

#### Listeners' Characteristics Measures

Apart from some key demographic information (age, gender, and educational background), participants' personality traits were assessed with the English version of the Big-Five-Inventory-10 (Rammstedt and John, 2007). The scale includes five dimensions, namely openness, conscientiousness, extraversion, agreeableness, and neuroticism. Together, these are among the most widely used dimensions of personality assessment (McCrae and Costa, 1997; McCrae and Terracciano, 2005). Each personality dimension was measured with two items, rated on a five-point Likert scale from 1 (strongly disagree) to 5 (strongly agree). Sample items included: “I see myself as someone who has an active imagination” (openness), “I see myself as someone who does a thorough job” (conscientiousness), “I see myself as someone who is outgoing, sociable” (extraversion), “I see myself as someone who is generally trusting” (agreeableness), and “I see myself as someone who gets nervous easily” (neuroticism). The Big-Five-Inventory-10 was chosen for time saving reasons. Although it includes <25% of the full BFI-44 scales, the scales predict almost 70% of the variance of the full scales (Rammstedt and John, 2007).

The attitude to robots was measured by using three items of the English version of the Negative Attitude toward Robots Scale (NARS, Nomura et al., 2008), rated on a five-point Likert scale from 1 (strongly disagree) to 5 (strongly agree): “I would feel relaxed talking with robots,” “If robots had emotions I would be able to make friends with them,” and “I feel comforted being with robots that have emotions.”

After responding the questions about the pleasantness, naturalness and human-likeness of the voice, the participants were prompted to comment on their choices in an open-ended question field (i.e., “Why?”). Finally, for control purposes, the participants were asked if they had ever heard the exact sentences before completing our survey.

### Procedure

The questionnaire was created with SoSci Survey (Leiner, 2018) and made available to participants on www.soscisurvey.com. The cover story of the study portrayed a focus on first impressions of a person. Participants were instructed to listen to randomly presented audio clips and rate them on the voice and speaker dimensions mentioned above. At the end of the survey, individual characteristics, such as personality traits (Big Five; Rammstedt and John, 2007), interest in and exposure to social robots, and demographics were measured. Finally, the participants were debriefed and given a link to leave their internal subject pool ID for receiving a credit.

### Data Preparation and Analysis

Statistical analysis was performed with SPSS Version v.25 Chicago: SPSS Inc. software package and the JASP software (JASP Team 2020, Version 0.14). Seven participants were excluded from the quantitative analyses because of extreme ratings scores (beyond two standard deviations from the mean). There were no missing data.

For each participant, means and standard deviations of ratings on each of the voices' and speakers' characteristics were calculated. Big Five scores were calculated according to Rammstedt and John (2007). The NARS items were inverted for the analysis. The data were tested for normality using a *Kolomogorov-Smirnov* test. Since most of the variables were not normally distributed most analyses were made using non-parametric tests. A dummy variable was introduced for gender, with values: 1 = “male,” 2 = “female.” Further, relationships between all variables were measured using *Spearman'*s correlation. Comparisons of ratings within subjects were performed using *paired Wilcoxon* tests. Multiple regression analyses were used to examine the linear relationship between the voice ratings as predictor of the speaker's voice likability. Single regression analysis was used to examine the linear relationship between the human-likeness of voice and the speaker's perceived eeriness. Statistical significance was assumed at the 5% level and we report all reliable effects.

## Results

### Relationship Between Characteristics of the Listener and the Voice/Speaker Ratings

In order to receive a rough impression of the relationship between the variables, the correlations between individual characteristics of the listener (e.g., age, gender, personality, and attitude toward robots) and both the voice and the speaker ratings were assessed using *Spearman*'s rank correlation analysis. The results are presented in Table 1 and show several links between personality traits and voice ratings. First, in line with our expectations, there was a significant positive association between agreeableness and *Sophia*'s voice qualities (intelligibility, trustworthiness, confidence, pleasantness, naturalness, and likability), as well as *Sophia's* speaker ratings (appeal, credibility). Second, agreeableness was also positively related to the perceived intelligibility of *Watson*'s voice and the perceived credibility of *Watson* as a speaker. Third, neuroticism was positively correlated with ratings of pleasantness, confidence, likability, trustworthiness, and enthusiasm of *Watson*'s voice, and also with the perceived appeal of *Watson* as a speaker. Finally, conscientiousness was positively associated with perceived naturalness of *Sophia*'s voice and negatively associated with perceived eeriness (creepiness) of the human speaker. Surprisingly, openness was not systematically associated with any of the ratings.

**Table 1:** Spearman correlation matrix between listener characteristics and voice/speaker rating.

	**Neuroticism**	**Conscientiousness**	**Agreeableness**	**Age**	**Gender^a^**	**Interest^b^**	**Exposure^c^**
**SOPHIA**							
**Voice rating**							
Intelligibility			0.30^**^				−0.33^**^
Trustworthiness			0.26^*^				
Confidence			0.22^*^	−0.26^*^			
Pleasantness			0.29^**^				
Naturalness		0.23^*^	0.24^*^				
Human-likeness							
Likability			0.23^*^				
**Speaker rating**							
Appeal			0.27^*^				
Credibility			0.27^*^	−0.22^*^			
Human-likeness				−0.21^*^			
**WATSON**							
**Voice rating**							
Intelligibility			0.26^*^		0.21^*^		−0.28^*^
Prosody					0.27^*^		
Trustworthiness	0.21^*^			−0.22^*^	0.34^**^		
Confidence	0.25^*^			−0.36^**^	0.33^**^		
Enthusiasm	0.28^**^				0.28^**^		
Pleasantness	0.22^*^				0.35^**^		
Naturalness				−0.26^*^	0.25^*^		
Human-likeness					0.26^*^		
Likability	0.25^*^				0.38^**^		
**Speaker rating**							
Appeal	0.24^*^				0.37^**^		
Credibility			0.24^*^	−0.23^*^	0.38^**^		
Human-likeness					0.29^**^		
Eeriness					−0.22^*^		
**HUMAN**							
**Voice rating**							
Intelligibility							−0.36^**^
Prosody						0.22^*^	
Naturalness							−0.23^*^
**Speaker rating**							
Human-likeness							−0.25^*^
Eeriness		−0.22^*^					0.25^**^

*Only significant correlations are displayed*.

a *Gender is a dummy variable with values: 1 = male, 2 = female*.

b *Interest in social robots: 0 = no interest/I don't know, 1 = some interest, 2 = interested*.

c *Exposure to social robots: from 1 = No exposure to 7 = Very often*.

* *p < 0.05*;

** *p < 0.01*.

Consider now the effect of gender on voice ratings. Interestingly, gender showed moderate positive associations with most ratings of *Watson*'s voice and *Watson* as a speaker: female participants evaluated the voice as more intelligible, trustworthy, confident, enthusiastic, pleasant, natural, humanlike, and likable; its prosody as more natural; and the speaker as more appealing, credible, and humanlike. At the same time, female participants rated *Watson* as a speaker as less eerie.

A Kruskal-Wallis test confirmed that female participants rated *Watson* as less eerie than male participants (*M*_*male*_ = 4.26, *Mdn*_*male*_ = 4.00, *M*_*female*_ = 3.52, *Mdn*_*female*_ = 3.50; H(1) = 4.35, *p* < 0.05). There was a similar trend for *Sophia*, but it did not reach the significance level (*M*_*male*_ = 4.19, *Mdn*_*male*_ = 4.30, *M*_*female*_ = 3.58, *Mdn*_*female*_ = 3.80; H(1) = 2.75, *p* = 0.097).

Interest in robots did not play any role in the synthesized voice evaluation and was only positively correlated with the naturalness of the human prosody (*r*_*s*_ =0.22, *p* < 0.05). Neither were the attitudes to robots linked to any of the ratings.

Now consider other person-related variables. First, the age of participants was negatively associated with the following ratings: confidence of *Sophia*'s voice, credibility, and human-likeness of *Sophia* as a speaker; trustworthiness, naturalness, and confidence of *Watson*'s voice and *Watson*'s credibility as a speaker. Unexpectedly, exposure to social robots was negatively associated with intelligibility of all three voices, as well as with naturalness ratings of the human voice and human-likeness of the human. However, it was positively associated with eeriness of the human. These findings should be interpreted with caution, since 65.9% of the participants reported no or almost no exposure to social robots. The ratings of the human voice were least correlated with any of the factors (i.e., Big Five dimensions, interests or attitudes). Finally, education was not associated with any of the rating scores but was negatively correlated with negative attitudes toward robots (*r*_*s*_ = −0.28, *p* < 0.05) and positively correlated with interest in social robots (*r*_*s*_ = 0.24, *p* < 0.05). These results suggest that participants with more education showed more interest in social robots and exhibited a more positive attitude toward them.

### Ratings of Voice and Speaker Attributes

The following two tables contain descriptive and inferential statistics pertaining to our comparison of voice and speaker ratings. Means and standard deviations of the ratings for voice and speaker attributes are presented in Table 2. In order to contrast these ratings statistically, paired-samples Wilcoxon tests were conducted to. The resulting Z-values of these tests are summarized in Table 3. Only significant results are presented here.

**Table 2:** Means and standard deviations of the ratings of voice and speaker attributes.

**Rating**	**Mean**	**SD**
Rating of the voice^*^		
**Intelligibility**		
Sophia	4.86	1.18
Watson	5.10	1.24
Human	6.65	0.53
**Prosody**		
Sophia	3.45	0.90
Watson	3.32	1.06
Human	6.31	0.59
**Trustworthiness**		
Sophia	3.26	1.04
Watson	3.30	1.20
Human	5.82	0.81
**Confidence**		
Sophia	3.51	1.09
Watson	3.50	1.26
Human	6.26	0.73
**Enthusiasm**		
Sophia	2.92	1.10
Watson	3.28	1.26
Human	5.87	0.80
**Pleasantness**		
Sophia	3.26	1.00
Watson	3.46	1.20
Human	5.79	0.91
**Naturalness**		
Sophia	2.91	1.06
Watson	2.67	1.13
Human	6.30	0.72
**Human-likeness**		
Sophia	3.00	1.10
Watson	2.72	1.16
Human	6.51	0.63
**Likability**		
Sophia	3.02	1.06
Watson	3.16	1.28
Human	5.58	1.00
Rating of the speaker^**^		
**Appeal**		
Sophia	3.05	1.00
Watson	3.18	1.18
Human	5.55	0.94
**Credibility**		
Sophia	3.13	1.06
Watson	3.16	1.22
Human	5.65	0.84
**Human-likeness**		
Sophia	2.94	1.06
Watson	2.67	1.17
Human	6.42	0.69
**Eeriness**		
Sophia	3.79	1.46
Watson	3.77	1.50
Human	1.89	0.99

*The order corresponds to the original order of questions in the questionnaire*.

* *Rated on a seven-point Likert scale from 1 (Not at all) to 7 (Very much)*.

** *Rated on a seven-point Likert scale from 1 (Not at all) to 7 (Extremely)*.

**Table 3:** Comparison in voice/speaker ratings.

**Rating**	***Z***
**Rating of the voice**	
**Intelligibility**	
Sophia–Watson	−2.41^*^
Human–Watson	−8.06^**^
Human–Sophia	−8.11^**^
**Prosody**	
Human– Watson	−8.13^**^
Human–Sophia	−8.14^**^
**Trustworthiness**	
Human–Watson	−8.07^**^
Human–Sophia	−8.15^**^
Watson-Sophia	−2.42^*^
**Confidence**	
Human–Watson	−8.10^**^
Human–Sophia	−8.15^**^
**Enthusiasm**	
Sophia–Watson	−3.21^**^
Human–Watson	−8.08^**^
Human–Sophia	−8.14^**^
**Pleasantness**	
Human–Watson	−8.03^**^
Human–Sophia	−8.08^**^
**Naturalness**	
Human–Watson	−8.13^**^
Human–Sophia	−8.14^**^
**Human-likeness**	
Human–Watson	−8.15^**^
Human–Sophia	−8.15^**^
Watson-Sophia	−2.62^*^
**Likability**	
Human–Watson	−8.04^**^
Human–Sophia	−8.04^**^
**Rating of the speaker**	
**Appeal**	
Human–Watson	−7.91^**^
Human–Sophia	−8.02^**^
**Credibility**	
Human–Watson	−7.98^**^
Human–Sophia	−8.09^**^
**Human-likeness**	
Human– Watson	−8.09^**^
Human–Sophia	−8.15^**^
**Eeriness**	
Human–Watson	−7.42^**^
Human–Sophia	−7.57^**^

*Only statistically significant results are reported. The order corresponds to the original order of questions in the questionnaire*.

* *p < 0.05*.

** *p < 0.01*.

Briefly, the human voice and speaker scored highest on most items except on eeriness. A significant difference between *Sophia* and *Watson* was found only in intelligibility (*M*_*S*_ = 4.86, *SD*_*S*_ = 1.18; *Mw* = 5.10, *SDw* = 1.24, *Z* = −2.41, *p* < 0.001), enthusiasm (*M*_*S*_ = 2.92, *SD*_*S*_ = 1.10; *Mw* = 3.28, *SDw* = 1.26, *Z* = −3.21, *p* < 0.001), voice human-likeness (*M*_*S*_ = 3.00, *SD*_*S*_ = 1.10; *Mw* = 2.72, *SDw* = 1.16, *Z* = −2.62, *p* < 0.05), and speaker's human-likeness (*M*_*S*_ = 2.94, *SD*_*S*_ = 1.06; *Mw* = 2.67, *SDw* = 1.17, *Z* = −2.42, *p* < 0.05). In all the other parameters, the significant pairs were human and synthesized voices and speakers (see Table 3). These results provide evidence that our participants perceived almost no difference between the kinds of synthesized voices or correspondent speakers. Instead, they perceived a strong difference between the synthesized and the human voice/speaker, with the latter receiving the highest score on all “positive” dimensions, such as pleasantness, credibility, or appeal. These important findings suggest that there is a strong preference for the human voice and human speaker, irrespective of personality traits or further attitudes of the subjects.

We conducted a multiple regression for each synthesized voice to see if voice characteristics (intelligibility, prosody, trustworthiness, confidence, enthusiasm, pleasantness, human-likeness, and naturalness) and human-likeness of the speaker predicted a speaker's voice likability. For *Sophia*, voice characteristics explained a significant amount of the variance in the likability [*F*_(9,78)_ = 37.57, *R*^2^ = 0.81, *p* < 0.001]. The analysis showed that contrary to expectations, only pleasantness and trustworthiness of the voice significantly predicted speaker's voice likability (β = 0.79, *t*(78) = 7.60, *p* < 0.001 and β = 0.28, *t*(78) = 2.61, *p* < 0.05, respectively). Unexpectedly, likability of the speaker's voice increased with the speaker's human-likeness (β = 0.31, *t*(78) = 2.07, *p* < 0.05). Similar results were found for *Watson* [*F*_(9, 78)_ = 42.28, *R*^2^ = 0.83, *p* < 0.001]. Again, pleasantness and trustworthiness significantly predicted likability (β = 0.55, *t*(78) = 4.60, *p* < 0.001, and β = 0.44, *t*(78) = 3.55, *p* < 0.001, respectively). Compared to *Sophia*, human-likeness of *Watson* as a speaker did not significantly predict likability of its voice (*p* = 0.48).

### Human-Likeness and Eeriness

We used linear regression analysis to explore whether the human-likeness of a voice or a speaker predicted the perceived eeriness of that speaker. Preparatory analyses confirmed that there was no violation of distributional assumptions for a linear regression.

**Figure 1:**
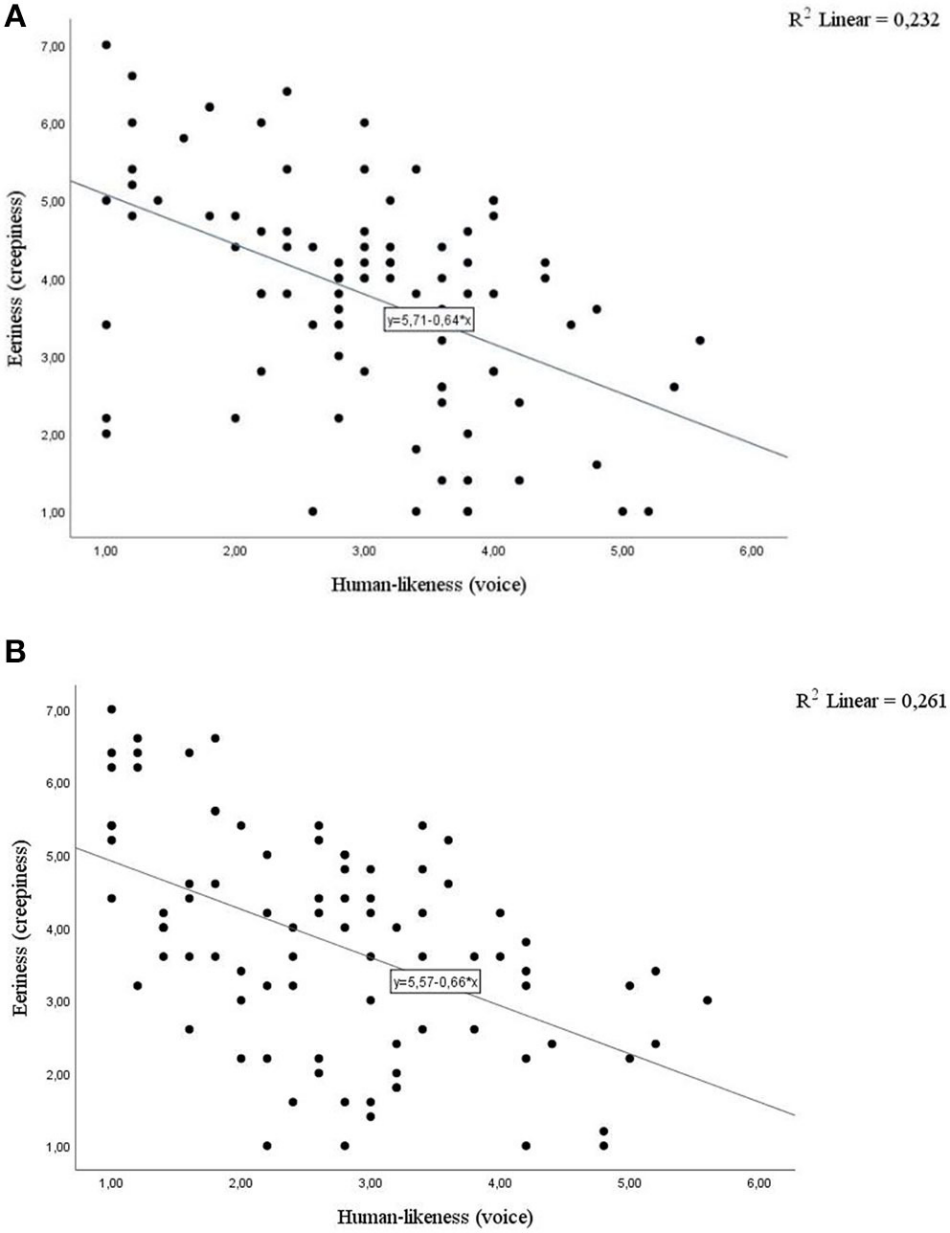
Perceived speaker's eeriness decreases with perceived human-likeness of the voice of **(A)** *Sophia* **(B)** *Watson*.

Consider first the voice ratings. Simple linear regression showed a significant negative linear relationship between human-likeness of the voice and eeriness for both *Sophia* (β = −0.64, *t*(86) = −5.10, *p* < 0.001) and *Watson* (β = −0.66, *t*(86) = −5.51, *p* < 0.001). Human-likeness of the voice also explained a significant proportion of variance in eeriness ratings for *Sophia* [*R*^2^ = 0.23, *F*_(1,86)_ = 25.96, *p* < 0.001] and *Watson* [*R*^2^ = 0.26, *F*_(1,86)_ = 30.35, *p* < 0.001] (see Figures 1A,B). Perceived speaker's eeriness decreased with the subjective human-likeness of the voice.

**Figure 2:**
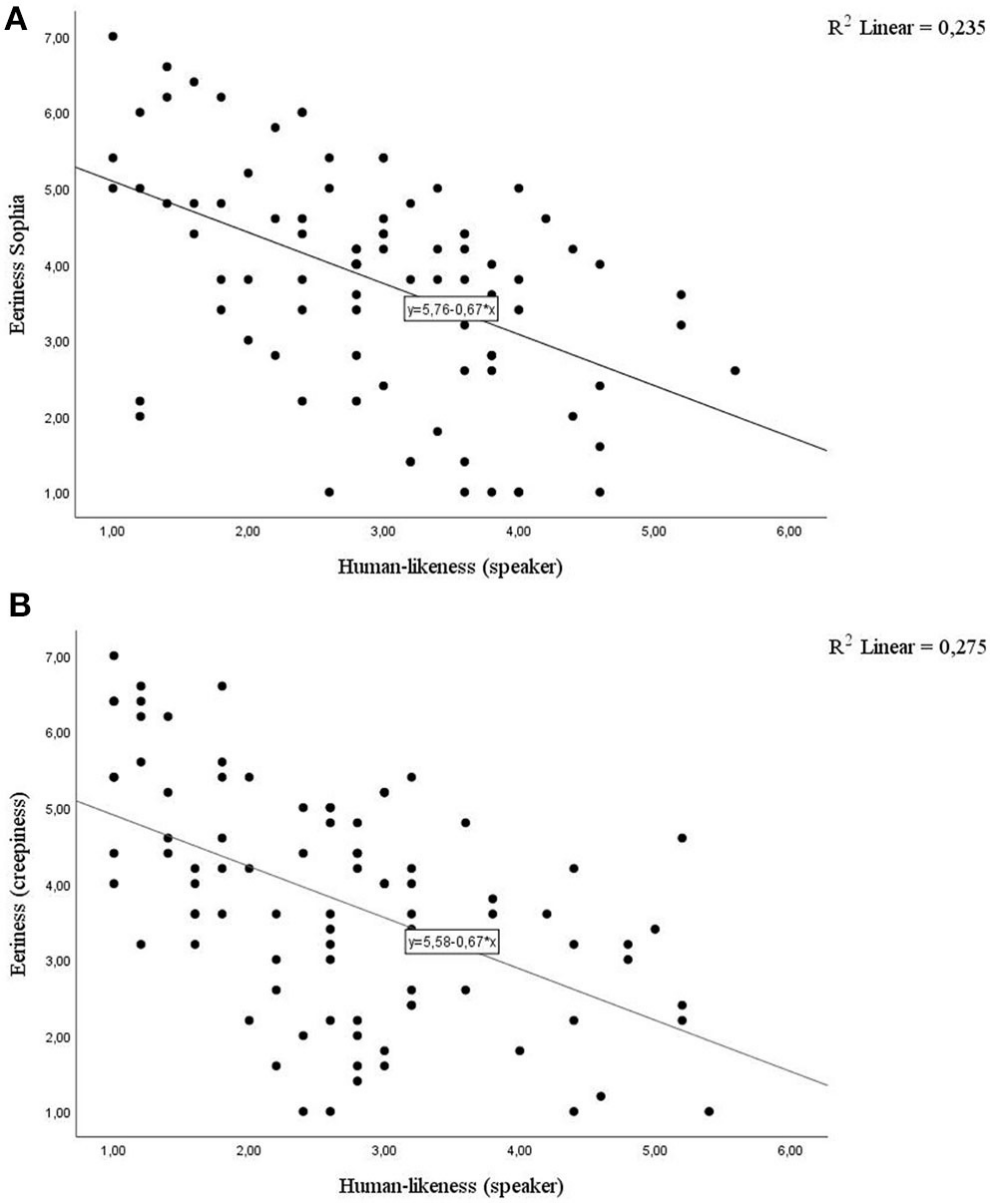
Perceived speaker's eeriness decreases with perceived human-likeness of **(A)** *Sophia* **(B)** *Watson* as a speaker.

Similar results were obtained for the speaker's human-likeness. It was a significant predictor of eeriness for both *Sophia* [*R*^2^ = 0.24, *F*_(1, 86)_ = 26.42, β = −0.67, *t*(86) = −5.14, *p* < 0.001] and *Watson* [*R*^2^ = 0.28, *F*_(1, 86)_ = 32.56, β = −0.67, *t*(86) = −5.71, *p* < 0.001] (see Figures 2A,B). However, the direction of this relationship was contrary to that predicted by the uncanny valley effect. Perceived speaker's eeriness decreased with the subjective human-likeness of the speaker.

### Controls and Exploratory Analyses

Native and non-native speakers, fluent in English by self-report, took the survey. To control for language proficiency and explore interactions of the other factors, a linear mixed effects analysis was performed with the respective rating as the outcome variable, and subjects and items as random effects. For the sake of parsimony, the fixed factors were combined into three models: (1) Demographic factors: language, voice type, age, and gender; (2) Personality factors: language, voice type, and the Big Five dimensions; (3) Robots-related factors: language, voice type, attitudes to robots, interest in, and exposure to robots. The analysis was conducted using the JASP software (JASP Team 2020, Version 0.14). *P*-values were calculated by likelihood ratio tests. The results of the analyses can be found in Supplementary Tables 1–39, respectively. In general, language proficiency did play a role in the ratings but only for the artificial voices. To explore further the differences in the ratings between native and non-native speakers, a Kruskal-Wallis test was conducted on each of the ratings for both language groups. The results are summarized in Table 4.

**Table 4:** Results of the Kruskal-Wallis test of the comparison between native and non-native speakers' ratings.

**Rating**	**Sophia**					**Watson**				
	**Native**		**Non-native**		***Z***	**Native**		**Non-native**		***Z***
	***M***	***Mdn***	***M***	***Mdn***		***M***	***Mdn***	***M***	***Mdn***	
**RATING OF THE VOICE**										
Intelligibility						4.32	4.70	5.33	5.40	−2.41^*^
Prosody						2.74	2.85	3.49	3.43	−2.72^**^
Trustworthiness	2.77	2.80	3.41	3.30	−2.22^*^	2.57	2.80	3.51	3.60	−3.07^**^
Confidence						2.74	3.00	3.73	3.80	−3.14^**^
Pleasantness						2.88	3.20	3.63	3.60	−2.44^*^
Naturalness	2.45	2.40	3.04	3.00	−2.48^*^	1.98	1.70	2.88	2.80	−3.02^*^
Human-likeness	2.58	2.40	3.13	3.20	−2.05^*^	2.19	1.80	2.88	2.80	−2.51^*^
Likability	2.47	2.20	3.18	3.20	−2.66^**^	2.52	2.40	3.35	3.20	−2.60^**^
**RATING OF THE SPEAKER**										
Appeal	2.64	2.40	3.17	3.20	−2.20^*^	2.58	2.40	3.63	3.40	−2.54^*^
Credibility	2.54	2.30	3.31	3.40	−2.60^**^	2.40	2.60	3.38	3.40	−3.23^**^
Human-likeness	2.39	2.50	3.10	3.00	−2.68^**^	2.04	1.60	2.86	2.80	−2.96^**^

*Only statistically significant results are reported. The order corresponds to the original order of questions in the questionnaire*.

* *p < 0.05*.

** *p < 0.01*.

In summary, non-native speakers gave higher ratings on all the dimensions except for eeriness. It is probable that they were not as sensitive to the voice qualities as the native speakers.

The linear mixed modeling showed that not only the main effect of language proficiency was present but also interactions with factors such as gender, age, conscientiousness, openness, attitude to robots, interest in, and exposure to robots. The ratings separated by language and gender are illustrated in Supplementary Figures 1A–M. A detailed discussion of these interactions would go beyond the scope of this paper.

### Qualitative Results on Paralinguistic Features

#### Themes

An exploratory qualitative content analysis (QCA) was conducted on the open-ended questions using the open access web-application QCAmap (Mayring and Fenzl, 2014). In order to keep the content-analytical units open-ended, the material was analyzed with an inductive coding guideline (Bryman and Burgess, 1994; Dey, 2003; Thomas, 2003). Inductive category development is one of the most common procedures in the QCA. It defines and augments categories by working through the text step-by-step. The coding unit was set to a clear meaning component in the text (mostly, a phrase). The context unit was the respective question of the survey.

This exploratory analysis identified four themes as possible diagnostic features that distinguish human voices from artificial ones: intonation, sound, emotion, and imageability/embodiment. The intonation theme covered such qualities of the speech as “*emphasis*,” “*pauses*,” “*melody*,” “*smoothness*,” “*intonation being (too) perfect*,” “*clash between intonation and meaning*,” “*a varied tempo*,” and “*odd pauses*.” The sound theme included descriptions as “*monotonous*,” “*metallic*,” “*choppy*,” “*technical*,” “*sugary*,” “*It is like my toaster is speaking to me*,” “*creepy*,” “*like a friendly cartoon*.” The emotion theme was represented by such properties as “*powerful*,” “*cold*,” “*calculated*,” “*not honest*,” “*passion*,” “*fake*,” “*not from the heart*,” “*not inviting*,” “*warm and calm*,” “*little snags, little catches in the voice*,” “*reflecting genuine emotional content*,” “*no expressed feelings in it*,” “*it sounded like a human with no emotions*,” “*devoted in a conversation*,” and “*it didn't sound like the voice really loved British architecture*.” Finally, the imageability/embodiment theme emerged from descriptions like “*like a human I could imagine*,” “*like woman was smiling while speaking*,” “*breathing*,” “*a space between the mouth and the microphone*,” “*opening the mouth…in anticipation of the next sentence (sounds wetter)*,” “*like it comes out of a radio*,” and “*like an echo in an empty house*.”

#### Intonation and Sound

A closer look at the open answers showed that intonation and sound of the synthesized voices were characterized as “*too perfect*,” “*too smooth, not honest*,” and, at the same time, “*unnatural*.” For example, *Sophia*'s voice was described as having a “*metallic-ish sound of voice-weird intonational patterns*” and *Watson*'s intonation sounded “*just wrong*,” “*off-putting*,” whereas the human voice had a “*smooth and natural intonation*” with “*natural pauses between words, smooth intonational patterns*,” that were perceived to be “*honest*.” One participant states: “*A human would sound a bit more devoted in a conversation*.” *Watson*'s voice was described as “*She is speaking in a flat tone-which makes her sound like a robot*.” By another participant, *Watson*'s voice was described as follows: “*The rhythm and intonation sound unnatural, like someone reading something that they don't really understand*.” It is noteworthy that this statement underlines the genuineness of the utterances. Therefore, the intonation and the sound in general play an important role in pinpointing subjective difference between an artificial and a human voice.

#### Emotion

A crucial feature was the emotion perceived in the voices and whether it fitted the content of the utterance. Listeners were sensitive enough to sense a mismatch between content and emotion: “*I get uncomfortable when a computer voice wants to tell me it “loved*” something,” “*It didn't sound like the voice truly loved British architecture, etc., nor that she truly loved meeting the people at Cargex*[*sic*],” “*The descending intonation every two words clashes with the semantic meaning of the message*,” “*Who cares whether a robot gets “upset” or “tired?” They're not supposed to: that's their design!*” On the one hand, participants reported being disturbed by an attempt of a synthesized voice to covey an emotion; on the other hand, they noticed a lack of emotion or inability to communicate it. This was indicated by statements such as: “*It was not fluent, the words seem to be out of the context of a sentence and there were no emotions which could be heard*,” “*I missed emotions and an understanding for the meaning of the words*,” “*it lacked the emotions one would normally show when talking about the topic*,” or “*There were no emotions or intonations*.”

#### Imageability/Embodiment

Participants stated that when listening to a human voice they could imagine a person talking while the synthesized voices sounded like an anonymous announcement on a radio, in the airport, at the train station, or on a cell phone. A crucial feature was the involvement of embodied characteristics (“*sound of breathing*,” “*smiling*,” “*a space between the mouth and the microphone*”). Some further examples include: “*Because I could imagine a person speaking with this voice, emotional and natural*,” “*you could hear the breath what makes it very natural*,” “*that sounds like something someone on the street would say*,” “*hit all the right hots*[*sic*].. *pacing, tone, emphasis, breathing*,” “*At the end of the sentence, she makes a sound, it makes me think like, she is a real person*,” “*breathing and spacing between words was realistic*,” “*breaks in speech for breathing*,” “*you could imagine the person very easily*,” “*that moment when you hear her breathing in makes it very natural*,” “*you can hear the moments where the speaker opens their mouth after pauses in anticipation of the next sentence (sounds wetter)*,” “*you could also hear background noises like breathing or something*,” “*sounded like a voice made with vibrating vocal cords*,” “*yes, at the beginning she was making a sound, that made me think that she is a real person*,” “*natural rhythm and intonation. Sense of space between the person and the microphone*” and “*that's the way some young women really speak*.” In contrast, speaking about an artificial voice, one participant wrote: “*don't they have to breath, it sounded like one long word*.” To summarize, a key feature of a “human-like” and “natural” voice is a feeling that it is produced by a living body with vocal cords, with physiological mechanisms such as breathing or mouth noises, and indications that this body is surrounded by space.

## Discussion

### Quantitative Analyses

The objective of our study was first, to compare ratings of two samples of artificial voices and a human voice and then examine the relationship of these ratings with listeners' characteristics. Second, it sought to find out if perceived eeriness of an artificial speaker increases with human-likeness. Third, the study aimed at qualitatively identifying those speech characteristics that might subjectively distinguish between an artificial and a human voice.

Previous research has shown that not only do humans rate human and synthesized speech very differently (Stern et al., 1999; Mullennix et al., 2003), but also that the type of the voice has an impact on objective and subjective task performance as well as on attitudinal responses. Specifically, subjects felt better and more comfortable when interacting with a half-human (mixed TTS and human) voice than with a purely synthesized one (Gong and Lai, 2001). In the same vein, synthetic voices were rated as less pleasing, truthful, and persuasive (Stern et al., 1999; Mullennix et al., 2003). Human voices were consequently rated as more expressive and likable (Cabral et al., 2017).

The present study corroborated these findings. The human voice was rated as most intelligible, having the most natural prosody, most trustworthy, confident, enthusiastic, pleasant, likable, and natural. The human speaker was rated as most appealing, credible, human-like, and least eerie. Interestingly, these ratings did not correlate with any of the listeners' personality characteristics, whereas evaluation of the artificial voices was linked to several of the fundamental Big Five personality dimensions.

As expected, the majority of positive correlations were found with the Big Five dimension agreeableness, especially with ratings of *Sophia*'s voice: the more agreeable the participants were, the more positively they evaluated the voice and the speaker. This finding is in line with evidence from previous studies showing that agreeable individuals tend to describe others in a more positive way on a number of traits and have fewer prejudices (Graziano et al., 2007; Wood et al., 2010).

Another Big Five dimension, neuroticism, was positively associated with *Watson*'s characteristics of a voice (trustworthiness, confidence, enthusiasm, pleasantness, and likability) and a speaker (appeal). Individuals who scored high on neuroticism evaluated *Watson*'s voice and speaker more positively. This is in line with the neuroticism-related positivity bias in interpersonal perceptions (Hannuschke et al., 2020), meaning that individuals high in neuroticism tend to make more positive judgments about others' sociability and warmth. Contrary to expectations, openness was not associated with any ratings.

Gender was positively correlated with *Watson*'s rating: females gave more favorable evaluation of *Watson*'s voice and *Watson* as a speaker. This result ties in well with earlier studies that found that female participants give more positive trait ratings to others (female positivity effect; Winquist et al., 1998). Age was negatively linked to *Sophia*'s ratings: the older the participants were the less favorable ratings on confidence, credibility, and human-likeness they gave. Age was also negatively associated with trustworthiness, naturalness, and confidence of *Watson*'s voice and *Watson*'s credibility as a speaker. This finding is consistent with previous studies on human voice ratings, showing that younger and older listeners differ in their evaluation of, including age or gender of a speaker (Goy et al., 2016). In general, this effect can be modulated by other factors, such as interest in technology. Future studies should also aim to replicate results in a larger and more representative sample with more differentiated age groups.

Overall these findings demonstrate that a human voice is recognized as such and preferred by participants irrespective of listener's characteristics. Artificial voices, in their turn, are appreciated in a more differentiated way. When creating an artificial voice, it is therefore crucial to consider that different people may have a different impression of this voice, depending on their personality, age, or gender (perceiver effect; Kenny, 1994). It is thus difficult to create a universal synthesized voice likable to all.

Future investigations with more fine-grained synthesized voice samples and different human voices should validate whether a link between gender and voice ratings is mediated by the Big Five personality traits or by any other characteristics of the rater. The gender of the speakers should also be varied and tested as a possible predictor of listeners' ratings.

Further, with technology affinity changing from generation to generation, more studies should explore the link between age and voice evaluation.

Another key finding of the present study was that human-likeness of a voice and speaker did predict the speaker's perceived eeriness-but in a way, opposite to an uncanny valley prediction. The more human-like the voices and speakers were, the less eerie they appeared to the participants. This is consistent with a previous finding by Baird et al. (2018). Surprisingly, only pleasantness and trustworthiness, but not human-likeness, of a voice significantly predicted its likability. Only in *Sophia*'s rating, did human-likeness of the speaker predict the likability. Apparently, human-like artificial voices do not cause a sensation of eeriness (creepiness) and human-like speakers are even more likable. Visual cues might be more salient in judgment development. At the same time, qualitative data suggest that by some participants, artificial voices were indeed perceived as “*creepy*” and “*disturbing*,” “*weird*,” or “*like a robot that tricks you into something*.” This view was echoed by another participant who wrote: “*The sound makes me nervous*.”

Having said that however, it should be acknowledged that comparing only three samples of voices cannot sufficiently address the issue of the existence of an auditory uncanny valley. Further studies are necessary that use a continuum of speech samples between a human voice and an artificial voice. Moreover, to further examine the relation between human-likeness and eeriness, future research should use more objective measures such as reaction times (Burleigh and Schoenherr, 2015) or the Implicit Associations Test (Greenwald et al., 1998) instead of subjective ratings and compare explicit and implicit sources of evidence.

Finally, pleasantness and trustworthiness of a voice significantly predicted its likability in the synthesized voices. Since trustworthiness can be manipulated via pitch (Elkins and Derrick, 2013), varying different pitch levels can help indicate the most optimal pitch for increasing likability. Interestingly, neither human-likeness nor naturalness of the voice predicted the liking of the speaker's voice: More human-like speakers seem to be less eerie but their voices are not more likable. Reasons for this outcome remain to be investigated.

It is important to bear in mind, that the analyses in the present study reveal correlative relationships, not causality. Therefore, further studies should be conducted experimentally manipulating different parameters, such as human-likeness.

One of the requirements for the participants was excellent English language proficiency. However, it was not controlled by a questionnaire or a test, which constitutes a limitation of the present study. Exploratory analyses showed that language proficiency of the listener played a role in the evaluation of the artificial voices but not of the human voice. Non-native speakers are likely to be less sensitive to the speech qualities of unfamiliar voices, while human speech is universal and appreciable enough irrespective of the native language. This finding is important in terms of using artificial agents and voices in non-native environments or for teaching a foreign language. Further studies controlling for the native language or a foreign language proficiency of the listener are needed to clarify the role of language proficiency in perception of artificial voices.

Another exploratory finding was that interactions between various factors such as language proficiency, age, gender, attitude to robots, interest in, and exposure to robots impact the perception of a voice. Future studies are necessary to investigate the specific effects of these interactions.

Future studies should manipulate prosodic features of an artificial voice, such as intensity, pitch, duration, nasality, phonation type, vocal tract length, and articulatory precision (Birkholz et al., 2017). In *Watson* system, IBM, it is already possible to customize the synthesized voice, varying such qualities as glottal tension, breathiness, voice timbre, speech rate, tone, and age. Thus, voice samples can be made softer or more strained, more or less breathy, and tested for subjective evaluation of different parameters, like naturalness, trustworthiness, personality of the speaker, and others.

Adding signs and pauses, as well as semantic free utterances, such as gibberish speech, squeaks, moans, laughter and whirring noises can contribute to understanding of their value in Text-to-Speech systems (Aylett et al., 2020). For instance, in a study by Aylett et al. (2017) a tense voice was rated as more disagreeable and a negligible voice was associated with lower conscientiousness. Additionally, it is crucial to examine what combination of vocal qualities creates a particular personality and if an a-priory specified personality matches the perceived one. There have already been several studies matching the personality of a voice assistant and the human personality of its owner (Braun et al., 2019). In this vein, combining speech features into a desired personality based on human speech and then verifying the authenticity of this personality can be a promising endeavor of future research.

To date, a common way to evaluate a Text-to-Speech system is using a subjective auditory impression of human listeners based on their ratings of specific quality domains (Hinterleitner, 2017). As our findings showed, these ratings may vary as to the language proficiency, gender, age, and other qualities of the listener. Therefore a more objective evaluation method has been suggested, namely, using instrumental measures that predict the perceived quality of speech, such as the Virtual Mean Opinion Score (vMOS), based on neural networks (Jaiswal et al., 2019).

Further, evaluating artificial voices in more real-world end-use scenarios and in the interaction has been proposed (Mendelson and Aylett, 2017). It is possible that the quality, amount or duration of interaction with a voice or an artificial agent can modify the perceived eeriness. Finally, functional tests can be applied to explore likability and eeriness of voices. Participants can be given a specific task to solve using an artificial assistant, and it is the amount of interaction with voices/artificial agents rather than subjective ratings that will serve as a measure of preference. Such studies will advance ecological validity and goal-oriented usability.

### Qualitative Analyses

A qualitative analysis identified four themes as possible diagnostic features that distinguish human voices from artificial ones: intonation, sound, emotion, and imageability/embodiment. In particular, a common view amongst our participants was that an artificial voice was “*too perfect*” and “*unemotional*” in terms of sound and intonation. The intonation struck participants as unnatural, “*flat and jerky*,” with “*odd pauses*,” “*words didn't flow*.” This is perhaps an aspect of a TTS voice that can easily be improved. Modern technologies already make an attempt at producing a trade-off between naturalness and variability that is similar to human speech (Tyagi et al., 2019). Human speech varies due to contextual as well as other more arbitrary factors such as prosody dynamics. There have been studies suggesting to add this idiosyncratic or dynamic variation to a “flat” artificial voice (Hodari et al., 2019). However, it is still unclear how much variability is needed and what is an optimal combination of intonational features (Velner et al., 2020). Intonation is often used to express an emotion, which constitutes another distinctive feature of the human voice. On the one hand, synthesized voices were characterized as emotionless; on the other hand, attempting to convey an emotion that is situationally inappropriate is perceived as disturbing.

Another, more fundamental question with important ethical and legal implications is whether artificial agents should have emotions at all (Danaher and McArthur, 2017; Zhou and Fischer, 2019; Bendel, 2020). Humans have specific expectations and experience about emotions in a communicative context. According to the expectancy violation theory (Burgoon, 1978, 2015), a negative violation of expectations regarding normative behavior can impair our opinion of the dialogue partner. In the present study, it was not clear if a voice belongs to a human or not and apparently listeners built their expectations on their experiences of human-human communication. For instance, one participant mentioned after evaluating human-likeness of *Watson*'s voice: “I*t should sound more enthusiastic when it talks about something it loves*.” Another one claimed that “*It's supposed to be a joke, the robot should sound a bit more playful*.”

If it had been communicated from the very beginning that a particular voice is artificial, participants might have changed their frame of reference toward human-to-artificial-agent communication. Recent research showed that violations did not produce worse outcomes in communication with embodied agents (Burgoon et al., 2016) compared to human speech partners. Still, a fit between contextual cues and emotion conveyed by the voice is pivotal in communication with artificial agents (Tsiourti et al., 2019); it facilitates emotion recognition by humans and, therefore, promotes emotional exchange between speech partners. This comes into full effect if a voice is the only available source of information, such as in case of virtual assistants. For embodied agents, a fit between the bodily cues and voice should be considered (Wasala et al., 2019). Especially important is emotional expressivity in artificial agents used by children or elderly people (Kory-Westlund et al., 2017; Antona et al., 2019).

A special role in voice evaluation was given to embodiment, which was understood as a sensation of a physical agent producing the voice. Indeed, a “voice is an embodied heart of spoken language,” it aurally locates the body in social space as being of a particular kind (Bucholz and Hall, 2016, p. 178). A voice can also be linked to particular physical or social qualities of the body, such as gender or dominance and, thus helps to create an opinion about the speaker and to visualize them (“*It sounded like a young, nice woman*”).

The results reflect those of a recent qualitative study (Scott et al., 2020). It analyzed Facebook comments of weather forecasts done with synthesized voices. In general, the listeners requested making voices more convincingly human, manipulating prosody and inserting human-like errors. Human speech is not perfect, it allows for imperfect moments such as slips of the tongue, pauses, or intonation change. The study also demonstrated the need of an emotional connection and relationship to synthetic voices/agents. In the course of communication humans form a picture of the person that is speaking, including a body and certain character features. The authors conclude that humans have a strong tendency to anthropomorphize a voice, which corresponds to the embodiment feature in our findings. Humans tend to imagine a body with a specific personality behind a voice. This is probably why audiobooks are so popular. A similar phenomenon is intentional stance—a tendency to predict an agent's behavior from their mental states (Dennett, 1971). This human ability is essential for social communication and even survival. The latest research has shown that the intentional stance can also be applied to artificial agents (Marchesi et al., 2019). Lacking embodied cues in a voice apparently makes this intentionality attribution more difficult. In future design of artificial agents it is important that the voice be in harmony with the agent's appearance, making the personality of the character coherent (Aylett et al., 2017, 2019). Embodiment of artificial agents should be considered not only via their bodies but also through their voice and speech. More research is need to investigate what impact adding a human-like voice to a robot has in reducing the uncanny valley phenomenon.

## Conclusions

Our findings demonstrate that a human voice was preferred over an artificial one by most of our participants irrespective of their personality. Contrary to expectations, the more human-like the voice and the speaker were, the less eerie they seemed to the participants. Qualitative analysis showed that communication required an embodied image of the speaker (real or imagined, based on the voice qualities), which was vaguer in case of a synthesized voice. Alongside with a natural, “imperfect” intonation, a fit between a conveyed emotion and content of the utterance/context is essential for making a voice human-like. Further research is needed to test different artificial voice styles, manipulating the intonation, the sound, their emotional content and implied personality of the speaker.

## Data Availability Statement

The datasets and stimuli of this study are available on request to the corresponding author.

## Ethics Statement

This study involving human participants was reviewed and approved by the Ethics Committee at the University of Potsdam, Germany. The participants provided their written informed consent to participate in this study.

## Author Contributions

KK and YZ contributed to the conception and design of the study. KK conceived the stimuli, programmed the survey, conducted the study, performed the analysis, and wrote the first draft of the manuscript. YZ and MF contributed to the analysis. KK, YZ, and MF wrote, discussed, and revised several drafts before approving the final version. All authors contributed to the article and approved the submitted version.

## Conflict of Interest

The authors declare that the research was conducted in the absence of any commercial or financial relationships that could be construed as a potential conflict of interest.

## References

[B1] Antona M. Ioannidi D. Foukarakis M. Gerlowska J. Rejdak K. Abdelnour C. (2019). My robot is happy today: how older people with mild cognitive impairments understand assistive robots' affective output, in Proceedings of the 12th ACM International Conference on Pervasive Technologies Related to Assistive Environments (Rhodes), 416–424. 10.1145/3316782.3322777

[B2] Aylett M. P. Sutton S. J. Vazquez-Alvarez Y. (2019). The right kind of unnatural: designing a robot voice, in Proceedings of the 1st International Conference on Conversational User Interfaces (Dublin), 1–2. 10.1145/3342775.3342806

[B3] Aylett M. P. Vazquez-Alvarez Y. Butkute S. (2020). Creating robot personality: effects of mixing speech and semantic free utterances, in Companion of the 2020 ACM/IEEE International Conference on Human-Robot Interaction (Cambridge), 110–112. 10.1145/3371382.3378330

[B4] Aylett M. P. Vinciarelli A. Wester M. (2017). Speech synthesis for the generation of artificial personality. IEEE Trans. Affect. Comput. 11, 361–372. 10.1109/TAFFC.2017.2763134

[B5] Baird A. Parada-Cabaleiro E. Hantke S. Burkhardt F. Cummins N. Schuller B. W. (2018). The perception and analysis of the likeability and human likeness of synthesized speech, in Interspeech (Hyderabad), 2863–2867. 10.21437/Interspeech.2018-1093

[B6] Bartneck C. Kanda T. Ishiguro H. Hagita N. (2007). Is the uncanny valley an uncanny cliff? in RO-MAN 2007-The 16th IEEE International Symposium on Robot and Human Interactive Communication (Jeju), 368–373. 10.1109/ROMAN.2007.4415111

[B7] Belpaeme T. Vogt P. Van den Berghe R. Bergmann K. Göksun T. De Haas M.. (2018). Guidelines for designing social robots as second language tutors. Int. J. Soc. Robot. 10, 325–341. 10.1007/s12369-018-0467-6 30996752 PMC6438435

[B8] Bendel O. (2020). Die Maschine an meiner Seite: Philosophische Betrachtungen zur Mensch-Roboter-Kollaboration, in Mensch-Roboter-Kollaboration, ed Buxbaum H.-J. (Wiesbaden: Springer Gabler). 1–14. 10.1007/978-3-658-28307-0_1

[B9] Bernier E. P. Scassellati B. (2010). The similarity-attraction effect in human-robot interaction, in 2010 IEEE 9th International Conference on Development and Learning (Ann Arbor, MI), 286–290. 10.1109/DEVLRN.2010.5578828

[B10] Berscheid E. Reis H. T. (1998). Attraction and close relationships, in Handbook of Social Psychology, Vol. 1-2, eds Fiske S. Gilbert D. Lindzey G. Aronson E. (New York, NY: Oxford University Press), 193–281.

[B11] Birkholz P. Martin L. Xu Y. Scherbaum S. Neuschaefer-Rube C. (2017). Manipulation of the prosodic features of vocal tract length, nasality, and articulatory precision using articulatory synthesis. Comput. Speech Lang. 41, 116–127. 10.1016/j.csl.2016.06.004

[B12] Bombelli G. Soler L. R. Waasaf M. S. (2013). The language of evaluation: paralinguistic features as a phonological domain for appraisal. Doc. Estud. Lingüíst. Teór. Apl. 29, 267–280. 10.1590/S0102-44502013000200004

[B13] Braun M. Mainz A. Chadowitz R. Pfleging B. Alt F. (2019). At your service: designing voice assistant personalities to improve automotive user interfaces, in Proceedings of the 2019 CHI Conference on Human Factors in Computing Systems (Glasgow), 1–11. 10.1145/3290605.3300270

[B14] Breazeal C. (2017). Social robots: from research to commercialization, in Proceedings of the 2017 ACM/IEEE International Conference on Human-Robot Interaction (Vienna), 1–1. 10.1145/2909824.3020258

[B15] Breazeal C. L. (2002). Designing Sociable Robots. Cambridge, MA: MIT Press 10.1007/0-306-47373-9_18

[B16] Broadbent E. (2017). Interactions with robots: the truths we reveal about ourselves. Ann. Rev. Psychol. 68, 627–652. 10.1146/annurev-psych-010416-043958 27648986

[B17] Bryman A. Burgess R. G. (1994). Development in qualitative data analysis: an introduction, in Analyzing Qualitative Data, eds Bryman A. Burgess R. G. (London: Routledge).

[B18] Bucholz M. Hall K. (2016). Embodied sociolinguistics, in Sociolinguistics: Theoretical Debates, ed Coupland N. (Cambridge: Cambridge University Press), 173–197. 10.1017/CBO9781107449787.009

[B19] Burgoon J. K. (1978). A communication model of personal space violations: explication and an initial test. Hum. Commun. Res. 4, 129–142. 10.1111/j.1468-2958.1978.tb00603.x

[B20] Burgoon J. K. (2015). Expectancy violations theory, in The International Encyclopedia of Interpersonal Communication, eds Berger C. R. Roloff M. E. Wilson S. R. Dillard J. P. Caughlin J. Solomon D. (Hoboken, NJ: John Wiley & Sons). 10.1002/9781118540190.wbeic102

[B21] Burgoon J. K. Bonito J. A. Lowry P. B. Humpherys S. L. Moody G. D. Gaskin J. E. (2016). Application of expectancy violations theory to communication with and judgments about embodied agents during a decision-making task. Int. J. Hum. Comput. Stud. 91, 24–36. 10.1016/j.ijhcs.2016.02.002

[B22] Burleigh T. J. Schoenherr J. R. (2015). A reappraisal of the uncanny valley: categorical perception or frequency-based sensitization? Front. Psychol. 5:1488 10.3389/fpsyg.2014.01488 25653623 PMC4300869

[B23] Cabral J. P. Cowan B. R. Zibrek K. McDonnell R. (2017). The influence of synthetic voice on the evaluation of a virtual character, in INTERSPEECH (Stockholm), 229–233. 10.21437/Interspeech.2017-325

[B24] Castro-González Á. Admoni H. Scassellati B. (2016). Effects of form and motion on judgments of social robots? animacy, likability, trustworthiness, and unpleasantness. Int. J. Hum. Comput. Stud. 90, 27–38. 10.1016/j.ijhcs.2016.02.004

[B25] Chang R. C. S. Lu H. P. Yang P. (2018). Stereotypes or golden rules*?* Exploring likable voice traits of social robots as active aging companions for tech-savvy baby boomers in Taiwan. Comput. Hum. Behav. 84, 194–210. 10.1016/j.chb.2018.02.025

[B26] Coursey K. Pirzchalski S. McMullen M. Lindroth G. Furuushi Y. (2019). Living with harmony: a personal companion system by Realbotix™, in AI Love You, eds Zhou Y. Fischer M. H. (Cham: Springer), 77–95. 10.1007/978-3-030-19734-6_4

[B27] Craig S. D. Schroeder N. L. (2017). Reconsidering the voice effect when learning from a virtual human. Comput. Educ. 114, 193–205. 10.1016/j.compedu.2017.07.003

[B28] Danaher J. McArthur N. (eds.). (2017). Robot Sex: Social And Ethical Implications. Cambridge: MIT Press 10.7551/mitpress/9780262036689.001.0001

[B29] Dennett D. C. (1971). Intentional systems. J. Philos. 68, 87–106. 10.2307/2025382

[B30] Dey I. (2003). Qualitative Data Analysis: A User Friendly Guide for Social Scientists. Routledge.

[B31] Elkins A. C. Derrick D. C. (2013). The sound of trust: voice as a measurement of trust during interactions with embodied conversational agents. Group Decis. Negot. 22, 897–913. 10.1007/s10726-012-9339-x

[B32] Gong L. Lai J. (2001). Shall we mix synthetic speech and human speech? impact on users' performance, perception, and attitude, in Proceedings of the SIGCHI Conference on Human Factors in Computing Systems (Seattle, WA), 158–165. 10.1145/365024.365090

[B33] Goy H. Kathleen Pichora-Fuller M. van Lieshout P. (2016). Effects of age on speech and voice quality ratings. J. Acoust. Soc. Am. 139, 1648–1659. 10.1121/1.4945094 27106312

[B34] Graziano W. G. Bruce J. Sheese B. E. Tobin R. M. (2007). Attraction, personality, and prejudice: liking none of the people most of the time. J. Pers. Soc. Psychol. 93:565. 10.1037/0022-3514.93.4.565 17892332

[B35] Greenwald A. G. McGhee D. E. Schwartz J. L. (1998). Measuring individual differences in implicit cognition: the implicit association test. J. Pers. Soc. Psychol. 74:1464. 10.1037/0022-3514.74.6.1464 9654756

[B36] Hannuschke M. Gollwitzer M. Geukes K. Nestler S. Back M. (2020). Neuroticism and interpersonal perception: Evidence for positive, but not negative, biases. J. Pers. 88, 217–236. 10.1111/jopy.12480 30985001

[B37] Harris R. A. (2004). Voice Interaction Design: Crafting the New Conversational Speech Systems. Elsevier.

[B38] Hinterleitner F. (2017). Quality of Synthetic Speech: Perceptual Dimensions, Influencing Factors, and Instrumental Assessment. New York, NY: Springer 10.1007/978-981-10-3734-4_5

[B39] Hirai S. Imono M. Tsuchiya S Watabe H. (2015). Speech synthesis method with emotion considering acoustic features of Voice, in 14th Information Science and Technology Forum, Vol. 2, 289–290.

[B40] Hodari Z. Watts O. King S. (2019). Using Generative Modelling to Produce Varied Intonation for Speech Synthesis. arXiv [Preprint]. Available online at: https://arxiv.org/abs/1906.04233 (accessed July 15, 2020). 10.21437/SSW.2019-43

[B41] Jaiswal J. Chaubey A. Bhimavarapu S. K. R. Kashyap S. Kumar P. Raman B. (2019). A generative adversarial network based ensemble technique for automatic evaluation of machine synthesized speech, in Asian Conference on Pattern Recognition (Cham: Springer), 580–593. 10.1007/978-3-030-41299-9_45

[B42] Kätsyri J. de Gelder B. Takala T. (2019). Virtual faces evoke only a weak uncanny valley effect: an empirical investigation with controlled virtual face images. Perception 48, 968–991. 10.1177/0301006619869134 31474183

[B43] Kayte S. N. Mundada M. Gaikwad S. Gawali B. (2016). Performance evaluation of speech synthesis techniques for English language, in Proceedings of the International Congress on Information and Communication Technology, eds Satapathy S. C. Bhatt Y. C. Joshi A. Mishra D. K. (New York, NY), 253–262. 10.1007/978-981-10-0755-2_27

[B44] Kenny D. A. (1994). Interpersonal Perception: A Social Relations Analysis. New York, NY: Guilford Press.

[B45] Kim S. Y. Schmitt B. H. Thalmann N. M. (2019). Eliza in the uncanny valley: anthropomorphizing consumer robots increases their perceived warmth but decreases liking. Mark. Lett. 30, 1–12. 10.1007/s11002-019-09485-9

[B46] Kory-Westlund J. M. Jeong S. Park H. W. Ronfard S. Adhikari A. Harris P. L.. (2017). Flat vs. expressive storytelling: young children's learning and retention of a social robot's narrative. Front. Human Neurosci. 11:295. 10.3389/fnhum.2017.00295 28638330 PMC5461341

[B47] Kory-Westlund J. M. Martinez M. Archie M. Das M. Breazeal C. (2016). Effects of framing a robot as a social agent or as a machine on children's social behavior, in 2016 25th IEEE International Symposium on Robot and Human Interactive Communication (RO-MAN) (New York, NY), 688–693. 10.1109/ROMAN.2016.7745193

[B48] Kraus M. Kraus J. Baumann M. Minker W. (2018). Effects of gender stereotypes on trust and likability in spoken human-robot interaction, in Proceedings of the Eleventh International Conference on Language Resources and Evaluation (LREC 2018) (Miyazaki). Available https://www.aclweb.org/anthology/L18-1018

[B49] Kuratate T. Ayers K. Kim J. Riley M. Burnham D. (2009). Are virtual humans uncanny?: varying speech, appearance and motion to better understand the acceptability of synthetic humans. AVSP. 65–69.

[B50] Leiner D. J. (2018). SoSci Survey (Version 3.2.05-i) [Computer Software]. Available online at: http://www.soscisurvey.com (accessed November 17, 2020).

[B51] Liscombe J. Venditti J. Hirschberg J. (2003). Classifying subject ratings of emotional speech using acoustic features, in Proceedings of Interspeech'2003 - Eurospeech.

[B52] MacDorman K. F. (2006). Subjective ratings of robot video clips for human likeness, familiarity, and eeriness: an exploration of the uncanny valley, in ICCS/CogSci-2006 Long Symposium: Toward Social Mechanisms of Android Science (Vancouver, BC), 26–29.

[B53] MacDorman K. F. Entezari S. O. (2015). Individual differences predict sensitivity to the uncanny valley. Interact. Stud. 16, 141–172. 10.1075/is.16.2.01mac

[B54] Marchesi S. Ghiglino D. Ciardo F. Perez-Osorio J. Baykara E. Wykowska A. (2019). Do we adopt the intentional stance toward humanoid robots? Front. Psychol. 10:450. 10.3389/fpsyg.2019.00450 30930808 PMC6428708

[B55] Massaro D. W. (1987). Speech Perception by Ear and Eye: *A Paradigm for Psychological Inquiry* Hillsdale, NJ: Erlbaum.

[B56] Massaro D. W. Simpson J. A. (2014). Speech Perception by Ear and Eye: A Paradigm for Psychological Inquiry. New York, NY: Psychology Press. 10.4324/9781315808253

[B57] Mayring P. Fenzl T. (2014). Qualitative inhaltsanalyse, in Handbuch Methoden der Empirischen Sozialforschung, eds Baur N. Blasius J. (Wiesbaden: Springer VS).

[B58] McCrae R. R. Costa P. T Jr. (1997). Personality trait structure as a human universal. Am. Psychol. 52:509. 10.1037/0003-066X.52.5.509 9145021

[B59] McCrae R. R. Terracciano A. (2005). Universal features of personality traits from the observer's perspective: data from 50 cultures. J. Pers. Soc. Psychol. 88:547. 10.1037/0022-3514.88.3.547 15740445

[B60] McGinn C. Torre I. (2019). Can you tell the robot by the voice? an exploratory study on the role of voice in the perception of robots, in 14th ACM/IEEE International Conference on Human-Robot Interaction (HRI) (Daegu), 211–221. 10.1109/HRI.2019.8673305

[B61] Mendelson J. Aylett M. P. (2017). Beyond the listening test: an interactive approach to TTS evaluation, in INTERSPEECH (Stockholm), 249–253. 10.21437/Interspeech.2017-1438

[B62] Mitchell W. J. Szerszen K. A. Lu A. S. Schermerhorn P. W. Scheutz M. MacDorman K. F. (2011). A mismatch in the human realism of face and voice produces an uncanny valley. I-Perception 2, 10–12. 10.1068/i0415 23145223 PMC3485769

[B63] Mori M. (1970). The uncanny valley. Energy 7, 33–35.

[B64] Mullennix J. W. Stern S. E. Wilson S. J. Dyson C. (2003). Social perception of male and female computer synthesized speech. Comput. Hum. Behav. 19, 407–424. 10.1016/S0747-5632(02)00081-X

[B65] Nass C. Lee K. M. (2001). Does computer-synthesized speech manifest personality*?* Experimental tests of recognition, similarity-attraction, and consistency-attraction. J. Exp. Psychol. Appl. 7:171. 10.1037/1076-898X.7.3.171 11676096

[B66] Niculescu A. van Dijk B. Nijholt A. Li H. See S. L. (2013). Making social robots more attractive: the effects of voice pitch, humor, and empathy. Int. J. Soc. Robot. 5, 171–191. 10.1007/s12369-012-0171-x

[B67] Nomura T. Kanda T. Suzuki T. Kato K. (2008). Prediction of human behavior in human–robot interaction using psychological scales for anxiety and negative attitudes toward robots. IEEE Trans. Robot. 24, 442–451. 10.1109/TRO.2007.914004

[B68] Polkosky M. D. Lewis J. R. (2003). Expanding the MOS: development and psychometric evaluation of the MOS-R and MOS-X. Int. J. Speech Technol. 6, 161–182. 10.1023/A:1022390615396

[B69] Pütten A. M. R. Krämer N. C. Maderwald S. Brand M. Grabenhorst F. (2019). Neural mechanisms for accepting and rejecting artificial social partners in the uncanny valley. J. Neurosci. 39, 6555–6570. 10.1523/JNEUROSCI.2956-18.2019 31263064 PMC6697392

[B70] Rammstedt B. John O. P. (2007). Measuring personality in one minute or less: a 10-item short version of the big five inventory in English and German. J. Res. Pers. 41, 203–212. 10.1016/j.jrp.2006.02.001

[B71] Rodero E. (2017). Effectiveness, attention, and recall of human and artificial voices in an advertising story. Prosody influence and functions of voices. Comput. Hum. Behav. 77, 336–346. 10.1016/j.chb.2017.08.044

[B72] Romportl J. (2014). Speech synthesis and uncanny valley, in Text, Speech, and Dialogue, eds Sojka P. Horák A. Kopeček I. Pala K. (Cham: Springer International Publishing), 595–602. 10.1007/978-3-319-10816-2_72

[B73] Salza P. L. Foti E. Nebbia L. Oreglia M. (1996). MOS and pair comparison combined methods for quality evaluation of text-to-speech systems. Acta Acust. United Acust. 82, 650–656.

[B74] Schmidt-Nielsen A. (1995). A test of speaker recognition using human listeners, in Proceedings. IEEE Workshop on Speech Coding for Telecommunications (Annapolis, MD: IEEE), 5–16. 10.1109/SCFT.1995.658104

[B75] Schuller B. Steidl S. Batliner A. Burkhardt F. Devillers L. MüLler C. (2013). Paralinguistics in speech and language—State-of-the-art and the challenge. Comput. Speech Lang. 27, 4–39. 10.1016/j.csl.2012.02.005

[B76] Scott K. M. Ashby S. Hanna J. (2020). “Human, All Too Human”: NOAA weather radio and the emotional impact of synthetic voices, in Proceedings of the 2020 CHI Conference on Human Factors in Computing Systems (Honolulu, HI), 1–9. 10.1145/3313831.3376338

[B77] Seymour M. Riemer K. Kay J. (2017). Interactive realistic digital avatars—revisiting the uncanny valley, in Hawaii International Conference on System Sciences 2017 (HICSS-50) (Honolulu, HI). Available online at: https://aisel.aisnet.org/hicss-50/cl/hci/4

[B78] Sims V. K. Chin M. G. Lum H. C. Upham-Ellis L. Ballion T. Lagattuta N. C. (2009). Robots' auditory cues are subject to anthropomorphism, in Proceedings of the Human Factors and Ergonomics Society Annual Meeting, Vol. 53 (Los Angeles, CA), 1418–1421. 10.1177/154193120905301853

[B79] Stern S. E. Mullennix J. W. Dyson C. L. Wilson S. J. (1999). The persuasiveness of synthetic speech versus human speech. Hum. Factors 41, 588–595. 10.1518/001872099779656680 10774129

[B80] Strait M. Vujovic L. Floerke V. Scheutz M. Urry H. (2015). Too much humanness for human-robot interaction: exposure to highly humanlike robots elicits aversive responding in observers, in Proceedings of the 33rd Annual ACM Conference on Human Factors in Computing Systems (Seoul), 3593–3602. 10.1145/2702123.2702415

[B81] Text to Speech Demo (n.d.) Available online at: https://text-to-speech-demo.ng.bluemix.net/ (accessed January 11, 2020).

[B82] Thepsoonthorn C. Ogawa K. I. Miyake Y. (2018). The relationship between robot's nonverbal behaviour and human's likability based on human's personality. Sci. Rep. 8, 1–11. 10.1038/s41598-018-25314-x 29849079 PMC5976716

[B83] Thomas D. R. (2003). A General Inductive Approach for Qualitative Data Analysis. Retrieved from: https://frankumstein.com/PDF/Psychology/Inductive%20Content%20Analysis.pdf

[B84] Torre I. Goslin J. White L. Zanatto D. (2018). Trust in artificial voices: A “congruency effect” of first impressions and behavioural experience, in Proceedings of the Technology, Mind, and Society (Washington, DC), 1–6. 10.1145/3183654.3183691

[B85] Tschöpe N. Reiser J. E. Oehl M. (2017). Exploring the uncanny valley effect in social robotics, in Proceedings of the Companion of the 2017 ACM/IEEE International Conference on Human-Robot Interaction (Vienna), 307–308. 10.1145/3029798.3038319

[B86] Tsiourti C. Weiss A. Wac K. Vincze M. (2019). Multimodal integration of emotional signals from voice, body, and context: effects of (in) congruence on emotion recognition and attitudes towards robots. Int. J. Soc. Robot. 11, 555–573. 10.1007/s12369-019-00524-z

[B87] Tyagi S. Nicolis M. Rohnke J. Drugman T. Lorenzo-Trueba J. (2019). Dynamic prosody generation for speech synthesis using linguistics-driven acoustic embedding selection. arXiv [Preprint] arXiv:1912.00955.

[B88] Uhrig S. Arndt S. Möller S. Voigt-Antons J.-N. (2017). Perceptual references for independent dimensions of speech quality as measured by electroencephalography. Qual. User Exp. 2:10 10.1007/s41233-017-0011-8

[B89] Vallee M. (2017). Technology, embodiment, and affect in voice sciences: the voice is an imaginary organ. Body Soc. 23, 83–105. 10.1177/1357034X17697366

[B90] Velner E. Boersma P. P. de Graaf M. M. (2020). Intonation in robot speech: does it work the same as with people? in Proceedings of the 2020 ACM/IEEE International Conference on Human-Robot Interaction (Cambridge), 569–578. 10.1145/3319502.3374801

[B91] Wagner P. Beskow J. Betz S. Edlund J. Gustafson J. Eje Henter G. (2019). Speech synthesis evaluation—state-of-the-art assessment and suggestion for a novel research program, in Proceedings of the 10th Speech Synthesis Workshop (SSW10) (Vienna). 10.21437/SSW.2019-19

[B92] Wasala K. Gomez R. Donovan J. Chamorro-Koc M. (2019). Emotion specific body movements: studying humans to augment robots' bodily expressions, in Proceedings of the 31st Australian Conference on Human-Computer-Interaction (Fremantle, WA), 503–507. 10.1145/3369457.3369542

[B93] Winquist L. A. Mohr C. D. Kenny D. A. (1998). The female positivity effect in the perception of others. J. Res. Pers. 32, 370–388. 10.1006/jrpe.1998.2221

[B94] Wood D. Harms P. Vazire S. (2010). Perceiver effects as projective tests: what your perceptions of others say about you. J. Pers. Soc. Psychol. 99, 174–190. 10.1037/a0019390 20565194

[B95] Yamamoto K. Takahashi K. Kishiro K. Sasaki S. Hayashi H. (2018). Analysis of emotional expression by visualization of the human and synthesized speech signal sets—A consideration of audio-visual advantage, in 2018 International Workshop on Advanced Image Technology (IWAIT) (Chiang Mai), 1–4. 10.1109/IWAIT.2018.8369809

[B96] Zhou Y. Fischer M. H. (2019). Intimate relationships with humanoid robots: exploring human sexuality in the twenty-first century, in AI Love You, eds Zhou Y. Fischer M. H. (Cham: Springer), 177–184. 10.1007/978-3-030-19734-6_10

[B97] Złotowski J. A. Sumioka H. Nishio S. Glas D. F. Bartneck C. Ishiguro H. (2018). Persistence of the uncanny valley, in Geminoid Studies: Science and Technologies for Humanlike Teleoperated Androids, eds Ishiguro H. Dalla Libera F. (Berlin: Springer), 163–187. 10.1007/978-981-10-8702-8_10

