# Supplementary material for: The Human Takes It All: Humanlike Synthesized Voices Are Perceived as Less Eerie and More Likable. Evidence From a Subjective Ratings Study
Source: Front Neurorobot. 2020 Dec 16;14:593732. doi: 10.3389/fnbot.2020.593732 (PMC7772241; doi:10.3389/fnbot.2020.593732)
Supplement: Supplementary file 1 [file Table_1.DOCX]

Demographic factors

Supplementary Table 1

*Results of linear mixed modeling with language, voice type, demographic factors as predictors and intelligibility as outcome*

|  | | | | | | | | | | | |
| --- | --- | --- | --- | --- | --- | --- | --- | --- | --- | --- | --- |
| Factor | | Estimate | | *SE* | | *df* | | *t* | | *p* | |
| Intercept |  | 5.257 |  | 0.334 |  | 90.142 |  | 15.718 |  | < .001 |  |
| **Language (1)** |  | **-0.801** |  | **0.327** |  | **87.108** |  | **-2.450** |  | **0.016** |  |
| **Voice (1)** |  | **1.635** |  | **0.257** |  | **101.118** |  | **6.374** |  | **< .001** |  |
| **Voice (2)** |  | **-0.686** |  | **0.214** |  | **102.312** |  | **-3.209** |  | **0.002** |  |
| Gender (1) |  | 0.401 |  | 0.327 |  | 87.108 |  | 1.226 |  | 0.223 |  |
| Age |  | -9.722e -4 |  | 0.011 |  | 87.108 |  | -0.090 |  | 0.928 |  |
| **Language (1) * Voice (1)** |  | **0.795** |  | **0.257** |  | **101.118** |  | **3.100** |  | **0.003** |  |
| Language (1) * Voice (2) |  | -0.010 |  | 0.214 |  | 102.312 |  | -0.049 |  | 0.961 |  |
| Language (1) * Gender (1) |  | 0.373 |  | 0.327 |  | 87.108 |  | 1.142 |  | 0.257 |  |
| Voice (1) * Gender (1) |  | -0.269 |  | 0.257 |  | 101.118 |  | -1.049 |  | 0.297 |  |
| Voice (2) * Gender (1) |  | 0.261 |  | 0.214 |  | 102.312 |  | 1.220 |  | 0.225 |  |
| Language (1) * Age |  | 0.018 |  | 0.011 |  | 87.108 |  | 1.687 |  | 0.095 |  |
| Voice (1) * Age |  | -0.008 |  | 0.008 |  | 101.118 |  | -0.934 |  | 0.352 |  |
| Voice (2) * Age |  | 6.616e -4 |  | 0.007 |  | 102.312 |  | 0.094 |  | 0.925 |  |
| Gender (1) * Age |  | -0.004 |  | 0.011 |  | 87.108 |  | -0.391 |  | 0.697 |  |
| Language (1) * Voice (1) * Gender (1) |  | -0.300 |  | 0.257 |  | 101.118 |  | -1.169 |  | 0.245 |  |
| Language (1) * Voice (2) * Gender (1) |  | 0.295 |  | 0.214 |  | 102.312 |  | 1.379 |  | 0.171 |  |
| Language (1) * Voice (1) * Age |  | -0.016 |  | 0.008 |  | 101.118 |  | -1.855 |  | 0.067 |  |
| Language (1) * Voice (2) * Age |  | 0.001 |  | 0.007 |  | 102.312 |  | 0.188 |  | 0.852 |  |
| Language (1) * Gender (1) * Age |  | -0.009 |  | 0.011 |  | 87.108 |  | -0.806 |  | 0.422 |  |
| Voice (1) * Gender (1) * Age |  | 0.004 |  | 0.008 |  | 101.118 |  | 0.499 |  | 0.619 |  |
| Voice (2) * Gender (1) * Age |  | -0.007 |  | 0.007 |  | 102.312 |  | -1.026 |  | 0.307 |  |
| Language (1) * Voice (1) * Gender (1) * Age |  | 0.008 |  | 0.008 |  | 101.118 |  | 0.970 |  | 0.335 |  |
| Language (1) * Voice (2) * Gender (1) * Age |  | -0.008 |  | 0.007 |  | 102.312 |  | -1.120 |  | 0.265 |  |
|  | | | | | | | | | | | |
| *Note.*  The intercept corresponds to the (unweighted) grand mean; for each factor with k levels, k - 1 parameters are estimated. Consequently, the estimates cannot be directly mapped to factor levels. Significant effects are highlighted in bold. | | | | | | | | | | | |

| Supplementary Table 2  *Results of linear mixed modeling with language, voice type, demographic factors as predictors and prosody as outcome* | | | | | | | | | | | |
| --- | --- | --- | --- | --- | --- | --- | --- | --- | --- | --- | --- |
| Factor | | Estimate | | *SE* | | *df* | | *t* | | *p* | |
| Intercept |  | 3.952 |  | 0.255 |  | 99.723 |  | 15.479 |  | < .001 |  |
| **Language (1)** |  | **-0.571** |  | **0.240** |  | **86.675** |  | **-2.381** |  | **0.019** |  |
| **Voice (1)** |  | **2.612** |  | **0.272** |  | **99.207** |  | **9.592** |  | **< .001** |  |
| **Voice (2)** |  | **-1.182** |  | **0.202** |  | **80.906** |  | **-5.855** |  | **< .001** |  |
| Gender (1) |  | 0.234 |  | 0.240 |  | 86.675 |  | 0.976 |  | 0.332 |  |
| Age |  | 0.006 |  | 0.008 |  | 86.655 |  | 0.749 |  | 0.456 |  |
| **Language (1) * Voice (1)** |  | **0.734** |  | **0.253** |  | **86.582** |  | **2.905** |  | **0.005** |  |
| **Language (1) * Voice (2)** |  | **-0.485** |  | **0.169** |  | **86.265** |  | **-2.862** |  | **0.005** |  |
| Language (1) * Gender (1) |  | 0.316 |  | 0.240 |  | 86.675 |  | 1.320 |  | 0.190 |  |
| Voice (1) * Gender (1) |  | -0.117 |  | 0.253 |  | 86.582 |  | -0.464 |  | 0.644 |  |
| **Voice (2) * Gender (1)** |  | **0.466** |  | **0.169** |  | **86.265** |  | **2.749** |  | **0.007** |  |
| Language (1) * Age |  | 0.010 |  | 0.008 |  | 86.655 |  | 1.329 |  | 0.187 |  |
| Voice (1) * Age |  | -0.014 |  | 0.008 |  | 86.508 |  | -1.627 |  | 0.107 |  |
| Voice (2) * Age |  | 0.005 |  | 0.006 |  | 86.225 |  | 0.961 |  | 0.339 |  |
| Gender (1) * Age |  | 3.734e -4 |  | 0.008 |  | 86.655 |  | 0.047 |  | 0.962 |  |
| Language (1) * Voice (1) * Gender (1) |  | -0.364 |  | 0.253 |  | 86.582 |  | -1.439 |  | 0.154 |  |
| **Language (1) * Voice (2) * Gender (1)** |  | **0.371** |  | **0.169** |  | **86.265** |  | **2.189** |  | **0.031** |  |
| Language (1) * Voice (1) * Age |  | -0.014 |  | 0.008 |  | 86.508 |  | -1.674 |  | 0.098 |  |
| **Language (1) * Voice (2) * Age** |  | **0.014** |  | **0.006** |  | **86.225** |  | **2.482** |  | **0.015** |  |
| Language (1) * Gender (1) * Age |  | -0.006 |  | 0.008 |  | 86.655 |  | -0.818 |  | 0.416 |  |
| Voice (1) * Gender (1) * Age |  | -0.002 |  | 0.008 |  | 86.508 |  | -0.244 |  | 0.808 |  |
| **Voice (2) * Gender (1) * Age** |  | **-0.013** |  | **0.006** |  | **86.225** |  | **-2.392** |  | **0.019** |  |
| Language (1) * Voice (1) * Gender (1) * Age |  | 0.010 |  | 0.008 |  | 86.508 |  | 1.250 |  | 0.215 |  |
| Language (1) * Voice (2) * Gender (1) * Age |  | -0.005 |  | 0.006 |  | 86.225 |  | -0.867 |  | 0.388 |  |
|  | | | | | | | | | | | |
| *Note.*  The intercept corresponds to the (unweighted) grand mean; for each factor with k levels, k - 1 parameters are estimated. Consequently, the estimates cannot be directly mapped to factor levels. Significant effects are highlighted in bold. | | | | | | | | | | | |

| Supplementary Table 3  *Results of linear mixed modeling with language, voice type, demographic factors as predictors and trustworthiness as outcome* | | | | | | | | | | | |
| --- | --- | --- | --- | --- | --- | --- | --- | --- | --- | --- | --- |
| Factor | | Estimate | | *SE* | | *df* | | *t* | | *p* | |
| Intercept |  | 3.924 |  | 0.319 |  | 81.289 |  | 12.294 |  | < .001 |  |
| Language (1) |  | -0.281 |  | 0.301 |  | 86.565 |  | -0.933 |  | 0.354 |  |
| **Voice (1)** |  | **2.241** |  | **0.188** |  | **1213.858** |  | **11.919** |  | **< .001** |  |
| **Voice (2)** |  | **-1.164** |  | **0.188** |  | **1213.858** |  | **-6.192** |  | **< .001** |  |
| Gender (1) |  | 0.400 |  | 0.301 |  | 86.565 |  | 1.329 |  | 0.187 |  |
| Age |  | -4.574e -4 |  | 0.010 |  | 86.565 |  | -0.046 |  | 0.963 |  |
| **Language (1) * Voice (1)** |  | **0.625** |  | **0.188** |  | **1213.858** |  | **3.323** |  | **< .001** |  |
| **Language (1) * Voice (2)** |  | **-0.476** |  | **0.188** |  | **1213.858** |  | **-2.531** |  | **0.011** |  |
| Language (1) * Gender (1) |  | 0.142 |  | 0.301 |  | 86.565 |  | 0.471 |  | 0.639 |  |
| Voice (1) * Gender (1) |  | -0.068 |  | 0.188 |  | 1213.858 |  | -0.360 |  | 0.719 |  |
| **Voice (2) * Gender (1)** |  | **0.372** |  | **0.188** |  | **1213.858** |  | **1.977** |  | **0.048** |  |
| Language (1) * Age |  | 7.694e -4 |  | 0.010 |  | 86.565 |  | 0.078 |  | 0.938 |  |
| Voice (1) * Age |  | -0.009 |  | 0.006 |  | 1213.858 |  | -1.528 |  | 0.127 |  |
| Voice (2) * Age |  | 0.006 |  | 0.006 |  | 1213.858 |  | 0.922 |  | 0.357 |  |
| Gender (1) * Age |  | -0.006 |  | 0.010 |  | 86.565 |  | -0.604 |  | 0.548 |  |
| **Language (1) * Voice (1) * Gender (1)** |  | **-0.552** |  | **0.188** |  | **1213.858** |  | **-2.934** |  | **0.003** |  |
| **Language (1) * Voice (2) * Gender (1)** |  | **0.470** |  | **0.188** |  | **1213.858** |  | **2.499** |  | **0.013** |  |
| Language (1) * Voice (1) * Age |  | -0.010 |  | 0.006 |  | 1213.858 |  | -1.624 |  | 0.105 |  |
| Language (1) * Voice (2) * Age |  | 0.012 |  | 0.006 |  | 1213.858 |  | 1.914 |  | 0.056 |  |
| Language (1) * Gender (1) * Age |  | -0.002 |  | 0.010 |  | 86.565 |  | -0.230 |  | 0.819 |  |
| Voice (1) * Gender (1) * Age |  | -0.007 |  | 0.006 |  | 1213.858 |  | -1.206 |  | 0.228 |  |
| Voice (2) * Gender (1) * Age |  | -0.009 |  | 0.006 |  | 1213.858 |  | -1.444 |  | 0.149 |  |
| **Language (1) * Voice (1) * Gender (1) * Age** |  | **0.022** |  | **0.006** |  | **1213.858** |  | **3.619** |  | **< .001** |  |
| Language (1) * Voice (2) * Gender (1) * Age |  | -0.012 |  | 0.006 |  | 1213.858 |  | -1.876 |  | 0.061 |  |
|  | | | | | | | | | | | |
| *Note.*  The intercept corresponds to the (unweighted) grand mean; for each factor with k levels, k - 1 parameters are estimated. Consequently, the estimates cannot be directly mapped to factor levels. Significant effects are highlighted in bold. | | | | | | | | | | | |

| Supplementary Table 4  *Results of linear mixed modeling with language, voice type, demographic factors as predictors and confidence as outcome* | | | | | | | | | | | |
| --- | --- | --- | --- | --- | --- | --- | --- | --- | --- | --- | --- |
| Factor | | Estimate | | *SE* | | *df* | | *t* | | *p* | |
| Intercept |  | 4.581 |  | 0.314 |  | 77.410 |  | 14.598 |  | < .001 |  |
| Language (1) |  | -0.808 |  | 0.292 |  | 86.669 |  | -2.764 |  | 0.007 |  |
| **Voice (1)** |  | **2.069** |  | **0.284** |  | **87.001** |  | **7.279** |  | **< .001** |  |
| **Voice (2)** |  | **-1.056** |  | **0.209** |  | **87.001** |  | **-5.050** |  | **< .001** |  |
| Gender (1) |  | 0.518 |  | 0.292 |  | 86.669 |  | 1.772 |  | 0.080 |  |
| Age |  | -0.017 |  | 0.010 |  | 86.669 |  | -1.720 |  | 0.089 |  |
| **Language (1) * Voice (1)** |  | **0.626** |  | **0.284** |  | **87.001** |  | **2.204** |  | **0.030** |  |
| Language (1) * Voice (2) |  | -0.386 |  | 0.209 |  | 87.001 |  | -1.847 |  | 0.068 |  |
| Language (1) * Gender (1) |  | 0.229 |  | 0.292 |  | 86.669 |  | 0.785 |  | 0.435 |  |
| Voice (1) * Gender (1) |  | -0.303 |  | 0.284 |  | 87.001 |  | -1.066 |  | 0.289 |  |
| Voice (2) * Gender (1) |  | 0.383 |  | 0.209 |  | 87.001 |  | 1.833 |  | 0.070 |  |
| **Language (1) * Age** |  | **0.021** |  | **0.010** |  | **86.669** |  | **2.222** |  | **0.029** |  |
| Voice (1) * Age |  | 0.001 |  | 0.009 |  | 87.001 |  | 0.141 |  | 0.888 |  |
| Voice (2) * Age |  | 0.002 |  | 0.007 |  | 87.001 |  | 0.245 |  | 0.807 |  |
| Gender (1) * Age |  | -0.009 |  | 0.010 |  | 86.669 |  | -0.976 |  | 0.332 |  |
| Language (1) * Voice (1) * Gender (1) |  | -0.400 |  | 0.284 |  | 87.001 |  | -1.408 |  | 0.163 |  |
| **Language (1) * Voice (2) * Gender (1)** |  | **0.449** |  | **0.209** |  | **87.001** |  | **2.145** |  | **0.035** |  |
| Language (1) * Voice (1) * Age |  | -0.015 |  | 0.009 |  | 87.001 |  | -1.556 |  | 0.123 |  |
| Language (1) * Voice (2) * Age |  | 0.012 |  | 0.007 |  | 87.001 |  | 1.792 |  | 0.077 |  |
| Language (1) * Gender (1) * Age |  | -0.004 |  | 0.010 |  | 86.669 |  | -0.398 |  | 0.692 |  |
| Voice (1) * Gender (1) * Age |  | 4.694e -4 |  | 0.009 |  | 87.001 |  | 0.050 |  | 0.960 |  |
| Voice (2) * Gender (1) * Age |  | -0.009 |  | 0.007 |  | 87.001 |  | -1.245 |  | 0.216 |  |
| Language (1) * Voice (1) * Gender (1) * Age |  | 0.016 |  | 0.009 |  | 87.001 |  | 1.733 |  | 0.087 |  |
| Language (1) * Voice (2) * Gender (1) * Age |  | -0.011 |  | 0.007 |  | 87.001 |  | -1.572 |  | 0.119 |  |
|  | | | | | | | | | | | |
| *Note.*  The intercept corresponds to the (unweighted) grand mean; for each factor with k levels, k - 1 parameters are estimated. Consequently, the estimates cannot be directly mapped to factor levels. Significant effects are highlighted in bold. | | | | | | | | | | | |

| Supplementary Table 5  *Results of linear mixed modeling with language, voice type, demographic factors as predictors and enthusiasm as outcome* | | | | | | | | | | | |
| --- | --- | --- | --- | --- | --- | --- | --- | --- | --- | --- | --- |
| Factor | | Estimate | | *SE* | | *df* | | *t* | | *p* | |
| Intercept |  | 4.274 |  | 0.342 |  | 82.078 |  | 12.507 |  | < .001 |  |
| Language (1) |  | -0.419 |  | 0.322 |  | 86.787 |  | -1.302 |  | 0.196 |  |
| **Voice (1)** |  | **2.463** |  | **0.198** |  | **262.172** |  | **12.465** |  | **< .001** |  |
| **Voice (2)** |  | **-1.485** |  | **0.207** |  | **113.676** |  | **-7.173** |  | **< .001** |  |
| Gender (1) |  | 0.317 |  | 0.322 |  | 86.787 |  | 0.984 |  | 0.328 |  |
| Age |  | -0.013 |  | 0.011 |  | 86.787 |  | -1.260 |  | 0.211 |  |
| Language (1) * Voice (1) |  | 0.250 |  | 0.191 |  | 1203.792 |  | 1.307 |  | 0.191 |  |
| Language (1) * Voice (2) |  | -0.260 |  | 0.191 |  | 1203.793 |  | -1.361 |  | 0.174 |  |
| Language (1) * Gender (1) |  | 0.304 |  | 0.322 |  | 86.787 |  | 0.943 |  | 0.348 |  |
| Voice (1) * Gender (1) |  | -0.180 |  | 0.191 |  | 1203.793 |  | -0.941 |  | 0.347 |  |
| Voice (2) * Gender (1) |  | 0.259 |  | 0.191 |  | 1203.793 |  | 1.352 |  | 0.177 |  |
| Language (1) * Age |  | 0.016 |  | 0.011 |  | 86.787 |  | 1.486 |  | 0.141 |  |
| **Voice (1) * Age** |  | **-0.016** |  | **0.006** |  | **1203.793** |  | **-2.567** |  | **0.010** |  |
| Voice (2) * Age |  | 0.012 |  | 0.006 |  | 1203.793 |  | 1.926 |  | 0.054 |  |
| Gender (1) * Age |  | -0.004 |  | 0.011 |  | 86.787 |  | -0.395 |  | 0.694 |  |
| Language (1) * Voice (1) * Gender (1) |  | -0.267 |  | 0.191 |  | 1203.793 |  | -1.397 |  | 0.163 |  |
| **Language (1) * Voice (2) * Gender (1)** |  | **0.437** |  | **0.191** |  | **1203.793** |  | **2.284** |  | **0.023** |  |
| Language (1) * Voice (1) * Age |  | -3.823e -4 |  | 0.006 |  | 1203.792 |  | -0.061 |  | 0.952 |  |
| Language (1) * Voice (2) * Age |  | 0.006 |  | 0.006 |  | 1203.793 |  | 0.971 |  | 0.332 |  |
| Language (1) * Gender (1) * Age |  | -0.010 |  | 0.011 |  | 86.787 |  | -0.943 |  | 0.348 |  |
| Voice (1) * Gender (1) * Age |  | 0.002 |  | 0.006 |  | 1203.793 |  | 0.288 |  | 0.773 |  |
| Voice (2) * Gender (1) * Age |  | -0.009 |  | 0.006 |  | 1203.793 |  | -1.351 |  | 0.177 |  |
| Language (1) * Voice (1) * Gender (1) * Age |  | 0.012 |  | 0.006 |  | 1203.793 |  | 1.958 |  | 0.050 |  |
| Language (1) * Voice (2) * Gender (1) * Age |  | -0.012 |  | 0.006 |  | 1203.793 |  | -1.832 |  | 0.067 |  |
|  | | | | | | | | | | | |
| *Note.*  The intercept corresponds to the (unweighted) grand mean; for each factor with k levels, k - 1 parameters are estimated. Consequently, the estimates cannot be directly mapped to factor levels. Significant effects are highlighted in bold. | | | | | | | | | | | |

| Supplementary Table 6  *Results of linear mixed modeling with language, voice type, demographic factors as predictors and pleasantness as outcome* | | | | | | | | | | | |
| --- | --- | --- | --- | --- | --- | --- | --- | --- | --- | --- | --- |
| Factor | | Estimate | | *SE* | | *df* | | *t* | | *p* | |
| Intercept |  | 4.023 |  | 0.328 |  | 84.497 |  | 12.283 |  | < .001 |  |
| Language (1) |  | -0.547 |  | 0.311 |  | 86.655 |  | -1.762 |  | 0.082 |  |
| **Voice (1)** |  | **2.278** |  | **0.285** |  | **87.003** |  | **8.000** |  | **< .001** |  |
| **Voice (2)** |  | **-1.170** |  | **0.234** |  | **87.007** |  | **-4.991** |  | **< .001** |  |
| Gender (1) |  | 0.255 |  | 0.311 |  | 86.655 |  | 0.820 |  | 0.414 |  |
| Age |  | -0.003 |  | 0.010 |  | 86.655 |  | -0.341 |  | 0.734 |  |
| Language (1) * Voice (1) |  | 0.383 |  | 0.285 |  | 87.003 |  | 1.345 |  | 0.182 |  |
| Language (1) * Voice (2) |  | -0.302 |  | 0.234 |  | 87.007 |  | -1.290 |  | 0.201 |  |
| Language (1) * Gender (1) |  | 0.117 |  | 0.311 |  | 86.655 |  | 0.377 |  | 0.707 |  |
| Voice (1) * Gender (1) |  | 0.072 |  | 0.285 |  | 87.003 |  | 0.254 |  | 0.800 |  |
| Voice (2) * Gender (1) |  | 0.315 |  | 0.234 |  | 87.007 |  | 1.344 |  | 0.182 |  |
| Language (1) * Age |  | 0.010 |  | 0.010 |  | 86.655 |  | 1.024 |  | 0.308 |  |
| Voice (1) * Age |  | -0.017 |  | 0.009 |  | 87.003 |  | -1.793 |  | 0.076 |  |
| Voice (2) * Age |  | 0.007 |  | 0.008 |  | 87.007 |  | 0.930 |  | 0.355 |  |
| Gender (1) * Age |  | -0.002 |  | 0.010 |  | 86.655 |  | -0.186 |  | 0.853 |  |
| Language (1) * Voice (1) * Gender (1) |  | -0.517 |  | 0.285 |  | 87.003 |  | -1.817 |  | 0.073 |  |
| Language (1) * Voice (2) * Gender (1) |  | 0.440 |  | 0.234 |  | 87.007 |  | 1.877 |  | 0.064 |  |
| Language (1) * Voice (1) * Age |  | -0.004 |  | 0.009 |  | 87.003 |  | -0.418 |  | 0.677 |  |
| Language (1) * Voice (2) * Age |  | 0.008 |  | 0.008 |  | 87.007 |  | 0.976 |  | 0.332 |  |
| Language (1) * Gender (1) * Age |  | -0.002 |  | 0.010 |  | 86.655 |  | -0.159 |  | 0.874 |  |
| Voice (1) * Gender (1) * Age |  | -0.011 |  | 0.009 |  | 87.003 |  | -1.158 |  | 0.250 |  |
| Voice (2) * Gender (1) * Age |  | -0.009 |  | 0.008 |  | 87.007 |  | -1.204 |  | 0.232 |  |
| **Language (1) * Voice (1) * Gender (1) * Age** |  | **0.020** |  | **0.009** |  | **87.003** |  | **2.176** |  | **0.032** |  |
| Language (1) * Voice (2) * Gender (1) * Age |  | -0.011 |  | 0.008 |  | 87.007 |  | -1.385 |  | 0.170 |  |
|  | | | | | | | | | | | |
| *Note.*  The intercept corresponds to the (unweighted) grand mean; for each factor with k levels, k - 1 parameters are estimated. Consequently, the estimates cannot be directly mapped to factor levels. Significant effects are highlighted in bold. | | | | | | | | | | | |

| Supplementary Table 7  *Results of linear mixed modeling with language, voice type, demographic factors as predictors and naturalness as outcome* | | | | | | | | | | | |
| --- | --- | --- | --- | --- | --- | --- | --- | --- | --- | --- | --- |
| Factor | | Estimate | | *SE* | | *df* | | *t* | | *p* | |
| Intercept |  | 3.709 |  | 0.268 |  | 81.961 |  | 13.850 |  | < .001 |  |
| **Language (1)** |  | **-0.831** |  | **0.255** |  | **86.605** |  | **-3.265** |  | **0.002** |  |
| **Voice (1)** |  | **2.942** |  | **0.194** |  | **1212.801** |  | **15.195** |  | **< .001** |  |
| **Voice (2)** |  | **-1.379** |  | **0.194** |  | **1212.801** |  | **-7.122** |  | **< .001** |  |
| Gender (1) |  | 0.479 |  | 0.255 |  | 86.605 |  | 1.880 |  | 0.063 |  |
| Age |  | -0.002 |  | 0.008 |  | 86.610 |  | -0.284 |  | 0.777 |  |
| **Language (1) * Voice (1)** |  | **0.798** |  | **0.194** |  | **1212.801** |  | **4.122** |  | **< .001** |  |
| **Language (1) * Voice (2)** |  | **-0.553** |  | **0.194** |  | **1212.801** |  | **-2.855** |  | **0.004** |  |
| Language (1) * Gender (1) |  | 0.105 |  | 0.255 |  | 86.605 |  | 0.413 |  | 0.680 |  |
| Voice (1) * Gender (1) |  | -0.287 |  | 0.194 |  | 1212.801 |  | -1.482 |  | 0.139 |  |
| **Voice (2) * Gender (1)** |  | **0.557** |  | **0.194** |  | **1212.801** |  | **2.878** |  | **0.004** |  |
| **Language (1) * Age** |  | **0.019** |  | **0.008** |  | **86.610** |  | **2.282** |  | **0.025** |  |
| Voice (1) * Age |  | -0.010 |  | 0.006 |  | 1212.804 |  | -1.585 |  | 0.113 |  |
| Voice (2) * Age |  | 0.006 |  | 0.006 |  | 1212.804 |  | 0.958 |  | 0.338 |  |
| Gender (1) * Age |  | -0.011 |  | 0.008 |  | 86.610 |  | -1.354 |  | 0.179 |  |
| Language (1) * Voice (1) * Gender (1) |  | -0.224 |  | 0.194 |  | 1212.801 |  | -1.159 |  | 0.247 |  |
| Language (1) * Voice (2) * Gender (1) |  | 0.295 |  | 0.194 |  | 1212.801 |  | 1.523 |  | 0.128 |  |
| **Language (1) * Voice (1) * Age** |  | **-0.016** |  | **0.006** |  | **1212.804** |  | **-2.486** |  | **0.013** |  |
| **Language (1) * Voice (2) * Age** |  | **0.014** |  | **0.006** |  | **1212.804** |  | **2.254** |  | **0.024** |  |
| Language (1) * Gender (1) * Age |  | 9.545e -5 |  | 0.008 |  | 86.610 |  | 0.011 |  | 0.991 |  |
| Voice (1) * Gender (1) * Age |  | 0.005 |  | 0.006 |  | 1212.804 |  | 0.815 |  | 0.415 |  |
| **Voice (2) * Gender (1) * Age** |  | **-0.017** |  | **0.006** |  | **1212.804** |  | **-2.697** |  | **0.007** |  |
| Language (1) * Voice (1) * Gender (1) * Age |  | 0.005 |  | 0.006 |  | 1212.804 |  | 0.814 |  | 0.416 |  |
| Language (1) * Voice (2) * Gender (1) * Age |  | -0.002 |  | 0.006 |  | 1212.804 |  | -0.293 |  | 0.769 |  |
|  | | | | | | | | | | | |
| *Note.*  The intercept corresponds to the (unweighted) grand mean; for each factor with k levels, k - 1 parameters are estimated. Consequently, the estimates cannot be directly mapped to factor levels. Significant effects are highlighted in bold. | | | | | | | | | | | |

| Supplementary Table 8  *Results of linear mixed modeling with language, voice type, demographic factors as predictors and human-likeness (voice) as outcome* | | | | | | | | | | | |
| --- | --- | --- | --- | --- | --- | --- | --- | --- | --- | --- | --- |
| Factor | | Estimate | | *SE* | | *df* | | *t* | | *p* | |
| Intercept |  | 3.673 |  | 0.268 |  | 81.037 |  | 13.729 |  | < .001 |  |
| **Language (1)** |  | **-1.098** |  | **0.254** |  | **86.590** |  | **-4.330** |  | **< .001** |  |
| **Voice (1)** |  | **3.190** |  | **0.193** |  | **1213.772** |  | **16.549** |  | **< .001** |  |
| **Voice (2)** |  | **-1.348** |  | **0.193** |  | **1213.772** |  | **-6.996** |  | **< .001** |  |
| **Gender (1)** |  | **0.580** |  | **0.254** |  | **86.590** |  | **2.289** |  | **0.025** |  |
| Age |  | 0.001 |  | 0.008 |  | 86.590 |  | 0.127 |  | 0.899 |  |
| **Language (1) * Voice (1)** |  | **0.945** |  | **0.193** |  | **1213.772** |  | **4.905** |  | **< .001** |  |
| **Language (1) * Voice (2)** |  | **-0.528** |  | **0.193** |  | **1213.772** |  | **-2.738** |  | **0.006** |  |
| Language (1) * Gender (1) |  | 0.253 |  | 0.254 |  | 86.590 |  | 0.999 |  | 0.321 |  |
| **Voice (1) * Gender (1)** |  | **-0.396** |  | **0.193** |  | **1213.772** |  | **-2.056** |  | **0.040** |  |
| **Voice (2) * Gender (1)** |  | **0.591** |  | **0.193** |  | **1213.772** |  | **3.065** |  | **0.002** |  |
| **Language (1) * Age** |  | **0.028** |  | **0.008** |  | **86.590** |  | **3.349** |  | **0.001** |  |
| **Voice (1) * Age** |  | **-0.014** |  | **0.006** |  | **1213.772** |  | **-2.183** |  | **0.029** |  |
| Voice (2) * Age |  | 0.003 |  | 0.006 |  | 1213.772 |  | 0.550 |  | 0.583 |  |
| Gender (1) * Age |  | -0.012 |  | 0.008 |  | 86.590 |  | -1.471 |  | 0.145 |  |
| Language (1) * Voice (1) * Gender (1) |  | -0.257 |  | 0.193 |  | 1213.772 |  | -1.333 |  | 0.183 |  |
| Language (1) * Voice (2) * Gender (1) |  | 0.260 |  | 0.193 |  | 1213.772 |  | 1.349 |  | 0.178 |  |
| **Language (1) * Voice (1) * Age** |  | **-0.021** |  | **0.006** |  | **1213.772** |  | **-3.266** |  | **0.001** |  |
| **Language (1) * Voice (2) * Age** |  | **0.013** |  | **0.006** |  | **1213.772** |  | **2.100** |  | **0.036** |  |
| Language (1) * Gender (1) * Age |  | -0.003 |  | 0.008 |  | 86.590 |  | -0.324 |  | 0.747 |  |
| Voice (1) * Gender (1) * Age |  | 0.006 |  | 0.006 |  | 1213.772 |  | 0.965 |  | 0.335 |  |
| **Voice (2) * Gender (1) * Age** |  | **-0.017** |  | **0.006** |  | **1213.772** |  | **-2.610** |  | **0.009** |  |
| Language (1) * Voice (1) * Gender (1) * Age |  | 0.005 |  | 0.006 |  | 1213.772 |  | 0.820 |  | 0.413 |  |
| Language (1) * Voice (2) * Gender (1) * Age |  | -9.283e -4 |  | 0.006 |  | 1213.772 |  | -0.146 |  | 0.884 |  |
|  | | | | | | | | | | | |
| *Note.*  The intercept corresponds to the (unweighted) grand mean; for each factor with k levels, k - 1 parameters are estimated. Consequently, the estimates cannot be directly mapped to factor levels. Significant effects are highlighted in bold. | | | | | | | | | | | |

| Supplementary Table 9  *Results of linear mixed modeling with language, voice type, demographic factors as predictors and likability as outcome* | | | | | | | | | | | |
| --- | --- | --- | --- | --- | --- | --- | --- | --- | --- | --- | --- |
| Factor | | Estimate | | *SE* | | *df* | | *t* | | *p* | |
| Intercept |  | 3.760 |  | 0.336 |  | 88.419 |  | 11.203 |  | < .001 |  |
| Language (1) |  | -0.280 |  | 0.324 |  | 86.754 |  | -0.863 |  | 0.390 |  |
| **Voice (1)** |  | **2.486** |  | **0.311** |  | **87.003** |  | **8.005** |  | **< .001** |  |
| **Voice (2)** |  | **-1.094** |  | **0.245** |  | **87.000** |  | **-4.460** |  | **< .001** |  |
| Gender (1) |  | 0.323 |  | 0.324 |  | 86.754 |  | 0.995 |  | 0.322 |  |
| Age |  | -0.002 |  | 0.011 |  | 86.754 |  | -0.178 |  | 0.859 |  |
| **Language (1) * Voice (1)** |  | **0.675** |  | **0.311** |  | **87.003** |  | **2.172** |  | **0.033** |  |
| Language (1) * Voice (2) |  | -0.328 |  | 0.245 |  | 87.000 |  | -1.339 |  | 0.184 |  |
| Language (1) * Gender (1) |  | -0.020 |  | 0.324 |  | 86.754 |  | -0.063 |  | 0.950 |  |
| Voice (1) * Gender (1) |  | -0.143 |  | 0.311 |  | 87.003 |  | -0.461 |  | 0.646 |  |
| **Voice (2) * Gender (1)** |  | **0.608** |  | **0.245** |  | **87.000** |  | **2.480** |  | **0.015** |  |
| Language (1) * Age |  | 8.616e -4 |  | 0.011 |  | 86.754 |  | 0.081 |  | 0.936 |  |
| Voice (1) * Age |  | -0.018 |  | 0.010 |  | 87.003 |  | -1.788 |  | 0.077 |  |
| Voice (2) * Age |  | 0.003 |  | 0.008 |  | 87.000 |  | 0.405 |  | 0.687 |  |
| Gender (1) * Age |  | -0.004 |  | 0.011 |  | 86.754 |  | -0.415 |  | 0.679 |  |
| Language (1) * Voice (1) * Gender (1) |  | -0.554 |  | 0.311 |  | 87.003 |  | -1.783 |  | 0.078 |  |
| **Language (1) * Voice (2) * Gender (1)** |  | **0.598** |  | **0.245** |  | **87.000** |  | **2.439** |  | **0.017** |  |
| Language (1) * Voice (1) * Age |  | -0.010 |  | 0.010 |  | 87.003 |  | -0.968 |  | 0.336 |  |
| Language (1) * Voice (2) * Age |  | 0.006 |  | 0.008 |  | 87.000 |  | 0.776 |  | 0.440 |  |
| Language (1) * Gender (1) * Age |  | 0.002 |  | 0.011 |  | 86.754 |  | 0.216 |  | 0.830 |  |
| Voice (1) * Gender (1) * Age |  | -0.007 |  | 0.010 |  | 87.003 |  | -0.662 |  | 0.510 |  |
| **Voice (2) * Gender (1) * Age** |  | **-0.017** |  | **0.008** |  | **87.000** |  | **-2.131** |  | **0.036** |  |
| Language (1) * Voice (1) * Gender (1) * Age |  | 0.020 |  | 0.010 |  | 87.003 |  | 1.913 |  | 0.059 |  |
| Language (1) * Voice (2) * Gender (1) * Age |  | -0.012 |  | 0.008 |  | 87.000 |  | -1.546 |  | 0.126 |  |
|  | | | | | | | | | | | |
| *Note.*  The intercept corresponds to the (unweighted) grand mean; for each factor with k levels, k - 1 parameters are estimated. Consequently, the estimates cannot be directly mapped to factor levels. Significant effects are highlighted in bold. | | | | | | | | | | | |

| Supplementary Table 10  *Results of linear mixed modeling with language, voice type, demographic factors as predictors and appeal as outcome* | | | | | | | | | | | |
| --- | --- | --- | --- | --- | --- | --- | --- | --- | --- | --- | --- |
| Factor | | Estimate | | *SE* | | *df* | | *t* | | *p* | |
| Intercept |  | 3.961 |  | 0.325 |  | 89.351 |  | 12.174 |  | < .001 |  |
| Language (1) |  | -0.276 |  | 0.317 |  | 86.821 |  | -0.870 |  | 0.387 |  |
| **Voice (1)** |  | **2.123** |  | **0.284** |  | **87.000** |  | **7.480** |  | **< .001** |  |
| **Voice (2)** |  | **-1.058** |  | **0.208** |  | **86.970** |  | **-5.098** |  | **< .001** |  |
| Gender (1) |  | 0.500 |  | 0.317 |  | 86.821 |  | 1.578 |  | 0.118 |  |
| Age |  | -0.008 |  | 0.010 |  | 86.821 |  | -0.769 |  | 0.444 |  |
| **Language (1) * Voice (1)** |  | **0.569** |  | **0.284** |  | **87.000** |  | **2.004** |  | **0.048** |  |
| Language (1) * Voice (2) |  | -0.374 |  | 0.208 |  | 86.970 |  | -1.800 |  | 0.075 |  |
| Language (1) * Gender (1) |  | -7.935e -5 |  | 0.317 |  | 86.821 |  | -2.504e -4 |  | 1.000 |  |
| Voice (1) * Gender (1) |  | -0.299 |  | 0.284 |  | 87.000 |  | -1.053 |  | 0.295 |  |
| **Voice (2) * Gender (1)** |  | **0.623** |  | **0.208** |  | **86.970** |  | **3.001** |  | **0.004** |  |
| Language (1) * Age |  | 0.004 |  | 0.010 |  | 86.821 |  | 0.382 |  | 0.703 |  |
| Voice (1) * Age |  | -0.008 |  | 0.009 |  | 87.000 |  | -0.840 |  | 0.403 |  |
| Voice (2) * Age |  | 0.002 |  | 0.007 |  | 86.970 |  | 0.297 |  | 0.767 |  |
| Gender (1) * Age |  | -0.011 |  | 0.010 |  | 86.821 |  | -1.021 |  | 0.310 |  |
| **Language (1) * Voice (1) * Gender (1)** |  | **-0.620** |  | **0.284** |  | **87.000** |  | **-2.186** |  | **0.032** |  |
| Language (1) * Voice (2) * Gender (1) |  | 0.367 |  | 0.208 |  | 86.970 |  | 1.767 |  | 0.081 |  |
| Language (1) * Voice (1) * Age |  | -0.009 |  | 0.009 |  | 87.000 |  | -0.956 |  | 0.342 |  |
| Language (1) * Voice (2) * Age |  | 0.009 |  | 0.007 |  | 86.970 |  | 1.387 |  | 0.169 |  |
| Language (1) * Gender (1) * Age |  | 0.003 |  | 0.010 |  | 86.821 |  | 0.310 |  | 0.757 |  |
| Voice (1) * Gender (1) * Age |  | -0.003 |  | 0.009 |  | 87.000 |  | -0.301 |  | 0.764 |  |
| **Voice (2) * Gender (1) * Age** |  | **-0.017** |  | **0.007** |  | **86.970** |  | **-2.500** |  | **0.014** |  |
| **Language (1) * Voice (1) * Gender (1) * Age** |  | **0.020** |  | **0.009** |  | **87.000** |  | **2.145** |  | **0.035** |  |
| Language (1) * Voice (2) * Gender (1) * Age |  | -0.006 |  | 0.007 |  | 86.970 |  | -0.833 |  | 0.407 |  |
|  | | | | | | | | | | | |
| *Note.*  The intercept corresponds to the (unweighted) grand mean; for each factor with k levels, k - 1 parameters are estimated. Consequently, the estimates cannot be directly mapped to factor levels. Significant effects are highlighted in bold. | | | | | | | | | | | |

| Supplementary Table 11  *Results of linear mixed modeling with language, voice type, demographic factors as predictors and credibility as outcome* | | | | | | | | | | | |
| --- | --- | --- | --- | --- | --- | --- | --- | --- | --- | --- | --- |
| Factor | | Estimate | | *SE* | | *df* | | *t* | | *p* | |
| Intercept |  | 3.811 |  | 0.316 |  | 87.841 |  | 12.067 |  | < .001 |  |
| Language (1) |  | -0.303 |  | 0.305 |  | 86.714 |  | -0.992 |  | 0.324 |  |
| **Voice (1)** |  | **2.043** |  | **0.177** |  | **1213.832** |  | **11.571** |  | **< .001** |  |
| **Voice (2)** |  | **-0.948** |  | **0.177** |  | **1213.832** |  | **-5.372** |  | **< .001** |  |
| Gender (1) |  | 0.600 |  | 0.305 |  | 86.714 |  | 1.966 |  | 0.052 |  |
| Age |  | -0.003 |  | 0.010 |  | 86.714 |  | -0.263 |  | 0.794 |  |
| **Language (1) * Voice (1)** |  | **0.447** |  | **0.177** |  | **1213.832** |  | **2.534** |  | **0.011** |  |
| **Language (1) * Voice (2)** |  | **-0.461** |  | **0.177** |  | **1213.832** |  | **-2.608** |  | **0.009** |  |
| Language (1) * Gender (1) |  | 0.126 |  | 0.305 |  | 86.714 |  | 0.413 |  | 0.681 |  |
| Voice (1) * Gender (1) |  | -0.244 |  | 0.177 |  | 1213.832 |  | -1.382 |  | 0.167 |  |
| **Voice (2) * Gender (1)** |  | **0.410** |  | **0.177** |  | **1213.832** |  | **2.320** |  | **0.021** |  |
| Language (1) * Age |  | 8.320e -4 |  | 0.010 |  | 86.714 |  | 0.083 |  | 0.934 |  |
| Voice (1) * Age |  | -0.004 |  | 0.006 |  | 1213.832 |  | -0.755 |  | 0.450 |  |
| Voice (2) * Age |  | -9.524e -4 |  | 0.006 |  | 1213.832 |  | -0.164 |  | 0.870 |  |
| Gender (1) * Age |  | -0.012 |  | 0.010 |  | 86.714 |  | -1.164 |  | 0.248 |  |
| **Language (1) * Voice (1) * Gender (1)** |  | **-0.441** |  | **0.177** |  | **1213.832** |  | **-2.496** |  | **0.013** |  |
| **Language (1) * Voice (2) * Gender (1)** |  | **0.384** |  | **0.177** |  | **1213.832** |  | **2.173** |  | **0.030** |  |
| Language (1) * Voice (1) * Age |  | -0.005 |  | 0.006 |  | 1213.832 |  | -0.843 |  | 0.399 |  |
| Language (1) * Voice (2) * Age |  | 0.011 |  | 0.006 |  | 1213.832 |  | 1.960 |  | 0.050 |  |
| Language (1) * Gender (1) * Age |  | -3.073e -4 |  | 0.010 |  | 86.714 |  | -0.031 |  | 0.976 |  |
| Voice (1) * Gender (1) * Age |  | -0.001 |  | 0.006 |  | 1213.832 |  | -0.196 |  | 0.844 |  |
| Voice (2) * Gender (1) * Age |  | -0.011 |  | 0.006 |  | 1213.832 |  | -1.953 |  | 0.051 |  |
| **Language (1) * Voice (1) * Gender (1) * Age** |  | **0.016** |  | **0.006** |  | **1213.832** |  | **2.740** |  | **0.006** |  |
| Language (1) * Voice (2) * Gender (1) * Age |  | -0.007 |  | 0.006 |  | 1213.832 |  | -1.160 |  | 0.246 |  |
|  | | | | | | | | | | | |
| *Note.*  The intercept corresponds to the (unweighted) grand mean; for each factor with k levels, k - 1 parameters are estimated. Consequently, the estimates cannot be directly mapped to factor levels. Significant effects are highlighted in bold. | | | | | | | | | | | |

| Supplementary Table 12  *Results of linear mixed modeling with language, voice type, demographic factors as predictors and human-likeness (personality) as outcome* | | | | | | | | | | | |
| --- | --- | --- | --- | --- | --- | --- | --- | --- | --- | --- | --- |
| Factor | | Estimate | | *SE* | | *df* | | *t* | | *p* | |
| Intercept |  | 3.691 |  | 0.255 |  | 78.839 |  | 14.490 |  | < .001 |  |
| **Language (1)** |  | **-0.984** |  | **0.240** |  | **86.569** |  | **-4.093** |  | **< .001** |  |
| **Voice (1)** |  | **3.139** |  | **0.197** |  | **1213.745** |  | **15.895** |  | **< .001** |  |
| **Voice (2)** |  | **-1.264** |  | **0.197** |  | **1213.745** |  | **-6.399** |  | **< .001** |  |
| Gender (1) |  | 0.459 |  | 0.240 |  | 86.569 |  | 1.910 |  | 0.059 |  |
| Age |  | -0.002 |  | 0.008 |  | 86.569 |  | -0.201 |  | 0.841 |  |
| **Language (1) * Voice (1)** |  | **0.904** |  | **0.197** |  | **1213.745** |  | **4.578** |  | **< .001** |  |
| **Language (1) * Voice (2)** |  | **-0.749** |  | **0.197** |  | **1213.745** |  | **-3.795** |  | **< .001** |  |
| Language (1) * Gender (1) |  | 0.121 |  | 0.240 |  | 86.569 |  | 0.502 |  | 0.617 |  |
| Voice (1) * Gender (1) |  | -0.305 |  | 0.197 |  | 1213.745 |  | -1.544 |  | 0.123 |  |
| **Voice (2) * Gender (1)** |  | **0.585** |  | **0.197** |  | **1213.745** |  | **2.964** |  | **0.003** |  |
| **Language (1) * Age** |  | **0.023** |  | **0.008** |  | **86.569** |  | **2.931** |  | **0.004** |  |
| **Voice (1) * Age** |  | **-0.014** |  | **0.006** |  | **1213.745** |  | **-2.083** |  | **0.037** |  |
| Voice (2) * Age |  | -6.856e -5 |  | 0.006 |  | 1213.745 |  | -0.011 |  | 0.992 |  |
| Gender (1) * Age |  | -0.009 |  | 0.008 |  | 86.569 |  | -1.177 |  | 0.242 |  |
| Language (1) * Voice (1) * Gender (1) |  | -0.358 |  | 0.197 |  | 1213.745 |  | -1.815 |  | 0.070 |  |
| **Language (1) * Voice (2) * Gender (1)** |  | **0.406** |  | **0.197** |  | **1213.745** |  | **2.057** |  | **0.040** |  |
| **Language (1) * Voice (1) * Age** |  | **-0.018** |  | **0.006** |  | **1213.745** |  | **-2.705** |  | **0.007** |  |
| **Language (1) * Voice (2) * Age** |  | **0.020** |  | **0.006** |  | **1213.745** |  | **3.033** |  | **0.002** |  |
| Language (1) * Gender (1) * Age |  | 0.001 |  | 0.008 |  | 86.569 |  | 0.131 |  | 0.896 |  |
| Voice (1) * Gender (1) * Age |  | 0.004 |  | 0.006 |  | 1213.745 |  | 0.647 |  | 0.518 |  |
| **Voice (2) * Gender (1) * Age** |  | **-0.019** |  | **0.006** |  | **1213.745** |  | **-2.931** |  | **0.003** |  |
| Language (1) * Voice (1) * Gender (1) * Age |  | 0.007 |  | 0.006 |  | 1213.745 |  | 1.142 |  | 0.254 |  |
| Language (1) * Voice (2) * Gender (1) * Age |  | -0.003 |  | 0.006 |  | 1213.745 |  | -0.481 |  | 0.631 |  |
|  | | | | | | | | | | | |
| *Note.*  The intercept corresponds to the (unweighted) grand mean; for each factor with k levels, k - 1 parameters are estimated. Consequently, the estimates cannot be directly mapped to factor levels. Significant effects are highlighted in bold. | | | | | | | | | | | |

| Supplementary Table 13  *Results of linear mixed modeling with language, voice type, demographic factors as predictors and eeriness as outcome* | | | | | | | | | | | |
| --- | --- | --- | --- | --- | --- | --- | --- | --- | --- | --- | --- |
| Factor | | Estimate | | *SE* | | *df* | | *t* | | *p* | |
| Intercept |  | 3.647 |  | 0.425 |  | 88.370 |  | 8.584 |  | < .001 |  |
| Language (1) |  | 0.421 |  | 0.422 |  | 86.957 |  | 0.999 |  | 0.321 |  |
| **Voice (1)** |  | **-1.498** |  | **0.211** |  | **1213.783** |  | **-7.093** |  | **< .001** |  |
| **Voice (2)** |  | **0.823** |  | **0.211** |  | **1213.783** |  | **3.898** |  | **< .001** |  |
| Gender (1) |  | -0.116 |  | 0.422 |  | 86.957 |  | -0.275 |  | 0.784 |  |
| Age |  | -0.010 |  | 0.014 |  | 86.957 |  | -0.725 |  | 0.470 |  |
| **Language (1) * Voice (1)** |  | **-0.434** |  | **0.211** |  | **1213.783** |  | **-2.055** |  | **0.040** |  |
| Language (1) * Voice (2) |  | 0.063 |  | 0.211 |  | 1213.783 |  | 0.296 |  | 0.767 |  |
| Language (1) * Gender (1) |  | -0.402 |  | 0.422 |  | 86.957 |  | -0.953 |  | 0.343 |  |
| Voice (1) * Gender (1) |  | -0.225 |  | 0.211 |  | 1213.783 |  | -1.065 |  | 0.287 |  |
| Voice (2) * Gender (1) |  | -0.002 |  | 0.211 |  | 1213.783 |  | -0.011 |  | 0.991 |  |
| Language (1) * Age |  | -0.006 |  | 0.014 |  | 86.957 |  | -0.467 |  | 0.642 |  |
| Voice (1) * Age |  | 0.003 |  | 0.007 |  | 1213.783 |  | 0.409 |  | 0.682 |  |
| Voice (2) * Age |  | -0.004 |  | 0.007 |  | 1213.783 |  | -0.580 |  | 0.562 |  |
| Gender (1) * Age |  | -0.006 |  | 0.014 |  | 86.957 |  | -0.466 |  | 0.642 |  |
| **Language (1) * Voice (1) * Gender (1)** |  | **0.609** |  | **0.211** |  | **1213.783** |  | **2.883** |  | **0.004** |  |
| **Language (1) * Voice (2) * Gender (1)** |  | **-0.581** |  | **0.211** |  | **1213.783** |  | **-2.750** |  | **0.006** |  |
| Language (1) * Voice (1) * Age |  | 0.008 |  | 0.007 |  | 1213.783 |  | 1.220 |  | 0.223 |  |
| Language (1) * Voice (2) * Age |  | 0.005 |  | 0.007 |  | 1213.783 |  | 0.785 |  | 0.433 |  |
| Language (1) * Gender (1) * Age |  | 0.018 |  | 0.014 |  | 86.957 |  | 1.316 |  | 0.192 |  |
| **Voice (1) * Gender (1) * Age** |  | **0.015** |  | **0.007** |  | **1213.783** |  | **2.208** |  | **0.027** |  |
| Voice (2) * Gender (1) * Age |  | -0.007 |  | 0.007 |  | 1213.783 |  | -0.962 |  | 0.336 |  |
| **Language (1) * Voice (1) * Gender (1) * Age** |  | **-0.026** |  | **0.007** |  | **1213.783** |  | **-3.744** |  | **< .001** |  |
| **Language (1) * Voice (2) * Gender (1) * Age** |  | **0.018** |  | **0.007** |  | **1213.783** |  | **2.576** |  | **0.010** |  |
|  | | | | | | | | | | | |
| *Note.*  The intercept corresponds to the (unweighted) grand mean; for each factor with k levels, k - 1 parameters are estimated. Consequently, the estimates cannot be directly mapped to factor levels. Significant effects are highlighted in bold. | | | | | | | | | | | |

Personality factors

| Supplementary Table 14  *Results of linear mixed modeling with language, voice type, personality factors as predictors and intelligibility as outcome* | | | | | | | | | | | |
| --- | --- | --- | --- | --- | --- | --- | --- | --- | --- | --- | --- |
| Factor | | Estimate | | *SE* | | *df* | | *t* | | *p* | |
| Intercept |  | 4.827 |  | 0.872 |  | 88.783 |  | 5.539 |  | < .001 |  |
| Language (1) |  | 0.072 |  | 0.869 |  | 87.794 |  | 0.083 |  | 0.934 |  |
| **Voice (1)** |  | **2.263** |  | **0.493** |  | **1227.809** |  | **4.591** |  | **< .001** |  |
| Voice (2) |  | -0.968 |  | 0.493 |  | 1227.809 |  | -1.964 |  | 0.050 |  |
| BFI_E |  | -0.098 |  | 0.122 |  | 87.794 |  | -0.799 |  | 0.426 |  |
| BFI_N |  | -0.004 |  | 0.111 |  | 87.794 |  | -0.036 |  | 0.971 |  |
| BFI_O |  | -0.078 |  | 0.156 |  | 87.794 |  | -0.502 |  | 0.617 |  |
| BFI_G |  | 0.022 |  | 0.101 |  | 87.794 |  | 0.215 |  | 0.830 |  |
| **BFI_V** |  | **0.312** |  | **0.138** |  | **87.794** |  | **2.255** |  | **0.027** |  |
| **Language (1) * Voice (1)** |  | **0.987** |  | **0.493** |  | **1227.809** |  | **2.002** |  | **0.046** |  |
| Language (1) * Voice (2) |  | -0.244 |  | 0.493 |  | 1227.809 |  | -0.496 |  | 0.620 |  |
| Language (1) * BFI_E |  | -0.136 |  | 0.122 |  | 87.794 |  | -1.114 |  | 0.268 |  |
| Voice (1) * BFI_E |  | 0.033 |  | 0.069 |  | 1227.809 |  | 0.481 |  | 0.631 |  |
| Voice (2) * BFI_E |  | 0.058 |  | 0.069 |  | 1227.809 |  | 0.836 |  | 0.403 |  |
| Language (1) * BFI_N |  | -0.133 |  | 0.111 |  | 87.794 |  | -1.200 |  | 0.233 |  |
| Voice (1) * BFI_N |  | -0.040 |  | 0.063 |  | 1227.809 |  | -0.629 |  | 0.529 |  |
| Voice (2) * BFI_N |  | 0.059 |  | 0.063 |  | 1227.809 |  | 0.934 |  | 0.350 |  |
| Language (1) * BFI_O |  | 0.138 |  | 0.156 |  | 87.794 |  | 0.886 |  | 0.378 |  |
| Voice (1) * BFI_O |  | 0.006 |  | 0.089 |  | 1227.809 |  | 0.064 |  | 0.949 |  |
| Voice (2) * BFI_O |  | -0.047 |  | 0.089 |  | 1227.809 |  | -0.529 |  | 0.597 |  |
| Language (1) * BFI_G |  | 0.058 |  | 0.101 |  | 87.794 |  | 0.579 |  | 0.564 |  |
| Voice (1) * BFI_G |  | -0.073 |  | 0.057 |  | 1227.809 |  | -1.270 |  | 0.204 |  |
| Voice (2) * BFI_G |  | -0.043 |  | 0.057 |  | 1227.809 |  | -0.742 |  | 0.458 |  |
| Language (1) * BFI_V |  | -0.047 |  | 0.138 |  | 87.794 |  | -0.337 |  | 0.737 |  |
| **Voice (1) * BFI_V** |  | **-0.223** |  | **0.078** |  | **1227.809** |  | **-2.840** |  | **0.005** |  |
| Voice (2) * BFI_V |  | 0.084 |  | 0.078 |  | 1227.809 |  | 1.072 |  | 0.284 |  |
| Language (1) * Voice (1) * BFI_E |  | 0.009 |  | 0.069 |  | 1227.809 |  | 0.128 |  | 0.898 |  |
| Language (1) * Voice (2) * BFI_E |  | 0.081 |  | 0.069 |  | 1227.809 |  | 1.169 |  | 0.243 |  |
| Language (1) * Voice (1) * BFI_N |  | 0.023 |  | 0.063 |  | 1227.809 |  | 0.361 |  | 0.718 |  |
| Language (1) * Voice (2) * BFI_N |  | 0.076 |  | 0.063 |  | 1227.809 |  | 1.205 |  | 0.229 |  |
| Language (1) * Voice (1) * BFI_O |  | -0.091 |  | 0.089 |  | 1227.809 |  | -1.027 |  | 0.305 |  |
| Language (1) * Voice (2) * BFI_O |  | -0.017 |  | 0.089 |  | 1227.809 |  | -0.193 |  | 0.847 |  |
| **Language (1) * Voice (1) * BFI_G** |  | **-0.115** |  | **0.057** |  | **1227.809** |  | **-2.008** |  | **0.045** |  |
| Language (1) * Voice (2) * BFI_G |  | 0.073 |  | 0.057 |  | 1227.809 |  | 1.270 |  | 0.204 |  |
| Language (1) * Voice (1) * BFI_V |  | -0.038 |  | 0.078 |  | 1227.809 |  | -0.478 |  | 0.633 |  |
| Language (1) * Voice (2) * BFI_V |  | -0.101 |  | 0.078 |  | 1227.809 |  | -1.292 |  | 0.197 |  |
|  | | | | | | | | | | | |
| *Note.*  The intercept corresponds to the (unweighted) grand mean; for each factor with k levels, k - 1 parameters are estimated. Consequently, the estimates cannot be directly mapped to factor levels. Significant effects are highlighted in bold. | | | | | | | | | | | |

| Supplementary Table 15  *Results of linear mixed modeling with language, voice type, personality factors as predictors and prosody as outcome* | | | | | | | | | | | |
| --- | --- | --- | --- | --- | --- | --- | --- | --- | --- | --- | --- |
| Factor | | Estimate | | *SE* | | *df* | | *t* | | *p* | |
| Intercept |  | 3.222 |  | 0.679 |  | 90.512 |  | 4.742 |  | < .001 |  |
| Language (1) |  | -0.695 |  | 0.674 |  | 87.720 |  | -1.031 |  | 0.305 |  |
| **Voice (1)** |  | **3.655** |  | **0.733** |  | **90.905** |  | **4.988** |  | **< .001** |  |
| **Voice (2)** |  | **-2.076** |  | **0.502** |  | **94.644** |  | **-4.138** |  | **< .001** |  |
| BFI_E |  | 0.030 |  | 0.095 |  | 87.651 |  | 0.315 |  | 0.754 |  |
| BFI_N |  | 0.082 |  | 0.086 |  | 87.651 |  | 0.951 |  | 0.344 |  |
| BFI_O |  | -0.018 |  | 0.121 |  | 87.678 |  | -0.145 |  | 0.885 |  |
| BFI_G |  | 0.072 |  | 0.078 |  | 87.664 |  | 0.922 |  | 0.359 |  |
| BFI_V |  | 0.174 |  | 0.107 |  | 87.674 |  | 1.621 |  | 0.109 |  |
| **Language (1) * Voice (1)** |  | **1.653** |  | **0.726** |  | **87.766** |  | **2.277** |  | **0.025** |  |
| Language (1) * Voice (2) |  | -0.925 |  | 0.490 |  | 87.393 |  | -1.888 |  | 0.062 |  |
| Language (1) * BFI_E |  | 0.017 |  | 0.095 |  | 87.651 |  | 0.183 |  | 0.856 |  |
| Voice (1) * BFI_E |  | 0.019 |  | 0.102 |  | 87.531 |  | 0.182 |  | 0.856 |  |
| Voice (2) * BFI_E |  | -0.026 |  | 0.069 |  | 87.263 |  | -0.383 |  | 0.702 |  |
| Language (1) * BFI_N |  | -0.010 |  | 0.086 |  | 87.651 |  | -0.119 |  | 0.905 |  |
| Voice (1) * BFI_N |  | -0.077 |  | 0.093 |  | 87.527 |  | -0.831 |  | 0.408 |  |
| Voice (2) * BFI_N |  | -0.006 |  | 0.063 |  | 87.261 |  | -0.094 |  | 0.925 |  |
| Language (1) * BFI_O |  | -0.064 |  | 0.121 |  | 87.678 |  | -0.531 |  | 0.596 |  |
| Voice (1) * BFI_O |  | -0.132 |  | 0.130 |  | 87.622 |  | -1.011 |  | 0.315 |  |
| Voice (2) * BFI_O |  | 0.130 |  | 0.088 |  | 87.313 |  | 1.473 |  | 0.144 |  |
| Language (1) * BFI_G |  | 0.086 |  | 0.078 |  | 87.664 |  | 1.099 |  | 0.275 |  |
| Voice (1) * BFI_G |  | -0.123 |  | 0.084 |  | 87.565 |  | -1.463 |  | 0.147 |  |
| Voice (2) * BFI_G |  | 0.092 |  | 0.057 |  | 87.285 |  | 1.624 |  | 0.108 |  |
| Language (1) * BFI_V |  | 0.144 |  | 0.107 |  | 87.674 |  | 1.344 |  | 0.183 |  |
| Voice (1) * BFI_V |  | -0.159 |  | 0.116 |  | 87.608 |  | -1.380 |  | 0.171 |  |
| Voice (2) * BFI_V |  | 0.140 |  | 0.078 |  | 87.305 |  | 1.802 |  | 0.075 |  |
| Language (1) * Voice (1) * BFI_E |  | 0.003 |  | 0.102 |  | 87.531 |  | 0.031 |  | 0.975 |  |
| Language (1) * Voice (2) * BFI_E |  | 0.011 |  | 0.069 |  | 87.263 |  | 0.154 |  | 0.878 |  |
| Language (1) * Voice (1) * BFI_N |  | -0.032 |  | 0.093 |  | 87.527 |  | -0.346 |  | 0.730 |  |
| Language (1) * Voice (2) * BFI_N |  | 0.015 |  | 0.063 |  | 87.261 |  | 0.246 |  | 0.806 |  |
| Language (1) * Voice (1) * BFI_O |  | -0.042 |  | 0.130 |  | 87.622 |  | -0.324 |  | 0.746 |  |
| Language (1) * Voice (2) * BFI_O |  | 0.045 |  | 0.088 |  | 87.313 |  | 0.516 |  | 0.607 |  |
| Language (1) * Voice (1) * BFI_G |  | -0.144 |  | 0.084 |  | 87.565 |  | -1.709 |  | 0.091 |  |
| **Language (1) * Voice (2) * BFI_G** |  | **0.136** |  | **0.057** |  | **87.285** |  | **2.399** |  | **0.019** |  |
| Language (1) * Voice (1) * BFI_V |  | -0.209 |  | 0.116 |  | 87.608 |  | -1.811 |  | 0.074 |  |
| Language (1) * Voice (2) * BFI_V |  | 0.062 |  | 0.078 |  | 87.305 |  | 0.794 |  | 0.429 |  |
|  | | | | | | | | | | | |
| *Note.*  The intercept corresponds to the (unweighted) grand mean; for each factor with k levels, k - 1 parameters are estimated. Consequently, the estimates cannot be directly mapped to factor levels. Significant effects are highlighted in bold. | | | | | | | | | | | |

| Supplementary Table 16  *Results of linear mixed modeling with language, voice type, personality factors as predictors and trustworthiness as outcome* | | | | | | | | | | | |
| --- | --- | --- | --- | --- | --- | --- | --- | --- | --- | --- | --- |
| Factor | | Estimate | | *SE* | | *df* | | *t* | | *p* | |
| Intercept |  | 2.374 |  | 0.814 |  | 90.045 |  | 2.916 |  | 0.004 |  |
| Language (1) |  | -0.051 |  | 0.807 |  | 87.659 |  | -0.063 |  | 0.950 |  |
| **Voice (1)** |  | **4.471** |  | **0.756** |  | **87.996** |  | **5.916** |  | **< .001** |  |
| **Voice (2)** |  | **-2.041** |  | **0.547** |  | **88.010** |  | **-3.729** |  | **< .001** |  |
| BFI_E |  | 0.067 |  | 0.114 |  | 87.659 |  | 0.590 |  | 0.557 |  |
| BFI_N |  | 0.144 |  | 0.103 |  | 87.659 |  | 1.399 |  | 0.165 |  |
| BFI_O |  | -0.037 |  | 0.145 |  | 87.659 |  | -0.256 |  | 0.798 |  |
| BFI_G |  | 0.150 |  | 0.094 |  | 87.659 |  | 1.603 |  | 0.112 |  |
| BFI_V |  | 0.194 |  | 0.129 |  | 87.659 |  | 1.513 |  | 0.134 |  |
| **Language (1) * Voice (1)** |  | **2.077** |  | **0.756** |  | **87.996** |  | **2.748** |  | **0.007** |  |
| **Language (1) * Voice (2)** |  | **-1.143** |  | **0.547** |  | **88.010** |  | **-2.089** |  | **0.040** |  |
| Language (1) * BFI_E |  | -0.019 |  | 0.114 |  | 87.659 |  | -0.163 |  | 0.871 |  |
| Voice (1) * BFI_E |  | 0.021 |  | 0.107 |  | 87.996 |  | 0.194 |  | 0.847 |  |
| Voice (2) * BFI_E |  | -0.065 |  | 0.077 |  | 88.010 |  | -0.848 |  | 0.399 |  |
| Language (1) * BFI_N |  | -0.009 |  | 0.103 |  | 87.659 |  | -0.091 |  | 0.928 |  |
| Voice (1) * BFI_N |  | -0.166 |  | 0.097 |  | 87.996 |  | -1.718 |  | 0.089 |  |
| Voice (2) * BFI_N |  | 0.066 |  | 0.070 |  | 88.010 |  | 0.940 |  | 0.350 |  |
| Language (1) * BFI_O |  | -0.128 |  | 0.145 |  | 87.659 |  | -0.881 |  | 0.381 |  |
| Voice (1) * BFI_O |  | -0.250 |  | 0.136 |  | 87.996 |  | -1.841 |  | 0.069 |  |
| Voice (2) * BFI_O |  | 0.065 |  | 0.098 |  | 88.010 |  | 0.661 |  | 0.510 |  |
| Language (1) * BFI_G |  | 0.052 |  | 0.094 |  | 87.659 |  | 0.552 |  | 0.582 |  |
| Voice (1) * BFI_G |  | -0.063 |  | 0.088 |  | 87.996 |  | -0.720 |  | 0.474 |  |
| Voice (2) * BFI_G |  | 0.090 |  | 0.064 |  | 88.010 |  | 1.419 |  | 0.159 |  |
| Language (1) * BFI_V |  | 0.066 |  | 0.129 |  | 87.659 |  | 0.516 |  | 0.607 |  |
| **Voice (1) * BFI_V** |  | **-0.306** |  | **0.120** |  | **87.996** |  | **-2.547** |  | **0.013** |  |
| **Voice (2) * BFI_V** |  | **0.177** |  | **0.087** |  | **88.010** |  | **2.030** |  | **0.045** |  |
| Language (1) * Voice (1) * BFI_E |  | 0.028 |  | 0.107 |  | 87.996 |  | 0.258 |  | 0.797 |  |
| Language (1) * Voice (2) * BFI_E |  | -0.053 |  | 0.077 |  | 88.010 |  | -0.684 |  | 0.496 |  |
| Language (1) * Voice (1) * BFI_N |  | 0.075 |  | 0.097 |  | 87.996 |  | 0.777 |  | 0.439 |  |
| Language (1) * Voice (2) * BFI_N |  | 0.059 |  | 0.070 |  | 88.010 |  | 0.848 |  | 0.399 |  |
| Language (1) * Voice (1) * BFI_O |  | -0.196 |  | 0.136 |  | 87.996 |  | -1.440 |  | 0.154 |  |
| Language (1) * Voice (2) * BFI_O |  | 0.004 |  | 0.098 |  | 88.010 |  | 0.042 |  | 0.967 |  |
| **Language (1) * Voice (1) * BFI_G** |  | **-0.224** |  | **0.088** |  | **87.996** |  | **-2.547** |  | **0.013** |  |
| **Language (1) * Voice (2) * BFI_G** |  | **0.252** |  | **0.064** |  | **88.010** |  | **3.961** |  | **< .001** |  |
| Language (1) * Voice (1) * BFI_V |  | -0.172 |  | 0.120 |  | 87.996 |  | -1.429 |  | 0.157 |  |
| Language (1) * Voice (2) * BFI_V |  | 0.057 |  | 0.087 |  | 88.010 |  | 0.650 |  | 0.518 |  |
|  | | | | | | | | | | | |
| *Note.*  The intercept corresponds to the (unweighted) grand mean; for each factor with k levels, k - 1 parameters are estimated. Consequently, the estimates cannot be directly mapped to factor levels. Significant effects are highlighted in bold. | | | | | | | | | | | |

| Supplementary Table 17  *Results of linear mixed modeling with language, voice type, personality factors as predictors and confidence as outcome* | | | | | | | | | | | |
| --- | --- | --- | --- | --- | --- | --- | --- | --- | --- | --- | --- |
| Factor | | Estimate | | *SE* | | *df* | | *t* | | *p* | |
| Intercept |  | 2.783 |  | 0.800 |  | 90.439 |  | 3.478 |  | < .001 |  |
| Language (1) |  | -0.175 |  | 0.792 |  | 87.676 |  | -0.222 |  | 0.825 |  |
| **Voice (1)** |  | **4.154** |  | **0.784** |  | **88.003** |  | **5.300** |  | **< .001** |  |
| **Voice (2)** |  | **-1.799** |  | **0.558** |  | **88.011** |  | **-3.221** |  | **0.002** |  |
| BFI_E |  | 0.054 |  | 0.112 |  | 87.676 |  | 0.483 |  | 0.630 |  |
| BFI_N |  | 0.200 |  | 0.101 |  | 87.676 |  | 1.977 |  | 0.051 |  |
| BFI_O |  | -0.073 |  | 0.142 |  | 87.676 |  | -0.516 |  | 0.607 |  |
| BFI_G |  | 0.133 |  | 0.092 |  | 87.676 |  | 1.441 |  | 0.153 |  |
| BFI_V |  | 0.165 |  | 0.126 |  | 87.676 |  | 1.305 |  | 0.195 |  |
| **Language (1) * Voice (1)** |  | **1.594** |  | **0.784** |  | **88.003** |  | **2.033** |  | **0.045** |  |
| Language (1) * Voice (2) |  | -0.631 |  | 0.558 |  | 88.011 |  | -1.130 |  | 0.261 |  |
| Language (1) * BFI_E |  | -0.124 |  | 0.112 |  | 87.676 |  | -1.115 |  | 0.268 |  |
| Voice (1) * BFI_E |  | -0.021 |  | 0.110 |  | 88.003 |  | -0.193 |  | 0.848 |  |
| Voice (2) * BFI_E |  | -0.032 |  | 0.079 |  | 88.011 |  | -0.401 |  | 0.690 |  |
| Language (1) * BFI_N |  | -0.051 |  | 0.101 |  | 87.676 |  | -0.500 |  | 0.618 |  |
| Voice (1) * BFI_N |  | -0.182 |  | 0.100 |  | 88.003 |  | -1.817 |  | 0.073 |  |
| Voice (2) * BFI_N |  | 0.010 |  | 0.071 |  | 88.011 |  | 0.138 |  | 0.891 |  |
| Language (1) * BFI_O |  | -0.011 |  | 0.142 |  | 87.676 |  | -0.074 |  | 0.941 |  |
| Voice (1) * BFI_O |  | -0.219 |  | 0.141 |  | 88.003 |  | -1.554 |  | 0.124 |  |
| Voice (2) * BFI_O |  | 0.113 |  | 0.100 |  | 88.011 |  | 1.129 |  | 0.262 |  |
| Language (1) * BFI_G |  | 0.169 |  | 0.092 |  | 87.676 |  | 1.835 |  | 0.070 |  |
| Voice (1) * BFI_G |  | -0.021 |  | 0.091 |  | 88.003 |  | -0.226 |  | 0.822 |  |
| Voice (2) * BFI_G |  | 0.057 |  | 0.065 |  | 88.011 |  | 0.877 |  | 0.383 |  |
| Language (1) * BFI_V |  | -0.013 |  | 0.126 |  | 87.676 |  | -0.099 |  | 0.921 |  |
| Voice (1) * BFI_V |  | -0.205 |  | 0.125 |  | 88.003 |  | -1.641 |  | 0.104 |  |
| Voice (2) * BFI_V |  | 0.099 |  | 0.089 |  | 88.011 |  | 1.118 |  | 0.267 |  |
| Language (1) * Voice (1) * BFI_E |  | 0.047 |  | 0.110 |  | 88.003 |  | 0.426 |  | 0.671 |  |
| Language (1) * Voice (2) * BFI_E |  | -0.027 |  | 0.079 |  | 88.011 |  | -0.347 |  | 0.729 |  |
| Language (1) * Voice (1) * BFI_N |  | 0.073 |  | 0.100 |  | 88.003 |  | 0.730 |  | 0.467 |  |
| Language (1) * Voice (2) * BFI_N |  | -0.013 |  | 0.071 |  | 88.011 |  | -0.186 |  | 0.853 |  |
| Language (1) * Voice (1) * BFI_O |  | -0.147 |  | 0.141 |  | 88.003 |  | -1.044 |  | 0.299 |  |
| Language (1) * Voice (2) * BFI_O |  | 0.032 |  | 0.100 |  | 88.011 |  | 0.323 |  | 0.748 |  |
| **Language (1) * Voice (1) * BFI_G** |  | **-0.220** |  | **0.091** |  | **88.003** |  | **-2.415** |  | **0.018** |  |
| **Language (1) * Voice (2) * BFI_G** |  | **0.209** |  | **0.065** |  | **88.011** |  | **3.215** |  | **0.002** |  |
| Language (1) * Voice (1) * BFI_V |  | -0.117 |  | 0.125 |  | 88.003 |  | -0.937 |  | 0.351 |  |
| Language (1) * Voice (2) * BFI_V |  | -0.023 |  | 0.089 |  | 88.011 |  | -0.255 |  | 0.800 |  |
|  | | | | | | | | | | | |
| *Note.*  The intercept corresponds to the (unweighted) grand mean; for each factor with k levels, k - 1 parameters are estimated. Consequently, the estimates cannot be directly mapped to factor levels. Significant effects are highlighted in bold. | | | | | | | | | | | |

| Supplementary Table 18  *Results of linear mixed modeling with language, voice type, personality factors as predictors and enthusiasm as outcome* | | | | | | | | | | | |
| --- | --- | --- | --- | --- | --- | --- | --- | --- | --- | --- | --- |
| Factor | | Estimate | | *SE* | | *df* | | *t* | | *p* | |
| Intercept |  | 3.071 |  | 0.899 |  | 90.039 |  | 3.415 |  | < .001 |  |
| Language (1) |  | -0.555 |  | 0.892 |  | 87.700 |  | -0.623 |  | 0.535 |  |
| **Voice (1)** |  | **3.919** |  | **0.810** |  | **88.002** |  | **4.836** |  | **< .001** |  |
| **Voice (2)** |  | **-1.522** |  | **0.585** |  | **88.003** |  | **-2.602** |  | **0.011** |  |
| BFI_E |  | -0.013 |  | 0.126 |  | 87.700 |  | -0.101 |  | 0.920 |  |
| BFI_N |  | 0.155 |  | 0.114 |  | 87.700 |  | 1.360 |  | 0.177 |  |
| BFI_O |  | 2.121e -4 |  | 0.160 |  | 87.700 |  | 0.001 |  | 0.999 |  |
| BFI_G |  | 0.075 |  | 0.104 |  | 87.700 |  | 0.720 |  | 0.473 |  |
| BFI_V |  | 0.065 |  | 0.142 |  | 87.700 |  | 0.457 |  | 0.649 |  |
| Language (1) * Voice (1) |  | 1.523 |  | 0.810 |  | 88.002 |  | 1.879 |  | 0.064 |  |
| Language (1) * Voice (2) |  | -0.415 |  | 0.585 |  | 88.003 |  | -0.709 |  | 0.480 |  |
| Language (1) * BFI_E |  | -0.041 |  | 0.126 |  | 87.700 |  | -0.330 |  | 0.743 |  |
| Voice (1) * BFI_E |  | 0.049 |  | 0.114 |  | 88.002 |  | 0.433 |  | 0.666 |  |
| Voice (2) * BFI_E |  | -0.108 |  | 0.082 |  | 88.003 |  | -1.307 |  | 0.195 |  |
| Language (1) * BFI_N |  | 0.009 |  | 0.114 |  | 87.700 |  | 0.081 |  | 0.936 |  |
| Voice (1) * BFI_N |  | -0.136 |  | 0.104 |  | 88.002 |  | -1.317 |  | 0.191 |  |
| Voice (2) * BFI_N |  | -0.014 |  | 0.075 |  | 88.003 |  | -0.183 |  | 0.855 |  |
| Language (1) * BFI_O |  | 0.092 |  | 0.160 |  | 87.700 |  | 0.574 |  | 0.568 |  |
| Voice (1) * BFI_O |  | -0.258 |  | 0.146 |  | 88.002 |  | -1.767 |  | 0.081 |  |
| Voice (2) * BFI_O |  | 0.012 |  | 0.105 |  | 88.003 |  | 0.114 |  | 0.909 |  |
| Language (1) * BFI_G |  | 0.143 |  | 0.104 |  | 87.700 |  | 1.377 |  | 0.172 |  |
| Voice (1) * BFI_G |  | -0.098 |  | 0.094 |  | 88.002 |  | -1.038 |  | 0.302 |  |
| **Voice (2) * BFI_G** |  | **0.160** |  | **0.068** |  | **88.003** |  | **2.348** |  | **0.021** |  |
| Language (1) * BFI_V |  | -0.063 |  | 0.142 |  | 87.700 |  | -0.445 |  | 0.657 |  |
| Voice (1) * BFI_V |  | -0.123 |  | 0.129 |  | 88.002 |  | -0.951 |  | 0.344 |  |
| Voice (2) * BFI_V |  | 0.059 |  | 0.093 |  | 88.003 |  | 0.631 |  | 0.530 |  |
| Language (1) * Voice (1) * BFI_E |  | 0.058 |  | 0.114 |  | 88.002 |  | 0.512 |  | 0.610 |  |
| Language (1) * Voice (2) * BFI_E |  | -0.059 |  | 0.082 |  | 88.003 |  | -0.716 |  | 0.476 |  |
| Language (1) * Voice (1) * BFI_N |  | 0.008 |  | 0.104 |  | 88.002 |  | 0.079 |  | 0.937 |  |
| Language (1) * Voice (2) * BFI_N |  | 0.054 |  | 0.075 |  | 88.003 |  | 0.718 |  | 0.474 |  |
| Language (1) * Voice (1) * BFI_O |  | -0.104 |  | 0.146 |  | 88.002 |  | -0.710 |  | 0.479 |  |
| Language (1) * Voice (2) * BFI_O |  | -0.020 |  | 0.105 |  | 88.003 |  | -0.190 |  | 0.850 |  |
| **Language (1) * Voice (1) * BFI_G** |  | **-0.208** |  | **0.094** |  | **88.002** |  | **-2.213** |  | **0.029** |  |
| **Language (1) * Voice (2) * BFI_G** |  | **0.179** |  | **0.068** |  | **88.003** |  | **2.633** |  | **0.010** |  |
| Language (1) * Voice (1) * BFI_V |  | -0.114 |  | 0.129 |  | 88.002 |  | -0.880 |  | 0.381 |  |
| Language (1) * Voice (2) * BFI_V |  | -0.026 |  | 0.093 |  | 88.003 |  | -0.278 |  | 0.782 |  |
|  | | | | | | | | | | | |
| *Note.*  The intercept corresponds to the (unweighted) grand mean; for each factor with k levels, k - 1 parameters are estimated. Consequently, the estimates cannot be directly mapped to factor levels. Significant effects are highlighted in bold. | | | | | | | | | | | |

| Supplementary Table 19  *Results of linear mixed modeling with language, voice type, personality factors as predictors and pleasantness as outcome* | | | | | | | | | | | |
| --- | --- | --- | --- | --- | --- | --- | --- | --- | --- | --- | --- |
| Factor | | Estimate | | *SE* | | *df* | | *t* | | *p* | |
| Intercept |  | 2.963 |  | 0.849 |  | 89.815 |  | 3.492 |  | < .001 |  |
| Language (1) |  | 0.002 |  | 0.842 |  | 87.654 |  | 0.002 |  | 0.998 |  |
| **Voice (1)** |  | **4.218** |  | **0.778** |  | **87.997** |  | **5.425** |  | **< .001** |  |
| **Voice (2)** |  | **-1.896** |  | **0.618** |  | **87.990** |  | **-3.068** |  | **0.003** |  |
| BFI_E |  | 0.037 |  | 0.119 |  | 87.654 |  | 0.313 |  | 0.755 |  |
| BFI_N |  | 0.113 |  | 0.108 |  | 87.654 |  | 1.052 |  | 0.296 |  |
| BFI_O |  | -0.105 |  | 0.151 |  | 87.654 |  | -0.696 |  | 0.489 |  |
| BFI_G |  | 0.124 |  | 0.098 |  | 87.654 |  | 1.263 |  | 0.210 |  |
| BFI_V |  | 0.190 |  | 0.134 |  | 87.654 |  | 1.413 |  | 0.161 |  |
| **Language (1) * Voice (1)** |  | **2.008** |  | **0.778** |  | **87.997** |  | **2.583** |  | **0.011** |  |
| Language (1) * Voice (2) |  | -0.884 |  | 0.618 |  | 87.990 |  | -1.431 |  | 0.156 |  |
| Language (1) * BFI_E |  | -0.022 |  | 0.119 |  | 87.654 |  | -0.187 |  | 0.852 |  |
| Voice (1) * BFI_E |  | -0.011 |  | 0.110 |  | 87.997 |  | -0.098 |  | 0.922 |  |
| Voice (2) * BFI_E |  | 0.015 |  | 0.087 |  | 87.990 |  | 0.168 |  | 0.867 |  |
| Language (1) * BFI_N |  | -2.109e -4 |  | 0.108 |  | 87.654 |  | -0.002 |  | 0.998 |  |
| Voice (1) * BFI_N |  | -0.135 |  | 0.099 |  | 87.997 |  | -1.363 |  | 0.176 |  |
| Voice (2) * BFI_N |  | -0.037 |  | 0.079 |  | 87.990 |  | -0.470 |  | 0.640 |  |
| Language (1) * BFI_O |  | -0.089 |  | 0.151 |  | 87.654 |  | -0.590 |  | 0.557 |  |
| Voice (1) * BFI_O |  | -0.154 |  | 0.140 |  | 87.997 |  | -1.101 |  | 0.274 |  |
| Voice (2) * BFI_O |  | 0.009 |  | 0.111 |  | 87.990 |  | 0.083 |  | 0.934 |  |
| Language (1) * BFI_G |  | 0.013 |  | 0.098 |  | 87.654 |  | 0.130 |  | 0.897 |  |
| Voice (1) * BFI_G |  | -0.096 |  | 0.090 |  | 87.997 |  | -1.064 |  | 0.290 |  |
| Voice (2) * BFI_G |  | 0.084 |  | 0.072 |  | 87.990 |  | 1.171 |  | 0.245 |  |
| Language (1) * BFI_V |  | 0.055 |  | 0.134 |  | 87.654 |  | 0.411 |  | 0.682 |  |
| **Voice (1) * BFI_V** |  | **-0.347** |  | **0.124** |  | **87.997** |  | **-2.807** |  | **0.006** |  |
| **Voice (2) * BFI_V** |  | **0.213** |  | **0.098** |  | **87.990** |  | **2.161** |  | **0.033** |  |
| Language (1) * Voice (1) * BFI_E |  | -0.011 |  | 0.110 |  | 87.997 |  | -0.097 |  | 0.923 |  |
| Language (1) * Voice (2) * BFI_E |  | 0.006 |  | 0.087 |  | 87.990 |  | 0.067 |  | 0.946 |  |
| Language (1) * Voice (1) * BFI_N |  | 0.015 |  | 0.099 |  | 87.997 |  | 0.155 |  | 0.877 |  |
| Language (1) * Voice (2) * BFI_N |  | 0.005 |  | 0.079 |  | 87.990 |  | 0.063 |  | 0.950 |  |
| Language (1) * Voice (1) * BFI_O |  | -0.185 |  | 0.140 |  | 87.997 |  | -1.325 |  | 0.188 |  |
| Language (1) * Voice (2) * BFI_O |  | 0.087 |  | 0.111 |  | 87.990 |  | 0.785 |  | 0.435 |  |
| Language (1) * Voice (1) * BFI_G |  | -0.160 |  | 0.090 |  | 87.997 |  | -1.775 |  | 0.079 |  |
| **Language (1) * Voice (2) * BFI_G** |  | **0.158** |  | **0.072** |  | **87.990** |  | **2.207** |  | **0.030** |  |
| Language (1) * Voice (1) * BFI_V |  | -0.189 |  | 0.124 |  | 87.997 |  | -1.526 |  | 0.131 |  |
| Language (1) * Voice (2) * BFI_V |  | 0.007 |  | 0.098 |  | 87.990 |  | 0.067 |  | 0.946 |  |
|  | | | | | | | | | | | |
| *Note.*  The intercept corresponds to the (unweighted) grand mean; for each factor with k levels, k - 1 parameters are estimated. Consequently, the estimates cannot be directly mapped to factor levels. Significant effects are highlighted in bold. | | | | | | | | | | | |

| Supplementary Table 20  *Results of linear mixed modeling with language, voice type, personality factors as predictors and naturalness as outcome* | | | | | | | | | | | |
| --- | --- | --- | --- | --- | --- | --- | --- | --- | --- | --- | --- |
| Factor | | Estimate | | *SE* | | *df* | | *t* | | *p* | |
| Intercept |  | 3.171 |  | 0.702 |  | 89.443 |  | 4.519 |  | < .001 |  |
| Language (1) |  | -0.206 |  | 0.697 |  | 87.612 |  | -0.296 |  | 0.768 |  |
| **Voice (1)** |  | **4.344** |  | **0.536** |  | **1226.792** |  | **8.098** |  | **< .001** |  |
| **Voice (2)** |  | **-2.173** |  | **0.536** |  | **1226.792** |  | **-4.051** |  | **< .001** |  |
| BFI_E |  | -0.054 |  | 0.098 |  | 87.609 |  | -0.550 |  | 0.584 |  |
| BFI_N |  | 0.014 |  | 0.089 |  | 87.612 |  | 0.152 |  | 0.880 |  |
| BFI_O |  | -0.097 |  | 0.125 |  | 87.609 |  | -0.774 |  | 0.441 |  |
| BFI_G |  | 0.157 |  | 0.081 |  | 87.636 |  | 1.938 |  | 0.056 |  |
| BFI_V |  | 0.189 |  | 0.111 |  | 87.611 |  | 1.701 |  | 0.092 |  |
| **Language (1) * Voice (1)** |  | **2.079** |  | **0.536** |  | **1226.792** |  | **3.875** |  | **< .001** |  |
| Language (1) * Voice (2) |  | -1.043 |  | 0.536 |  | 1226.792 |  | -1.944 |  | 0.052 |  |
| Language (1) * BFI_E |  | -0.045 |  | 0.098 |  | 87.609 |  | -0.458 |  | 0.648 |  |
| Voice (1) * BFI_E |  | -0.013 |  | 0.076 |  | 1226.790 |  | -0.170 |  | 0.865 |  |
| Voice (2) * BFI_E |  | -0.030 |  | 0.076 |  | 1226.790 |  | -0.395 |  | 0.693 |  |
| Language (1) * BFI_N |  | -0.071 |  | 0.089 |  | 87.612 |  | -0.792 |  | 0.430 |  |
| **Voice (1) * BFI_N** |  | **-0.155** |  | **0.069** |  | **1226.792** |  | **-2.259** |  | **0.024** |  |
| Voice (2) * BFI_N |  | 0.031 |  | 0.069 |  | 1226.792 |  | 0.454 |  | 0.650 |  |
| Language (1) * BFI_O |  | -0.079 |  | 0.125 |  | 87.609 |  | -0.631 |  | 0.530 |  |
| Voice (1) * BFI_O |  | -0.057 |  | 0.096 |  | 1226.791 |  | -0.592 |  | 0.554 |  |
| Voice (2) * BFI_O |  | 0.042 |  | 0.096 |  | 1226.791 |  | 0.437 |  | 0.662 |  |
| Language (1) * BFI_G |  | 0.101 |  | 0.081 |  | 87.636 |  | 1.249 |  | 0.215 |  |
| **Voice (1) * BFI_G** |  | **-0.173** |  | **0.062** |  | **1226.804** |  | **-2.776** |  | **0.006** |  |
| **Voice (2) * BFI_G** |  | **0.152** |  | **0.062** |  | **1226.804** |  | **2.434** |  | **0.015** |  |
| Language (1) * BFI_V |  | 0.088 |  | 0.111 |  | 87.611 |  | 0.792 |  | 0.431 |  |
| **Voice (1) * BFI_V** |  | **-0.178** |  | **0.085** |  | **1226.792** |  | **-2.088** |  | **0.037** |  |
| Voice (2) * BFI_V |  | 0.127 |  | 0.085 |  | 1226.792 |  | 1.492 |  | 0.136 |  |
| Language (1) * Voice (1) * BFI_E |  | -0.080 |  | 0.076 |  | 1226.790 |  | -1.055 |  | 0.292 |  |
| Language (1) * Voice (2) * BFI_E |  | 0.027 |  | 0.076 |  | 1226.790 |  | 0.353 |  | 0.724 |  |
| Language (1) * Voice (1) * BFI_N |  | -0.094 |  | 0.069 |  | 1226.792 |  | -1.369 |  | 0.171 |  |
| Language (1) * Voice (2) * BFI_N |  | 0.043 |  | 0.069 |  | 1226.792 |  | 0.623 |  | 0.533 |  |
| Language (1) * Voice (1) * BFI_O |  | -0.039 |  | 0.096 |  | 1226.791 |  | -0.402 |  | 0.688 |  |
| Language (1) * Voice (2) * BFI_O |  | 0.024 |  | 0.096 |  | 1226.791 |  | 0.253 |  | 0.800 |  |
| **Language (1) * Voice (1) * BFI_G** |  | **-0.218** |  | **0.062** |  | **1226.804** |  | **-3.496** |  | **< .001** |  |
| **Language (1) * Voice (2) * BFI_G** |  | **0.207** |  | **0.062** |  | **1226.804** |  | **3.324** |  | **< .001** |  |
| Language (1) * Voice (1) * BFI_V |  | -0.130 |  | 0.085 |  | 1226.792 |  | -1.525 |  | 0.128 |  |
| Language (1) * Voice (2) * BFI_V |  | 0.001 |  | 0.085 |  | 1226.792 |  | 0.017 |  | 0.986 |  |
|  | | | | | | | | | | | |
| *Note.*  The intercept corresponds to the (unweighted) grand mean; for each factor with k levels, k - 1 parameters are estimated. Consequently, the estimates cannot be directly mapped to factor levels. Significant effects are highlighted in bold. | | | | | | | | | | | |

| Supplementary Table 21  *Results of linear mixed modeling with language, voice type, personality factors as predictors and human-likeness (voice) as outcome* | | | | | | | | | | | |
| --- | --- | --- | --- | --- | --- | --- | --- | --- | --- | --- | --- |
| Factor | | Estimate | | *SE* | | *df* | | *t* | | *p* | |
| Intercept |  | 3.028 |  | 0.727 |  | 89.776 |  | 4.166 |  | < .001 |  |
| Language (1) |  | -0.428 |  | 0.722 |  | 87.806 |  | -0.593 |  | 0.555 |  |
| **Voice (1)** |  | **3.864** |  | **0.848** |  | **87.992** |  | **4.555** |  | **< .001** |  |
| **Voice (2)** |  | **-1.653** |  | **0.605** |  | **88.001** |  | **-2.730** |  | **0.008** |  |
| BFI_E |  | -0.029 |  | 0.102 |  | 87.806 |  | -0.290 |  | 0.773 |  |
| BFI_N |  | 0.036 |  | 0.092 |  | 87.806 |  | 0.392 |  | 0.696 |  |
| BFI_O |  | -0.058 |  | 0.130 |  | 87.806 |  | -0.449 |  | 0.654 |  |
| **BFI_G** |  | **0.172** |  | **0.084** |  | **87.806** |  | **2.050** |  | **0.043** |  |
| BFI_V |  | 0.172 |  | 0.115 |  | 87.806 |  | 1.500 |  | 0.137 |  |
| Language (1) * Voice (1) |  | 1.113 |  | 0.848 |  | 87.992 |  | 1.311 |  | 0.193 |  |
| Language (1) * Voice (2) |  | -0.524 |  | 0.605 |  | 88.001 |  | -0.865 |  | 0.390 |  |
| Language (1) * BFI_E |  | -0.040 |  | 0.102 |  | 87.806 |  | -0.396 |  | 0.693 |  |
| Voice (1) * BFI_E |  | 0.046 |  | 0.120 |  | 87.991 |  | 0.384 |  | 0.702 |  |
| Voice (2) * BFI_E |  | -0.066 |  | 0.085 |  | 88.001 |  | -0.776 |  | 0.440 |  |
| Language (1) * BFI_N |  | -0.080 |  | 0.092 |  | 87.806 |  | -0.872 |  | 0.386 |  |
| Voice (1) * BFI_N |  | -0.058 |  | 0.108 |  | 87.992 |  | -0.539 |  | 0.591 |  |
| Voice (2) * BFI_N |  | 0.034 |  | 0.077 |  | 88.001 |  | 0.438 |  | 0.663 |  |
| Language (1) * BFI_O |  | -0.050 |  | 0.130 |  | 87.806 |  | -0.384 |  | 0.702 |  |
| Voice (1) * BFI_O |  | -0.129 |  | 0.153 |  | 87.992 |  | -0.844 |  | 0.401 |  |
| Voice (2) * BFI_O |  | 0.023 |  | 0.109 |  | 88.001 |  | 0.213 |  | 0.832 |  |
| **Language (1) * BFI_G** |  | **0.198** |  | **0.084** |  | **87.806** |  | **2.367** |  | **0.020** |  |
| Voice (1) * BFI_G |  | -0.180 |  | 0.099 |  | 87.991 |  | -1.831 |  | 0.070 |  |
| **Voice (2) * BFI_G** |  | **0.156** |  | **0.070** |  | **88.001** |  | **2.223** |  | **0.029** |  |
| Language (1) * BFI_V |  | 0.040 |  | 0.115 |  | 87.806 |  | 0.344 |  | 0.731 |  |
| Voice (1) * BFI_V |  | -0.055 |  | 0.135 |  | 87.992 |  | -0.411 |  | 0.682 |  |
| Voice (2) * BFI_V |  | 0.005 |  | 0.096 |  | 88.001 |  | 0.056 |  | 0.955 |  |
| Language (1) * Voice (1) * BFI_E |  | -0.005 |  | 0.120 |  | 87.991 |  | -0.043 |  | 0.966 |  |
| Language (1) * Voice (2) * BFI_E |  | -0.002 |  | 0.085 |  | 88.001 |  | -0.027 |  | 0.979 |  |
| Language (1) * Voice (1) * BFI_N |  | 0.028 |  | 0.108 |  | 87.992 |  | 0.259 |  | 0.796 |  |
| Language (1) * Voice (2) * BFI_N |  | 0.041 |  | 0.077 |  | 88.001 |  | 0.525 |  | 0.601 |  |
| Language (1) * Voice (1) * BFI_O |  | -0.063 |  | 0.153 |  | 87.992 |  | -0.410 |  | 0.683 |  |
| Language (1) * Voice (2) * BFI_O |  | -0.023 |  | 0.109 |  | 88.001 |  | -0.208 |  | 0.835 |  |
| Language (1) * Voice (1) * BFI_G |  | -0.196 |  | 0.099 |  | 87.991 |  | -1.984 |  | 0.050 |  |
| **Language (1) * Voice (2) * BFI_G** |  | **0.223** |  | **0.070** |  | **88.001** |  | **3.164** |  | **0.002** |  |
| Language (1) * Voice (1) * BFI_V |  | -0.012 |  | 0.135 |  | 87.991 |  | -0.089 |  | 0.929 |  |
| Language (1) * Voice (2) * BFI_V |  | -0.102 |  | 0.096 |  | 88.001 |  | -1.060 |  | 0.292 |  |
|  | | | | | | | | | | | |
| *Note.*  The intercept corresponds to the (unweighted) grand mean; for each factor with k levels, k - 1 parameters are estimated. Consequently, the estimates cannot be directly mapped to factor levels. Significant effects are highlighted in bold. | | | | | | | | | | | |

| Supplementary Table 22  *Results of linear mixed modeling with language, voice type, personality factors as predictors and likability as outcome* | | | | | | | | | | | |
| --- | --- | --- | --- | --- | --- | --- | --- | --- | --- | --- | --- |
| Factor | | Estimate | | *SE* | | *df* | | *t* | | *p* | |
| Intercept |  | 2.202 |  | 0.866 |  | 89.262 |  | 2.544 |  | 0.013 |  |
| Language (1) |  | 0.034 |  | 0.861 |  | 87.707 |  | 0.039 |  | 0.969 |  |
| **Voice (1)** |  | **4.186** |  | **0.850** |  | **87.999** |  | **4.926** |  | **< .001** |  |
| **Voice (2)** |  | **-1.507** |  | **0.682** |  | **87.999** |  | **-2.211** |  | **0.030** |  |
| BFI_E |  | 0.110 |  | 0.121 |  | 87.707 |  | 0.909 |  | 0.366 |  |
| BFI_N |  | 0.196 |  | 0.110 |  | 87.707 |  | 1.784 |  | 0.078 |  |
| BFI_O |  | -0.072 |  | 0.155 |  | 87.707 |  | -0.462 |  | 0.645 |  |
| BFI_G |  | 0.156 |  | 0.100 |  | 87.707 |  | 1.560 |  | 0.122 |  |
| BFI_V |  | 0.129 |  | 0.137 |  | 87.707 |  | 0.939 |  | 0.351 |  |
| **Language (1) * Voice (1)** |  | **1.998** |  | **0.850** |  | **87.999** |  | **2.351** |  | **0.021** |  |
| Language (1) * Voice (2) |  | -0.819 |  | 0.682 |  | 87.999 |  | -1.201 |  | 0.233 |  |
| Language (1) * BFI_E |  | 0.021 |  | 0.121 |  | 87.707 |  | 0.176 |  | 0.861 |  |
| Voice (1) * BFI_E |  | 0.012 |  | 0.120 |  | 87.999 |  | 0.097 |  | 0.923 |  |
| Voice (2) * BFI_E |  | -0.076 |  | 0.096 |  | 87.999 |  | -0.788 |  | 0.433 |  |
| Language (1) * BFI_N |  | 0.018 |  | 0.110 |  | 87.707 |  | 0.160 |  | 0.874 |  |
| Voice (1) * BFI_N |  | -0.106 |  | 0.109 |  | 87.999 |  | -0.980 |  | 0.330 |  |
| Voice (2) * BFI_N |  | -0.076 |  | 0.087 |  | 87.999 |  | -0.878 |  | 0.382 |  |
| Language (1) * BFI_O |  | -0.090 |  | 0.155 |  | 87.707 |  | -0.581 |  | 0.563 |  |
| Voice (1) * BFI_O |  | -0.176 |  | 0.153 |  | 87.999 |  | -1.150 |  | 0.253 |  |
| Voice (2) * BFI_O |  | 0.036 |  | 0.123 |  | 87.999 |  | 0.294 |  | 0.769 |  |
| Language (1) * BFI_G |  | -0.060 |  | 0.100 |  | 87.707 |  | -0.603 |  | 0.548 |  |
| Voice (1) * BFI_G |  | -0.146 |  | 0.099 |  | 87.999 |  | -1.478 |  | 0.143 |  |
| Voice (2) * BFI_G |  | 0.084 |  | 0.079 |  | 87.999 |  | 1.060 |  | 0.292 |  |
| Language (1) * BFI_V |  | 0.052 |  | 0.137 |  | 87.707 |  | 0.379 |  | 0.706 |  |
| **Voice (1) * BFI_V** |  | **-0.275** |  | **0.135** |  | **87.999** |  | **-2.035** |  | **0.045** |  |
| Voice (2) * BFI_V |  | 0.165 |  | 0.109 |  | 87.999 |  | 1.522 |  | 0.132 |  |
| Language (1) * Voice (1) * BFI_E |  | 0.055 |  | 0.120 |  | 87.999 |  | 0.462 |  | 0.645 |  |
| Language (1) * Voice (2) * BFI_E |  | -0.105 |  | 0.096 |  | 87.999 |  | -1.094 |  | 0.277 |  |
| Language (1) * Voice (1) * BFI_N |  | 0.087 |  | 0.109 |  | 87.999 |  | 0.797 |  | 0.428 |  |
| Language (1) * Voice (2) * BFI_N |  | -0.014 |  | 0.087 |  | 87.999 |  | -0.161 |  | 0.873 |  |
| Language (1) * Voice (1) * BFI_O |  | -0.224 |  | 0.153 |  | 87.999 |  | -1.463 |  | 0.147 |  |
| Language (1) * Voice (2) * BFI_O |  | 0.089 |  | 0.123 |  | 87.999 |  | 0.722 |  | 0.472 |  |
| **Language (1) * Voice (1) * BFI_G** |  | **-0.252** |  | **0.099** |  | **87.999** |  | **-2.549** |  | **0.013** |  |
| **Language (1) * Voice (2) * BFI_G** |  | **0.177** |  | **0.079** |  | **87.999** |  | **2.236** |  | **0.028** |  |
| Language (1) * Voice (1) * BFI_V |  | -0.142 |  | 0.135 |  | 87.999 |  | -1.053 |  | 0.295 |  |
| Language (1) * Voice (2) * BFI_V |  | 0.049 |  | 0.109 |  | 87.999 |  | 0.454 |  | 0.651 |  |
|  | | | | | | | | | | | |
| *Note.*  The intercept corresponds to the (unweighted) grand mean; for each factor with k levels, k - 1 parameters are estimated. Consequently, the estimates cannot be directly mapped to factor levels. Significant effects are highlighted in bold. | | | | | | | | | | | |

| Supplementary Table 23  *Results of linear mixed modeling with language, voice type, personality factors as predictors and appeal as outcome* | | | | | | | | | | | |
| --- | --- | --- | --- | --- | --- | --- | --- | --- | --- | --- | --- |
| Factor | | Estimate | | *SE* | | *df* | | *t* | | *p* | |
| Intercept |  | 2.466 |  | 0.861 |  | 88.918 |  | 2.865 |  | 0.005 |  |
| Language (1) |  | 0.003 |  | 0.858 |  | 87.776 |  | 0.003 |  | 0.998 |  |
| **Voice (1)** |  | **4.139** |  | **0.754** |  | **87.993** |  | **5.492** |  | **< .001** |  |
| **Voice (2)** |  | **-1.756** |  | **0.561** |  | **88.003** |  | **-3.129** |  | **0.002** |  |
| BFI_E |  | 0.059 |  | 0.121 |  | 87.776 |  | 0.488 |  | 0.627 |  |
| BFI_N |  | 0.168 |  | 0.110 |  | 87.776 |  | 1.530 |  | 0.130 |  |
| BFI_O |  | -0.100 |  | 0.154 |  | 87.776 |  | -0.646 |  | 0.520 |  |
| BFI_G |  | 0.139 |  | 0.100 |  | 87.776 |  | 1.400 |  | 0.165 |  |
| BFI_V |  | 0.182 |  | 0.137 |  | 87.776 |  | 1.331 |  | 0.187 |  |
| **Language (1) * Voice (1)** |  | **2.131** |  | **0.754** |  | **87.993** |  | **2.828** |  | **0.006** |  |
| Language (1) * Voice (2) |  | -1.005 |  | 0.561 |  | 88.003 |  | -1.791 |  | 0.077 |  |
| Language (1) * BFI_E |  | -0.003 |  | 0.121 |  | 87.776 |  | -0.021 |  | 0.984 |  |
| Voice (1) * BFI_E |  | 0.052 |  | 0.106 |  | 87.993 |  | 0.492 |  | 0.624 |  |
| Voice (2) * BFI_E |  | -0.054 |  | 0.079 |  | 88.003 |  | -0.680 |  | 0.498 |  |
| Language (1) * BFI_N |  | 0.024 |  | 0.110 |  | 87.776 |  | 0.219 |  | 0.827 |  |
| Voice (1) * BFI_N |  | -0.131 |  | 0.096 |  | 87.993 |  | -1.361 |  | 0.177 |  |
| Voice (2) * BFI_N |  | -0.010 |  | 0.072 |  | 88.003 |  | -0.144 |  | 0.886 |  |
| Language (1) * BFI_O |  | -0.127 |  | 0.154 |  | 87.776 |  | -0.827 |  | 0.411 |  |
| Voice (1) * BFI_O |  | -0.209 |  | 0.136 |  | 87.993 |  | -1.540 |  | 0.127 |  |
| Voice (2) * BFI_O |  | 0.061 |  | 0.101 |  | 88.003 |  | 0.604 |  | 0.548 |  |
| Language (1) * BFI_G |  | 0.006 |  | 0.100 |  | 87.776 |  | 0.064 |  | 0.949 |  |
| Voice (1) * BFI_G |  | -0.052 |  | 0.088 |  | 87.993 |  | -0.595 |  | 0.554 |  |
| Voice (2) * BFI_G |  | 0.054 |  | 0.065 |  | 88.003 |  | 0.827 |  | 0.410 |  |
| Language (1) * BFI_V |  | 0.070 |  | 0.137 |  | 87.776 |  | 0.513 |  | 0.610 |  |
| **Voice (1) * BFI_V** |  | **-0.341** |  | **0.120** |  | **87.993** |  | **-2.844** |  | **0.006** |  |
| Voice (2) * BFI_V |  | 0.176 |  | 0.089 |  | 88.003 |  | 1.970 |  | 0.052 |  |
| Language (1) * Voice (1) * BFI_E |  | -0.005 |  | 0.106 |  | 87.993 |  | -0.049 |  | 0.961 |  |
| Language (1) * Voice (2) * BFI_E |  | -0.041 |  | 0.079 |  | 88.003 |  | -0.518 |  | 0.606 |  |
| Language (1) * Voice (1) * BFI_N |  | 0.076 |  | 0.096 |  | 87.993 |  | 0.793 |  | 0.430 |  |
| Language (1) * Voice (2) * BFI_N |  | -0.003 |  | 0.072 |  | 88.003 |  | -0.039 |  | 0.969 |  |
| **Language (1) * Voice (1) * BFI_O** |  | **-0.294** |  | **0.136** |  | **87.993** |  | **-2.170** |  | **0.033** |  |
| Language (1) * Voice (2) * BFI_O |  | 0.129 |  | 0.101 |  | 88.003 |  | 1.274 |  | 0.206 |  |
| **Language (1) * Voice (1) * BFI_G** |  | **-0.204** |  | **0.088** |  | **87.993** |  | **-2.326** |  | **0.022** |  |
| **Language (1) * Voice (2) * BFI_G** |  | **0.173** |  | **0.065** |  | **88.003** |  | **2.657** |  | **0.009** |  |
| Language (1) * Voice (1) * BFI_V |  | -0.087 |  | 0.120 |  | 87.993 |  | -0.722 |  | 0.473 |  |
| Language (1) * Voice (2) * BFI_V |  | 0.005 |  | 0.089 |  | 88.003 |  | 0.056 |  | 0.956 |  |
|  | | | | | | | | | | | |
| *Note.*  The intercept corresponds to the (unweighted) grand mean; for each factor with k levels, k - 1 parameters are estimated. Consequently, the estimates cannot be directly mapped to factor levels. Significant effects are highlighted in bold. | | | | | | | | | | | |

| Supplementary Table 24  *Results of linear mixed modeling with language, voice type, personality factors as predictors and credibility as outcome* | | | | | | | | | | | |
| --- | --- | --- | --- | --- | --- | --- | --- | --- | --- | --- | --- |
| Factor | | *Estimate* | | *SE* | | *df* | | *t* | | *p* | |
| Intercept |  | 2.136 |  | 0.830 |  | 89.166 |  | 2.573 |  | 0.012 |  |
| Language (1) |  | -0.499 |  | 0.826 |  | 87.692 |  | -0.604 |  | 0.547 |  |
| **Voice (1)** |  | **3.752** |  | **0.485** |  | **1227.835** |  | **7.739** |  | **< .001** |  |
| **Voice (2)** |  | **-1.597** |  | **0.485** |  | **1227.835** |  | **-3.295** |  | **0.001** |  |
| BFI_E |  | -5.073e -5 |  | 0.116 |  | 87.692 |  | -4.357e -4 |  | 1.000 |  |
| BFI_N |  | 0.137 |  | 0.106 |  | 87.692 |  | 1.296 |  | 0.198 |  |
| BFI_O |  | -0.039 |  | 0.149 |  | 87.692 |  | -0.265 |  | 0.791 |  |
| BFI_G |  | 0.156 |  | 0.096 |  | 87.692 |  | 1.626 |  | 0.108 |  |
| **BFI_V** |  | **0.269** |  | **0.132** |  | **87.692** |  | **2.042** |  | **0.044** |  |
| **Language (1) * Voice (1)** |  | **1.868** |  | **0.485** |  | **1227.835** |  | **3.853** |  | **< .001** |  |
| **Language (1) * Voice (2)** |  | **-1.048** |  | **0.485** |  | **1227.835** |  | **-2.162** |  | **0.031** |  |
| Language (1) * BFI_E |  | -0.078 |  | 0.116 |  | 87.692 |  | -0.670 |  | 0.504 |  |
| Voice (1) * BFI_E |  | 0.059 |  | 0.068 |  | 1227.835 |  | 0.862 |  | 0.389 |  |
| Voice (2) * BFI_E |  | -0.079 |  | 0.068 |  | 1227.835 |  | -1.162 |  | 0.246 |  |
| Language (1) * BFI_N |  | -0.017 |  | 0.106 |  | 87.692 |  | -0.164 |  | 0.870 |  |
| Voice (1) * BFI_N |  | -0.114 |  | 0.062 |  | 1227.835 |  | -1.835 |  | 0.067 |  |
| Voice (2) * BFI_N |  | 0.009 |  | 0.062 |  | 1227.835 |  | 0.139 |  | 0.889 |  |
| Language (1) * BFI_O |  | -0.023 |  | 0.149 |  | 87.692 |  | -0.152 |  | 0.880 |  |
| Voice (1) * BFI_O |  | -0.156 |  | 0.087 |  | 1227.835 |  | -1.785 |  | 0.075 |  |
| Voice (2) * BFI_O |  | 0.060 |  | 0.087 |  | 1227.835 |  | 0.683 |  | 0.495 |  |
| Language (1) * BFI_G |  | 0.050 |  | 0.096 |  | 87.692 |  | 0.525 |  | 0.601 |  |
| Voice (1) * BFI_G |  | -0.032 |  | 0.056 |  | 1227.835 |  | -0.565 |  | 0.572 |  |
| Voice (2) * BFI_G |  | 0.076 |  | 0.056 |  | 1227.835 |  | 1.352 |  | 0.177 |  |
| Language (1) * BFI_V |  | 0.132 |  | 0.132 |  | 87.692 |  | 1.007 |  | 0.317 |  |
| **Voice (1) * BFI_V** |  | **-0.311** |  | **0.077** |  | **1227.835** |  | **-4.023** |  | **< .001** |  |
| Voice (2) * BFI_V |  | 0.123 |  | 0.077 |  | 1227.835 |  | 1.600 |  | 0.110 |  |
| Language (1) * Voice (1) * BFI_E |  | 0.008 |  | 0.068 |  | 1227.835 |  | 0.111 |  | 0.912 |  |
| Language (1) * Voice (2) * BFI_E |  | -0.053 |  | 0.068 |  | 1227.835 |  | -0.779 |  | 0.436 |  |
| Language (1) * Voice (1) * BFI_N |  | 0.046 |  | 0.062 |  | 1227.835 |  | 0.735 |  | 0.462 |  |
| Language (1) * Voice (2) * BFI_N |  | 0.018 |  | 0.062 |  | 1227.835 |  | 0.284 |  | 0.777 |  |
| **Language (1) * Voice (1) * BFI_O** |  | **-0.194** |  | **0.087** |  | **1227.835** |  | **-2.223** |  | **0.026** |  |
| Language (1) * Voice (2) * BFI_O |  | 0.058 |  | 0.087 |  | 1227.835 |  | 0.668 |  | 0.504 |  |
| **Language (1) * Voice (1) * BFI_G** |  | **-0.240** |  | **0.056** |  | **1227.835** |  | **-4.256** |  | **< .001** |  |
| **Language (1) * Voice (2) * BFI_G** |  | **0.234** |  | **0.056** |  | **1227.835** |  | **4.162** |  | **< .001** |  |
| Language (1) * Voice (1) * BFI_V |  | -0.056 |  | 0.077 |  | 1227.835 |  | -0.719 |  | 0.472 |  |
| Language (1) * Voice (2) * BFI_V |  | 0.013 |  | 0.077 |  | 1227.835 |  | 0.165 |  | 0.869 |  |
|  | | | | | | | | | | | |
| *Note.*  The intercept corresponds to the (unweighted) grand mean; for each factor with k levels, k - 1 parameters are estimated. Consequently, the estimates cannot be directly mapped to factor levels. Significant effects are highlighted in bold. | | | | | | | | | | | |

| Supplementary Table 25  *Results of linear mixed modeling with language, voice type, personality factors as predictors and human-likeness (personality) as outcome* | | | | | | | | | | | |
| --- | --- | --- | --- | --- | --- | --- | --- | --- | --- | --- | --- |
| Factor | | Estimate | | *SE* | | *df* | | *t* | | *p* | |
| Intercept |  | 2.936 |  | 0.680 |  | 89.892 |  | 4.316 |  | < .001 |  |
| Language (1) |  | -0.236 |  | 0.675 |  | 87.734 |  | -0.349 |  | 0.728 |  |
| **Voice (1)** |  | **4.061** |  | **0.872** |  | **87.981** |  | **4.655** |  | **< .001** |  |
| **Voice (2)** |  | **-1.784** |  | **0.635** |  | **87.989** |  | **-2.809** |  | **0.006** |  |
| BFI_E |  | -0.018 |  | 0.095 |  | 87.734 |  | -0.193 |  | 0.848 |  |
| BFI_N |  | 0.041 |  | 0.086 |  | 87.734 |  | 0.470 |  | 0.639 |  |
| BFI_O |  | -0.057 |  | 0.121 |  | 87.734 |  | -0.468 |  | 0.641 |  |
| BFI_G |  | 0.153 |  | 0.078 |  | 87.734 |  | 1.948 |  | 0.055 |  |
| BFI_V |  | 0.168 |  | 0.107 |  | 87.734 |  | 1.568 |  | 0.121 |  |
| Language (1) * Voice (1) |  | 1.565 |  | 0.872 |  | 87.981 |  | 1.793 |  | 0.076 |  |
| Language (1) * Voice (2) |  | -0.964 |  | 0.635 |  | 87.989 |  | -1.518 |  | 0.133 |  |
| Language (1) * BFI_E |  | -0.025 |  | 0.095 |  | 87.734 |  | -0.267 |  | 0.790 |  |
| Voice (1) * BFI_E |  | 0.074 |  | 0.123 |  | 87.981 |  | 0.599 |  | 0.550 |  |
| Voice (2) * BFI_E |  | -0.099 |  | 0.090 |  | 87.989 |  | -1.101 |  | 0.274 |  |
| Language (1) * BFI_N |  | -0.075 |  | 0.086 |  | 87.734 |  | -0.872 |  | 0.385 |  |
| Voice (1) * BFI_N |  | -0.071 |  | 0.112 |  | 87.981 |  | -0.636 |  | 0.526 |  |
| Voice (2) * BFI_N |  | -0.022 |  | 0.081 |  | 87.989 |  | -0.267 |  | 0.790 |  |
| Language (1) * BFI_O |  | -0.007 |  | 0.121 |  | 87.734 |  | -0.059 |  | 0.953 |  |
| Voice (1) * BFI_O |  | -0.126 |  | 0.157 |  | 87.981 |  | -0.802 |  | 0.425 |  |
| Voice (2) * BFI_O |  | 0.067 |  | 0.114 |  | 87.989 |  | 0.588 |  | 0.558 |  |
| Language (1) * BFI_G |  | 0.117 |  | 0.078 |  | 87.734 |  | 1.486 |  | 0.141 |  |
| Voice (1) * BFI_G |  | -0.180 |  | 0.101 |  | 87.981 |  | -1.779 |  | 0.079 |  |
| Voice (2) * BFI_G |  | 0.145 |  | 0.074 |  | 87.989 |  | 1.972 |  | 0.052 |  |
| Language (1) * BFI_V |  | -0.016 |  | 0.107 |  | 87.734 |  | -0.144 |  | 0.885 |  |
| Voice (1) * BFI_V |  | -0.136 |  | 0.139 |  | 87.981 |  | -0.977 |  | 0.331 |  |
| Voice (2) * BFI_V |  | 0.083 |  | 0.101 |  | 87.989 |  | 0.820 |  | 0.414 |  |
| Language (1) * Voice (1) * BFI_E |  | -0.031 |  | 0.123 |  | 87.981 |  | -0.249 |  | 0.804 |  |
| Language (1) * Voice (2) * BFI_E |  | -0.033 |  | 0.090 |  | 87.989 |  | -0.372 |  | 0.711 |  |
| Language (1) * Voice (1) * BFI_N |  | -0.020 |  | 0.112 |  | 87.981 |  | -0.177 |  | 0.860 |  |
| Language (1) * Voice (2) * BFI_N |  | -0.014 |  | 0.081 |  | 87.989 |  | -0.176 |  | 0.861 |  |
| Language (1) * Voice (1) * BFI_O |  | -0.049 |  | 0.157 |  | 87.981 |  | -0.313 |  | 0.755 |  |
| Language (1) * Voice (2) * BFI_O |  | 0.018 |  | 0.114 |  | 87.989 |  | 0.160 |  | 0.873 |  |
| **Language (1) * Voice (1) * BFI_G** |  | **-0.215** |  | **0.101** |  | **87.981** |  | **-2.124** |  | **0.036** |  |
| **Language (1) * Voice (2) * BFI_G** |  | **0.226** |  | **0.074** |  | **87.989** |  | **3.069** |  | **0.003** |  |
| Language (1) * Voice (1) * BFI_V |  | -0.066 |  | 0.139 |  | 87.981 |  | -0.472 |  | 0.638 |  |
| Language (1) * Voice (2) * BFI_V |  | 0.045 |  | 0.101 |  | 87.989 |  | 0.449 |  | 0.654 |  |
|  | | | | | | | | | | | |
| *Note.*  The intercept corresponds to the (unweighted) grand mean; for each factor with k levels, k - 1 parameters are estimated. Consequently, the estimates cannot be directly mapped to factor levels. Significant effects are highlighted in bold. | | | | | | | | | | | |

| Supplementary Table 26  *Results of linear mixed modeling with language, voice type, personality factors as predictors and eeriness as outcome* | | | | | | | | | | | |
| --- | --- | --- | --- | --- | --- | --- | --- | --- | --- | --- | --- |
| Factor | | Estimate | | *SE* | | *df* | | *t* | | *p* | |
| Intercept |  | 3.961 |  | 1.116 |  | 88.287 |  | 3.548 |  | < .001 |  |
| Language (1) |  | -0.428 |  | 1.115 |  | 87.962 |  | -0.383 |  | 0.702 |  |
| **Voice (1)** |  | **-2.941** |  | **1.022** |  | **87.999** |  | **-2.879** |  | **0.005** |  |
| Voice (2) |  | 0.998 |  | 0.646 |  | 88.027 |  | 1.545 |  | 0.126 |  |
| BFI_E |  | -0.036 |  | 0.157 |  | 87.962 |  | -0.227 |  | 0.821 |  |
| BFI_N |  | -0.214 |  | 0.143 |  | 87.962 |  | -1.499 |  | 0.137 |  |
| BFI_O |  | 0.315 |  | 0.201 |  | 87.962 |  | 1.570 |  | 0.120 |  |
| BFI_G |  | -0.215 |  | 0.130 |  | 87.962 |  | -1.662 |  | 0.100 |  |
| BFI_V |  | -0.157 |  | 0.178 |  | 87.962 |  | -0.884 |  | 0.379 |  |
| **Language (1) * Voice (1)** |  | **-2.730** |  | **1.022** |  | **87.999** |  | **-2.672** |  | **0.009** |  |
| Language (1) * Voice (2) |  | 1.167 |  | 0.646 |  | 88.027 |  | 1.807 |  | 0.074 |  |
| Language (1) * BFI_E |  | -0.020 |  | 0.157 |  | 87.962 |  | -0.128 |  | 0.899 |  |
| Voice (1) * BFI_E |  | 0.044 |  | 0.144 |  | 87.999 |  | 0.302 |  | 0.763 |  |
| Voice (2) * BFI_E |  | 0.090 |  | 0.091 |  | 88.027 |  | 0.994 |  | 0.323 |  |
| Language (1) * BFI_N |  | 0.090 |  | 0.143 |  | 87.962 |  | 0.635 |  | 0.527 |  |
| Voice (1) * BFI_N |  | 0.167 |  | 0.131 |  | 87.999 |  | 1.281 |  | 0.204 |  |
| Voice (2) * BFI_N |  | -0.027 |  | 0.083 |  | 88.027 |  | -0.326 |  | 0.745 |  |
| Language (1) * BFI_O |  | 0.295 |  | 0.201 |  | 87.962 |  | 1.469 |  | 0.145 |  |
| Voice (1) * BFI_O |  | -0.060 |  | 0.184 |  | 87.999 |  | -0.326 |  | 0.745 |  |
| Voice (2) * BFI_O |  | 0.010 |  | 0.116 |  | 88.027 |  | 0.090 |  | 0.929 |  |
| Language (1) * BFI_G |  | -0.257 |  | 0.130 |  | 87.962 |  | -1.985 |  | 0.050 |  |
| Voice (1) * BFI_G |  | -0.031 |  | 0.119 |  | 87.999 |  | -0.257 |  | 0.798 |  |
| Voice (2) * BFI_G |  | -0.006 |  | 0.075 |  | 88.027 |  | -0.076 |  | 0.939 |  |
| Language (1) * BFI_V |  | 0.016 |  | 0.178 |  | 87.962 |  | 0.088 |  | 0.930 |  |
| **Voice (1) * BFI_V** |  | **0.403** |  | **0.163** |  | **87.999** |  | **2.478** |  | **0.015** |  |
| Voice (2) * BFI_V |  | -0.146 |  | 0.103 |  | 88.027 |  | -1.417 |  | 0.160 |  |
| Language (1) * Voice (1) * BFI_E |  | 0.101 |  | 0.144 |  | 87.999 |  | 0.702 |  | 0.485 |  |
| Language (1) * Voice (2) * BFI_E |  | 0.021 |  | 0.091 |  | 88.027 |  | 0.228 |  | 0.820 |  |
| Language (1) * Voice (1) * BFI_N |  | 0.061 |  | 0.131 |  | 87.999 |  | 0.465 |  | 0.643 |  |
| Language (1) * Voice (2) * BFI_N |  | 0.026 |  | 0.083 |  | 88.027 |  | 0.318 |  | 0.751 |  |
| Language (1) * Voice (1) * BFI_O |  | 0.041 |  | 0.184 |  | 87.999 |  | 0.221 |  | 0.826 |  |
| Language (1) * Voice (2) * BFI_O |  | -0.047 |  | 0.116 |  | 88.027 |  | -0.403 |  | 0.688 |  |
| **Language (1) * Voice (1) * BFI_G** |  | **0.297** |  | **0.119** |  | **87.999** |  | **2.505** |  | **0.014** |  |
| **Language (1) * Voice (2) * BFI_G** |  | **-0.161** |  | **0.075** |  | **88.027** |  | **-2.141** |  | **0.035** |  |
| Language (1) * Voice (1) * BFI_V |  | 0.295 |  | 0.163 |  | 87.999 |  | 1.814 |  | 0.073 |  |
| Language (1) * Voice (2) * BFI_V |  | -0.125 |  | 0.103 |  | 88.027 |  | -1.220 |  | 0.226 |  |
|  | | | | | | | | | | | |
| *Note.*  The intercept corresponds to the (unweighted) grand mean; for each factor with k levels, k - 1 parameters are estimated. Consequently, the estimates cannot be directly mapped to factor levels. Significant effects are highlighted in bold. | | | | | | | | | | | |

Robots-related factors

| Supplementary Table 27  *Results of linear mixed modeling with language, voice type, robots-related factors as predictors and intelligibility as outcome* | | | | | | | | | | | |
| --- | --- | --- | --- | --- | --- | --- | --- | --- | --- | --- | --- |
| Factor | | Estimate | | *SE* | | *df* | | *t* | | *p* | |
| Intercept |  | 3.495 |  | 5.561 |  | 87.788 |  | 0.628 |  | 0.531 |  |
| Language (1) |  | -3.535 |  | 5.561 |  | 87.761 |  | -0.636 |  | 0.527 |  |
| Voice (1) |  | -0.784 |  | 3.357 |  | 1227.796 |  | -0.233 |  | 0.815 |  |
| Voice (2) |  | 0.118 |  | 3.357 |  | 1227.796 |  | 0.035 |  | 0.972 |  |
| NARS |  | 0.815 |  | 1.481 |  | 87.761 |  | 0.550 |  | 0.584 |  |
| Interest |  | 1.813 |  | 2.826 |  | 87.761 |  | 0.642 |  | 0.523 |  |
| Exposure |  | 0.311 |  | 1.730 |  | 87.761 |  | 0.180 |  | 0.858 |  |
| Language (1) * Voice (1) |  | -1.277 |  | 3.357 |  | 1227.796 |  | -0.380 |  | 0.704 |  |
| Language (1) * Voice (2) |  | 0.220 |  | 3.357 |  | 1227.796 |  | 0.066 |  | 0.948 |  |
| Language (1) * NARS |  | 0.887 |  | 1.481 |  | 87.761 |  | 0.599 |  | 0.551 |  |
| Voice (1) * NARS |  | 0.356 |  | 0.894 |  | 1227.796 |  | 0.398 |  | 0.691 |  |
| Voice (2) * NARS |  | -0.053 |  | 0.894 |  | 1227.796 |  | -0.060 |  | 0.953 |  |
| Language (1) * Interest |  | 1.895 |  | 2.826 |  | 87.761 |  | 0.670 |  | 0.504 |  |
| Voice (1) * Interest |  | 0.475 |  | 1.706 |  | 1227.796 |  | 0.279 |  | 0.781 |  |
| Voice (2) * Interest |  | -0.103 |  | 1.706 |  | 1227.796 |  | -0.061 |  | 0.952 |  |
| NARS * Interest |  | -0.597 |  | 0.758 |  | 87.761 |  | -0.788 |  | 0.433 |  |
| Language (1) * Exposure |  | 0.699 |  | 1.730 |  | 87.761 |  | 0.404 |  | 0.687 |  |
| Voice (1) * Exposure |  | 1.256 |  | 1.044 |  | 1227.796 |  | 1.203 |  | 0.229 |  |
| Voice (2) * Exposure |  | 0.137 |  | 1.044 |  | 1227.796 |  | 0.131 |  | 0.896 |  |
| NARS * Exposure |  | -0.206 |  | 0.463 |  | 87.761 |  | -0.445 |  | 0.657 |  |
| Interest * Exposure |  | -0.356 |  | 0.881 |  | 87.761 |  | -0.404 |  | 0.687 |  |
| Language (1) * Voice (1) * NARS |  | 0.315 |  | 0.894 |  | 1227.796 |  | 0.353 |  | 0.724 |  |
| Language (1) * Voice (2) * NARS |  | 0.021 |  | 0.894 |  | 1227.796 |  | 0.023 |  | 0.981 |  |
| Language (1) * Voice (1) * Interest |  | 0.661 |  | 1.706 |  | 1227.796 |  | 0.387 |  | 0.699 |  |
| Language (1) * Voice (2) * Interest |  | -0.236 |  | 1.706 |  | 1227.796 |  | -0.138 |  | 0.890 |  |
| Language (1) * NARS * Interest |  | -0.421 |  | 0.758 |  | 87.761 |  | -0.556 |  | 0.580 |  |
| Voice (1) * NARS * Interest |  | -0.064 |  | 0.458 |  | 1227.796 |  | -0.140 |  | 0.889 |  |
| Voice (2) * NARS * Interest |  | -0.021 |  | 0.458 |  | 1227.796 |  | -0.046 |  | 0.963 |  |
| Language (1) * Voice (1) * Exposure |  | 0.983 |  | 1.044 |  | 1227.796 |  | 0.942 |  | 0.347 |  |
| Language (1) * Voice (2) * Exposure |  | 0.617 |  | 1.044 |  | 1227.796 |  | 0.591 |  | 0.554 |  |
| Language (1) * NARS * Exposure |  | -0.192 |  | 0.463 |  | 87.761 |  | -0.415 |  | 0.679 |  |
| Voice (1) * NARS * Exposure |  | -0.278 |  | 0.279 |  | 1227.796 |  | -0.993 |  | 0.321 |  |
| Voice (2) * NARS * Exposure |  | -0.094 |  | 0.279 |  | 1227.796 |  | -0.336 |  | 0.737 |  |
| Language (1) * Interest * Exposure |  | -0.407 |  | 0.881 |  | 87.761 |  | -0.462 |  | 0.645 |  |
| Voice (1) * Interest * Exposure |  | -0.590 |  | 0.532 |  | 1227.796 |  | -1.110 |  | 0.267 |  |
| Voice (2) * Interest * Exposure |  | -0.072 |  | 0.532 |  | 1227.796 |  | -0.135 |  | 0.892 |  |
| NARS * Interest * Exposure |  | 0.138 |  | 0.237 |  | 87.761 |  | 0.583 |  | 0.561 |  |
| Language (1) * Voice (1) * NARS * Interest |  | -0.181 |  | 0.458 |  | 1227.796 |  | -0.396 |  | 0.692 |  |
| Language (1) * Voice (2) * NARS * Interest |  | 0.084 |  | 0.458 |  | 1227.796 |  | 0.184 |  | 0.854 |  |
| Language (1) * Voice (1) * NARS * Exposure |  | -0.215 |  | 0.279 |  | 1227.796 |  | -0.769 |  | 0.442 |  |
| Language (1) * Voice (2) * NARS * Exposure |  | -0.200 |  | 0.279 |  | 1227.796 |  | -0.717 |  | 0.474 |  |
| Language (1) * Voice (1) * Interest * Exposure |  | -0.525 |  | 0.532 |  | 1227.796 |  | -0.987 |  | 0.324 |  |
| Language (1) * Voice (2) * Interest * Exposure |  | -0.272 |  | 0.532 |  | 1227.796 |  | -0.512 |  | 0.609 |  |
| Language (1) * NARS * Interest * Exposure |  | 0.082 |  | 0.237 |  | 87.761 |  | 0.345 |  | 0.731 |  |
| Voice (1) * NARS * Interest * Exposure |  | 0.151 |  | 0.143 |  | 1227.796 |  | 1.052 |  | 0.293 |  |
| Voice (2) * NARS * Interest * Exposure |  | 0.031 |  | 0.143 |  | 1227.796 |  | 0.219 |  | 0.827 |  |
| Language (1) * Voice (1) * NARS * Interest * Exposure |  | 0.132 |  | 0.143 |  | 1227.796 |  | 0.922 |  | 0.357 |  |
| Language (1) * Voice (2) * NARS * Interest * Exposure |  | 0.072 |  | 0.143 |  | 1227.796 |  | 0.500 |  | 0.617 |  |
|  | | | | | | | | | | | |
| *Note.*  The intercept corresponds to the (unweighted) grand mean; for each factor with k levels, k - 1 parameters are estimated. Consequently, the estimates cannot be directly mapped to factor levels. Significant effects are highlighted in bold. | | | | | | | | | | | |

| Supplementary Table 28  *Results of linear mixed modeling with language, voice type, robots-related factors as predictors and prosody as outcome* | | | | | | | | | | | |
| --- | --- | --- | --- | --- | --- | --- | --- | --- | --- | --- | --- |
| Factor | | Estimate | | *SE* | | *df* | | *t* | | *p* | |
| Intercept |  | 1.206 |  | 4.663 |  | 87.713 |  | 0.259 |  | 0.797 |  |
| Language (1) |  | -4.545 |  | 4.662 |  | 87.652 |  | -0.975 |  | 0.332 |  |
| Voice (1) |  | 3.750 |  | 4.778 |  | 87.551 |  | 0.785 |  | 0.435 |  |
| Voice (2) |  | -2.593 |  | 3.419 |  | 87.422 |  | -0.758 |  | 0.450 |  |
| NARS |  | 0.914 |  | 1.241 |  | 87.652 |  | 0.736 |  | 0.463 |  |
| Interest |  | 1.520 |  | 2.369 |  | 87.652 |  | 0.642 |  | 0.523 |  |
| Exposure |  | 0.870 |  | 1.450 |  | 87.652 |  | 0.600 |  | 0.550 |  |
| Language (1) * Voice (1) |  | 1.679 |  | 4.776 |  | 87.475 |  | 0.352 |  | 0.726 |  |
| Language (1) * Voice (2) |  | -2.189 |  | 3.417 |  | 87.247 |  | -0.641 |  | 0.523 |  |
| Language (1) * NARS |  | 1.194 |  | 1.241 |  | 87.652 |  | 0.962 |  | 0.339 |  |
| Voice (1) * NARS |  | -0.474 |  | 1.272 |  | 87.475 |  | -0.373 |  | 0.710 |  |
| Voice (2) * NARS |  | 0.591 |  | 0.910 |  | 87.247 |  | 0.650 |  | 0.517 |  |
| Language (1) * Interest |  | 2.121 |  | 2.369 |  | 87.652 |  | 0.895 |  | 0.373 |  |
| Voice (1) * Interest |  | -0.920 |  | 2.427 |  | 87.475 |  | -0.379 |  | 0.706 |  |
| Voice (2) * Interest |  | 0.774 |  | 1.737 |  | 87.247 |  | 0.446 |  | 0.657 |  |
| NARS * Interest |  | -0.428 |  | 0.636 |  | 87.652 |  | -0.673 |  | 0.503 |  |
| Language (1) * Exposure |  | 1.318 |  | 1.450 |  | 87.652 |  | 0.909 |  | 0.366 |  |
| Voice (1) * Exposure |  | 0.010 |  | 1.486 |  | 87.476 |  | 0.007 |  | 0.995 |  |
| Voice (2) * Exposure |  | 0.311 |  | 1.063 |  | 87.248 |  | 0.293 |  | 0.771 |  |
| NARS * Exposure |  | -0.273 |  | 0.388 |  | 87.653 |  | -0.704 |  | 0.483 |  |
| Interest * Exposure |  | -0.393 |  | 0.739 |  | 87.653 |  | -0.532 |  | 0.596 |  |
| Language (1) * Voice (1) * NARS |  | -0.509 |  | 1.272 |  | 87.475 |  | -0.400 |  | 0.690 |  |
| Language (1) * Voice (2) * NARS |  | 0.734 |  | 0.910 |  | 87.247 |  | 0.807 |  | 0.422 |  |
| Language (1) * Voice (1) * Interest |  | -1.003 |  | 2.427 |  | 87.475 |  | -0.413 |  | 0.680 |  |
| Language (1) * Voice (2) * Interest |  | 1.164 |  | 1.737 |  | 87.247 |  | 0.670 |  | 0.504 |  |
| Language (1) * NARS * Interest |  | -0.551 |  | 0.636 |  | 87.652 |  | -0.867 |  | 0.388 |  |
| Voice (1) * NARS * Interest |  | 0.226 |  | 0.651 |  | 87.476 |  | 0.347 |  | 0.729 |  |
| Voice (2) * NARS * Interest |  | -0.265 |  | 0.466 |  | 87.248 |  | -0.570 |  | 0.570 |  |
| Language (1) * Voice (1) * Exposure |  | 0.307 |  | 1.486 |  | 87.476 |  | 0.207 |  | 0.837 |  |
| Language (1) * Voice (2) * Exposure |  | 0.619 |  | 1.063 |  | 87.248 |  | 0.583 |  | 0.562 |  |
| Language (1) * NARS * Exposure |  | -0.367 |  | 0.388 |  | 87.653 |  | -0.946 |  | 0.347 |  |
| Voice (1) * NARS * Exposure |  | -0.025 |  | 0.398 |  | 87.480 |  | -0.063 |  | 0.950 |  |
| Voice (2) * NARS * Exposure |  | -0.127 |  | 0.284 |  | 87.250 |  | -0.445 |  | 0.658 |  |
| Language (1) * Interest * Exposure |  | -0.671 |  | 0.739 |  | 87.653 |  | -0.908 |  | 0.366 |  |
| Voice (1) * Interest * Exposure |  | -0.078 |  | 0.757 |  | 87.479 |  | -0.103 |  | 0.918 |  |
| Voice (2) * Interest * Exposure |  | -0.106 |  | 0.541 |  | 87.249 |  | -0.197 |  | 0.845 |  |
| NARS * Interest * Exposure |  | 0.114 |  | 0.199 |  | 87.657 |  | 0.571 |  | 0.570 |  |
| Language (1) * Voice (1) * NARS * Interest |  | 0.319 |  | 0.651 |  | 87.476 |  | 0.490 |  | 0.625 |  |
| Language (1) * Voice (2) * NARS * Interest |  | -0.387 |  | 0.466 |  | 87.248 |  | -0.831 |  | 0.408 |  |
| Language (1) * Voice (1) * NARS * Exposure |  | -0.020 |  | 0.398 |  | 87.480 |  | -0.051 |  | 0.960 |  |
| Language (1) * Voice (2) * NARS * Exposure |  | -0.223 |  | 0.284 |  | 87.250 |  | -0.784 |  | 0.435 |  |
| Language (1) * Voice (1) * Interest * Exposure |  | -0.115 |  | 0.757 |  | 87.479 |  | -0.152 |  | 0.880 |  |
| Language (1) * Voice (2) * Interest * Exposure |  | -0.287 |  | 0.541 |  | 87.249 |  | -0.529 |  | 0.598 |  |
| Language (1) * NARS * Interest * Exposure |  | 0.179 |  | 0.199 |  | 87.657 |  | 0.900 |  | 0.371 |  |
| Voice (1) * NARS * Interest * Exposure |  | 0.056 |  | 0.204 |  | 87.493 |  | 0.276 |  | 0.783 |  |
| Voice (2) * NARS * Interest * Exposure |  | 0.041 |  | 0.146 |  | 87.256 |  | 0.279 |  | 0.781 |  |
| Language (1) * Voice (1) * NARS * Interest * Exposure |  | -0.004 |  | 0.204 |  | 87.493 |  | -0.019 |  | 0.985 |  |
| Language (1) * Voice (2) * NARS * Interest * Exposure |  | 0.105 |  | 0.146 |  | 87.256 |  | 0.721 |  | 0.473 |  |
|  | | | | | | | | | | | |
| *Note.*  The intercept corresponds to the (unweighted) grand mean; for each factor with k levels, k - 1 parameters are estimated. Consequently, the estimates cannot be directly mapped to factor levels. Significant effects are highlighted in bold. | | | | | | | | | | | |

| Supplementary Table 29  *Results of linear mixed modeling with language, voice type, robots-related factors as predictors and trustworthiness as outcome* | | | | | | | | | | | |
| --- | --- | --- | --- | --- | --- | --- | --- | --- | --- | --- | --- |
| Factor | | Estimate | | *SE* | | *df* | | *t* | | *p* | |
| Intercept |  | 8.403 |  | 5.636 |  | 87.613 |  | 1.491 |  | 0.140 |  |
| Language (1) |  | 3.738 |  | 5.635 |  | 87.554 |  | 0.663 |  | 0.509 |  |
| **Voice (1)** |  | **6.990** |  | **3.546** |  | **1227.860** |  | **1.971** |  | **0.049** |  |
| Voice (2) |  | -3.578 |  | 3.546 |  | 1227.860 |  | -1.009 |  | 0.313 |  |
| NARS |  | -1.067 |  | 1.501 |  | 87.554 |  | -0.711 |  | 0.479 |  |
| Interest |  | -2.378 |  | 2.864 |  | 87.554 |  | -0.830 |  | 0.409 |  |
| Exposure |  | -0.980 |  | 1.753 |  | 87.554 |  | -0.559 |  | 0.578 |  |
| Language (1) * Voice (1) |  | 2.570 |  | 3.546 |  | 1227.860 |  | 0.725 |  | 0.469 |  |
| Language (1) * Voice (2) |  | -1.561 |  | 3.546 |  | 1227.860 |  | -0.440 |  | 0.660 |  |
| Language (1) * NARS |  | -1.159 |  | 1.501 |  | 87.554 |  | -0.773 |  | 0.442 |  |
| Voice (1) * NARS |  | -1.375 |  | 0.944 |  | 1227.860 |  | -1.456 |  | 0.146 |  |
| Voice (2) * NARS |  | 0.784 |  | 0.944 |  | 1227.860 |  | 0.830 |  | 0.407 |  |
| Language (1) * Interest |  | -1.957 |  | 2.864 |  | 87.554 |  | -0.683 |  | 0.496 |  |
| Voice (1) * Interest |  | -3.225 |  | 1.802 |  | 1227.860 |  | -1.790 |  | 0.074 |  |
| Voice (2) * Interest |  | 1.499 |  | 1.802 |  | 1227.860 |  | 0.832 |  | 0.406 |  |
| NARS * Interest |  | 0.567 |  | 0.768 |  | 87.554 |  | 0.738 |  | 0.463 |  |
| Language (1) * Exposure |  | -0.852 |  | 1.753 |  | 87.554 |  | -0.486 |  | 0.628 |  |
| Voice (1) * Exposure |  | -0.784 |  | 1.103 |  | 1227.860 |  | -0.711 |  | 0.477 |  |
| Voice (2) * Exposure |  | 1.101 |  | 1.103 |  | 1227.860 |  | 0.998 |  | 0.318 |  |
| NARS * Exposure |  | 0.221 |  | 0.469 |  | 87.554 |  | 0.472 |  | 0.638 |  |
| Interest * Exposure |  | 0.607 |  | 0.893 |  | 87.554 |  | 0.680 |  | 0.498 |  |
| Language (1) * Voice (1) * NARS |  | -0.566 |  | 0.944 |  | 1227.860 |  | -0.599 |  | 0.549 |  |
| Language (1) * Voice (2) * NARS |  | 0.533 |  | 0.944 |  | 1227.860 |  | 0.565 |  | 0.572 |  |
| Language (1) * Voice (1) * Interest |  | -1.294 |  | 1.802 |  | 1227.860 |  | -0.718 |  | 0.473 |  |
| Language (1) * Voice (2) * Interest |  | 0.775 |  | 1.802 |  | 1227.860 |  | 0.430 |  | 0.667 |  |
| Language (1) * NARS * Interest |  | 0.586 |  | 0.768 |  | 87.554 |  | 0.763 |  | 0.447 |  |
| Voice (1) * NARS * Interest |  | 0.864 |  | 0.483 |  | 1227.860 |  | 1.787 |  | 0.074 |  |
| Voice (2) * NARS * Interest |  | -0.453 |  | 0.483 |  | 1227.860 |  | -0.938 |  | 0.348 |  |
| Language (1) * Voice (1) * Exposure |  | -0.012 |  | 1.103 |  | 1227.860 |  | -0.011 |  | 0.991 |  |
| Language (1) * Voice (2) * Exposure |  | 0.885 |  | 1.103 |  | 1227.860 |  | 0.802 |  | 0.423 |  |
| Language (1) * NARS * Exposure |  | 0.244 |  | 0.469 |  | 87.554 |  | 0.520 |  | 0.604 |  |
| Voice (1) * NARS * Exposure |  | 0.182 |  | 0.295 |  | 1227.860 |  | 0.617 |  | 0.537 |  |
| Voice (2) * NARS * Exposure |  | -0.300 |  | 0.295 |  | 1227.860 |  | -1.016 |  | 0.310 |  |
| Language (1) * Interest * Exposure |  | 0.470 |  | 0.893 |  | 87.554 |  | 0.526 |  | 0.600 |  |
| Voice (1) * Interest * Exposure |  | 0.431 |  | 0.562 |  | 1227.860 |  | 0.767 |  | 0.443 |  |
| Voice (2) * Interest * Exposure |  | -0.535 |  | 0.562 |  | 1227.860 |  | -0.952 |  | 0.341 |  |
| NARS * Interest * Exposure |  | -0.143 |  | 0.241 |  | 87.554 |  | -0.595 |  | 0.553 |  |
| Language (1) * Voice (1) * NARS * Interest |  | 0.300 |  | 0.483 |  | 1227.860 |  | 0.620 |  | 0.535 |  |
| Language (1) * Voice (2) * NARS * Interest |  | -0.291 |  | 0.483 |  | 1227.860 |  | -0.602 |  | 0.547 |  |
| Language (1) * Voice (1) * NARS * Exposure |  | -0.008 |  | 0.295 |  | 1227.860 |  | -0.029 |  | 0.977 |  |
| Language (1) * Voice (2) * NARS * Exposure |  | -0.268 |  | 0.295 |  | 1227.860 |  | -0.906 |  | 0.365 |  |
| Language (1) * Voice (1) * Interest * Exposure |  | -0.055 |  | 0.562 |  | 1227.860 |  | -0.098 |  | 0.922 |  |
| Language (1) * Voice (2) * Interest * Exposure |  | -0.372 |  | 0.562 |  | 1227.860 |  | -0.662 |  | 0.508 |  |
| Language (1) * NARS * Interest * Exposure |  | -0.135 |  | 0.241 |  | 87.554 |  | -0.563 |  | 0.575 |  |
| Voice (1) * NARS * Interest * Exposure |  | -0.088 |  | 0.151 |  | 1227.860 |  | -0.582 |  | 0.561 |  |
| Voice (2) * NARS * Interest * Exposure |  | 0.145 |  | 0.151 |  | 1227.860 |  | 0.959 |  | 0.338 |  |
| Language (1) * Voice (1) * NARS * Interest * Exposure |  | 0.027 |  | 0.151 |  | 1227.860 |  | 0.177 |  | 0.859 |  |
| Language (1) * Voice (2) * NARS * Interest * Exposure |  | 0.113 |  | 0.151 |  | 1227.860 |  | 0.745 |  | 0.456 |  |
|  | | | | | | | | | | | |
| *Note.*  The intercept corresponds to the (unweighted) grand mean; for each factor with k levels, k - 1 parameters are estimated. Consequently, the estimates cannot be directly mapped to factor levels. Significant effects are highlighted in bold. | | | | | | | | | | | |

| Supplementary Table 30  *Results of linear mixed modeling with language, voice type, robots-related factors as predictors and confidence as outcome* | | | | | | | | | | | |
| --- | --- | --- | --- | --- | --- | --- | --- | --- | --- | --- | --- |
| Factor | | Estimate | | *SE* | | *df* | | *t* | | *p* | |
| Intercept |  | 2.883 |  | 5.712 |  | 87.798 |  | 0.505 |  | 0.615 |  |
| Language (1) |  | -2.220 |  | 5.711 |  | 87.730 |  | -0.389 |  | 0.698 |  |
| Voice (1) |  | 0.169 |  | 5.575 |  | 88.009 |  | 0.030 |  | 0.976 |  |
| Voice (2) |  | -4.240 |  | 3.756 |  | 87.989 |  | -1.129 |  | 0.262 |  |
| NARS |  | 0.567 |  | 1.521 |  | 87.730 |  | 0.373 |  | 0.710 |  |
| Interest |  | 0.487 |  | 2.902 |  | 87.730 |  | 0.168 |  | 0.867 |  |
| Exposure |  | 0.621 |  | 1.776 |  | 87.730 |  | 0.349 |  | 0.728 |  |
| Language (1) * Voice (1) |  | -4.120 |  | 5.575 |  | 88.009 |  | -0.739 |  | 0.462 |  |
| Language (1) * Voice (2) |  | -1.533 |  | 3.756 |  | 87.989 |  | -0.408 |  | 0.684 |  |
| Language (1) * NARS |  | 0.567 |  | 1.521 |  | 87.730 |  | 0.373 |  | 0.710 |  |
| Voice (1) * NARS |  | 0.492 |  | 1.485 |  | 88.009 |  | 0.332 |  | 0.741 |  |
| Voice (2) * NARS |  | 1.001 |  | 1.000 |  | 87.989 |  | 1.000 |  | 0.320 |  |
| Language (1) * Interest |  | 1.155 |  | 2.902 |  | 87.730 |  | 0.398 |  | 0.692 |  |
| Voice (1) * Interest |  | 0.623 |  | 2.833 |  | 88.009 |  | 0.220 |  | 0.827 |  |
| Voice (2) * Interest |  | 1.686 |  | 1.909 |  | 87.989 |  | 0.883 |  | 0.379 |  |
| NARS * Interest |  | -0.232 |  | 0.779 |  | 87.730 |  | -0.299 |  | 0.766 |  |
| Language (1) * Exposure |  | 0.980 |  | 1.776 |  | 87.730 |  | 0.552 |  | 0.582 |  |
| Voice (1) * Exposure |  | 0.749 |  | 1.734 |  | 88.009 |  | 0.432 |  | 0.667 |  |
| Voice (2) * Exposure |  | 0.489 |  | 1.168 |  | 87.989 |  | 0.418 |  | 0.677 |  |
| NARS * Exposure |  | -0.237 |  | 0.475 |  | 87.730 |  | -0.499 |  | 0.619 |  |
| Interest * Exposure |  | -0.167 |  | 0.905 |  | 87.730 |  | -0.184 |  | 0.854 |  |
| Language (1) * Voice (1) * NARS |  | 1.121 |  | 1.485 |  | 88.009 |  | 0.755 |  | 0.452 |  |
| Language (1) * Voice (2) * NARS |  | 0.587 |  | 1.000 |  | 87.989 |  | 0.587 |  | 0.558 |  |
| Language (1) * Voice (1) * Interest |  | 1.950 |  | 2.833 |  | 88.008 |  | 0.688 |  | 0.493 |  |
| Language (1) * Voice (2) * Interest |  | 0.725 |  | 1.909 |  | 87.989 |  | 0.380 |  | 0.705 |  |
| Language (1) * NARS * Interest |  | -0.292 |  | 0.779 |  | 87.730 |  | -0.375 |  | 0.709 |  |
| Voice (1) * NARS * Interest |  | -0.179 |  | 0.760 |  | 88.009 |  | -0.235 |  | 0.815 |  |
| Voice (2) * NARS * Interest |  | -0.540 |  | 0.512 |  | 87.989 |  | -1.055 |  | 0.294 |  |
| Language (1) * Voice (1) * Exposure |  | 1.614 |  | 1.734 |  | 88.008 |  | 0.931 |  | 0.354 |  |
| Language (1) * Voice (2) * Exposure |  | 0.010 |  | 1.168 |  | 87.989 |  | 0.008 |  | 0.993 |  |
| Language (1) * NARS * Exposure |  | -0.292 |  | 0.475 |  | 87.730 |  | -0.615 |  | 0.540 |  |
| Voice (1) * NARS * Exposure |  | -0.229 |  | 0.464 |  | 88.009 |  | -0.492 |  | 0.624 |  |
| Voice (2) * NARS * Exposure |  | -0.149 |  | 0.313 |  | 87.989 |  | -0.477 |  | 0.635 |  |
| Language (1) * Interest * Exposure |  | -0.516 |  | 0.905 |  | 87.730 |  | -0.570 |  | 0.570 |  |
| Voice (1) * Interest * Exposure |  | -0.443 |  | 0.883 |  | 88.008 |  | -0.502 |  | 0.617 |  |
| Voice (2) * Interest * Exposure |  | -0.177 |  | 0.595 |  | 87.989 |  | -0.298 |  | 0.766 |  |
| NARS * Interest * Exposure |  | 0.077 |  | 0.244 |  | 87.730 |  | 0.316 |  | 0.753 |  |
| Language (1) * Voice (1) * NARS * Interest |  | -0.531 |  | 0.760 |  | 88.009 |  | -0.699 |  | 0.486 |  |
| Language (1) * Voice (2) * NARS * Interest |  | -0.270 |  | 0.512 |  | 87.989 |  | -0.528 |  | 0.599 |  |
| Language (1) * Voice (1) * NARS * Exposure |  | -0.406 |  | 0.464 |  | 88.008 |  | -0.875 |  | 0.384 |  |
| Language (1) * Voice (2) * NARS * Exposure |  | -0.048 |  | 0.313 |  | 87.989 |  | -0.152 |  | 0.879 |  |
| Language (1) * Voice (1) * Interest * Exposure |  | -0.871 |  | 0.883 |  | 88.008 |  | -0.986 |  | 0.327 |  |
| Language (1) * Voice (2) * Interest * Exposure |  | 0.089 |  | 0.595 |  | 87.989 |  | 0.149 |  | 0.882 |  |
| Language (1) * NARS * Interest * Exposure |  | 0.141 |  | 0.244 |  | 87.730 |  | 0.579 |  | 0.564 |  |
| Voice (1) * NARS * Interest * Exposure |  | 0.145 |  | 0.238 |  | 88.008 |  | 0.609 |  | 0.544 |  |
| Voice (2) * NARS * Interest * Exposure |  | 0.063 |  | 0.160 |  | 87.989 |  | 0.393 |  | 0.695 |  |
| Language (1) * Voice (1) * NARS * Interest * Exposure |  | 0.232 |  | 0.238 |  | 88.008 |  | 0.973 |  | 0.333 |  |
| Language (1) * Voice (2) * NARS * Interest * Exposure |  | -0.015 |  | 0.160 |  | 87.989 |  | -0.095 |  | 0.924 |  |
|  | | | | | | | | | | | |
| *Note.*  The intercept corresponds to the (unweighted) grand mean; for each factor with k levels, k - 1 parameters are estimated. Consequently, the estimates cannot be directly mapped to factor levels. Significant effects are highlighted in bold. | | | | | | | | | | | |

| Supplementary Table 31  *Results of linear mixed modeling with language, voice type, robots-related factors as predictors and enthusiasm as outcome* | | | | | | | | | | | |
| --- | --- | --- | --- | --- | --- | --- | --- | --- | --- | --- | --- |
| Factor | | Estimate | | *SE* | | *df* | | *t* | | *p* | |
| Intercept |  | 2.781 |  | 5.942 |  | 87.841 |  | 0.468 |  | 0.641 |  |
| Language (1) |  | -1.807 |  | 5.941 |  | 87.777 |  | -0.304 |  | 0.762 |  |
| Voice (1) |  | 1.388 |  | 5.620 |  | 87.846 |  | 0.247 |  | 0.806 |  |
| Voice (2) |  | -1.241 |  | 3.949 |  | 87.742 |  | -0.314 |  | 0.754 |  |
| NARS |  | 0.546 |  | 1.582 |  | 87.777 |  | 0.345 |  | 0.731 |  |
| Interest |  | 0.588 |  | 3.019 |  | 87.777 |  | 0.195 |  | 0.846 |  |
| Exposure |  | 0.454 |  | 1.848 |  | 87.777 |  | 0.246 |  | 0.807 |  |
| Language (1) * Voice (1) |  | -2.766 |  | 5.620 |  | 87.829 |  | -0.492 |  | 0.624 |  |
| Language (1) * Voice (2) |  | -0.199 |  | 3.948 |  | 87.662 |  | -0.050 |  | 0.960 |  |
| Language (1) * NARS |  | 0.626 |  | 1.582 |  | 87.777 |  | 0.395 |  | 0.694 |  |
| Voice (1) * NARS |  | 0.129 |  | 1.497 |  | 87.829 |  | 0.086 |  | 0.932 |  |
| Voice (2) * NARS |  | 0.229 |  | 1.051 |  | 87.662 |  | 0.218 |  | 0.828 |  |
| Language (1) * Interest |  | 0.947 |  | 3.019 |  | 87.777 |  | 0.314 |  | 0.755 |  |
| Voice (1) * Interest |  | 0.132 |  | 2.856 |  | 87.829 |  | 0.046 |  | 0.963 |  |
| Voice (2) * Interest |  | 0.028 |  | 2.006 |  | 87.662 |  | 0.014 |  | 0.989 |  |
| NARS * Interest |  | -0.256 |  | 0.810 |  | 87.777 |  | -0.317 |  | 0.752 |  |
| Language (1) * Exposure |  | 0.917 |  | 1.848 |  | 87.777 |  | 0.496 |  | 0.621 |  |
| Voice (1) * Exposure |  | 0.410 |  | 1.748 |  | 87.829 |  | 0.234 |  | 0.815 |  |
| Voice (2) * Exposure |  | -0.178 |  | 1.228 |  | 87.662 |  | -0.145 |  | 0.885 |  |
| NARS * Exposure |  | -0.215 |  | 0.495 |  | 87.777 |  | -0.434 |  | 0.665 |  |
| Interest * Exposure |  | -0.077 |  | 0.941 |  | 87.777 |  | -0.082 |  | 0.935 |  |
| Language (1) * Voice (1) * NARS |  | 0.666 |  | 1.497 |  | 87.829 |  | 0.445 |  | 0.658 |  |
| Language (1) * Voice (2) * NARS |  | 0.297 |  | 1.051 |  | 87.662 |  | 0.282 |  | 0.778 |  |
| Language (1) * Voice (1) * Interest |  | 0.988 |  | 2.856 |  | 87.829 |  | 0.346 |  | 0.730 |  |
| Language (1) * Voice (2) * Interest |  | 0.084 |  | 2.006 |  | 87.662 |  | 0.042 |  | 0.967 |  |
| Language (1) * NARS * Interest |  | -0.276 |  | 0.810 |  | 87.777 |  | -0.341 |  | 0.734 |  |
| Voice (1) * NARS * Interest |  | -0.050 |  | 0.766 |  | 87.829 |  | -0.065 |  | 0.948 |  |
| Voice (2) * NARS * Interest |  | -0.143 |  | 0.538 |  | 87.662 |  | -0.266 |  | 0.791 |  |
| Language (1) * Voice (1) * Exposure |  | 1.332 |  | 1.748 |  | 87.829 |  | 0.762 |  | 0.448 |  |
| Language (1) * Voice (2) * Exposure |  | 1.378e -4 |  | 1.228 |  | 87.663 |  | 1.122e -4 |  | 1.000 |  |
| Language (1) * NARS * Exposure |  | -0.325 |  | 0.495 |  | 87.777 |  | -0.656 |  | 0.513 |  |
| Voice (1) * NARS * Exposure |  | -0.117 |  | 0.468 |  | 87.829 |  | -0.250 |  | 0.803 |  |
| Voice (2) * NARS * Exposure |  | 0.001 |  | 0.329 |  | 87.662 |  | 0.003 |  | 0.997 |  |
| Language (1) * Interest * Exposure |  | -0.474 |  | 0.941 |  | 87.777 |  | -0.503 |  | 0.616 |  |
| Voice (1) * Interest * Exposure |  | -0.327 |  | 0.890 |  | 87.829 |  | -0.367 |  | 0.715 |  |
| Voice (2) * Interest * Exposure |  | 0.156 |  | 0.625 |  | 87.662 |  | 0.249 |  | 0.804 |  |
| NARS * Interest * Exposure |  | 0.059 |  | 0.254 |  | 87.777 |  | 0.233 |  | 0.817 |  |
| Language (1) * Voice (1) * NARS * Interest |  | -0.211 |  | 0.766 |  | 87.829 |  | -0.276 |  | 0.783 |  |
| Language (1) * Voice (2) * NARS * Interest |  | -0.153 |  | 0.538 |  | 87.662 |  | -0.284 |  | 0.777 |  |
| Language (1) * Voice (1) * NARS * Exposure |  | -0.294 |  | 0.468 |  | 87.829 |  | -0.628 |  | 0.532 |  |
| Language (1) * Voice (2) * NARS * Exposure |  | -0.076 |  | 0.329 |  | 87.663 |  | -0.231 |  | 0.818 |  |
| Language (1) * Voice (1) * Interest * Exposure |  | -0.601 |  | 0.890 |  | 87.829 |  | -0.675 |  | 0.501 |  |
| Language (1) * Voice (2) * Interest * Exposure |  | 0.062 |  | 0.625 |  | 87.662 |  | 0.099 |  | 0.921 |  |
| Language (1) * NARS * Interest * Exposure |  | 0.150 |  | 0.254 |  | 87.777 |  | 0.591 |  | 0.556 |  |
| Voice (1) * NARS * Interest * Exposure |  | 0.111 |  | 0.240 |  | 87.829 |  | 0.461 |  | 0.646 |  |
| Voice (2) * NARS * Interest * Exposure |  | -0.011 |  | 0.169 |  | 87.662 |  | -0.064 |  | 0.949 |  |
| Language (1) * Voice (1) * NARS * Interest * Exposure |  | 0.129 |  | 0.240 |  | 87.829 |  | 0.539 |  | 0.591 |  |
| Language (1) * Voice (2) * NARS * Interest * Exposure |  | 0.017 |  | 0.169 |  | 87.662 |  | 0.104 |  | 0.918 |  |
|  | | | | | | | | | | | |
| *Note.*  The intercept corresponds to the (unweighted) grand mean; for each factor with k levels, k - 1 parameters are estimated. Consequently, the estimates cannot be directly mapped to factor levels. Significant effects are highlighted in bold. | | | | | | | | | | | |

| Supplementary Table 32  *Results of linear mixed modeling with language, voice type, robots-related factors as predictors and pleasantness as outcome* | | | | | | | | | | | |
| --- | --- | --- | --- | --- | --- | --- | --- | --- | --- | --- | --- |
| Factor | | Estimate | | *SE* | | *df* | | *t* | | *p* | |
| Intercept |  | 14.867 |  | 5.711 |  | 87.712 |  | 2.603 |  | 0.011 |  |
| Language (1) |  | 8.838 |  | 5.710 |  | 87.656 |  | 1.548 |  | 0.125 |  |
| Voice (1) |  | 1.202 |  | 5.316 |  | 87.999 |  | 0.226 |  | 0.822 |  |
| Voice (2) |  | -0.433 |  | 4.354 |  | 88.000 |  | -0.099 |  | 0.921 |  |
| NARS |  | -2.816 |  | 1.521 |  | 87.656 |  | -1.852 |  | 0.067 |  |
| Interest |  | -5.604 |  | 2.902 |  | 87.656 |  | -1.931 |  | 0.057 |  |
| Exposure |  | -3.073 |  | 1.776 |  | 87.656 |  | -1.730 |  | 0.087 |  |
| Language (1) * Voice (1) |  | -2.022 |  | 5.316 |  | 87.999 |  | -0.380 |  | 0.705 |  |
| Language (1) * Voice (2) |  | 0.857 |  | 4.354 |  | 88.000 |  | 0.197 |  | 0.844 |  |
| Language (1) * NARS |  | -2.436 |  | 1.521 |  | 87.656 |  | -1.602 |  | 0.113 |  |
| Voice (1) * NARS |  | 0.130 |  | 1.416 |  | 87.999 |  | 0.092 |  | 0.927 |  |
| Voice (2) * NARS |  | 0.014 |  | 1.160 |  | 88.000 |  | 0.012 |  | 0.991 |  |
| Language (1) * Interest |  | -4.871 |  | 2.902 |  | 87.656 |  | -1.679 |  | 0.097 |  |
| Voice (1) * Interest |  | -0.360 |  | 2.701 |  | 87.999 |  | -0.133 |  | 0.894 |  |
| Voice (2) * Interest |  | 0.114 |  | 2.213 |  | 88.000 |  | 0.052 |  | 0.959 |  |
| NARS * Interest |  | 1.483 |  | 0.778 |  | 87.656 |  | 1.906 |  | 0.060 |  |
| Language (1) * Exposure |  | -2.311 |  | 1.776 |  | 87.656 |  | -1.301 |  | 0.197 |  |
| Voice (1) * Exposure |  | 0.571 |  | 1.654 |  | 87.999 |  | 0.346 |  | 0.731 |  |
| Voice (2) * Exposure |  | -0.297 |  | 1.355 |  | 88.000 |  | -0.219 |  | 0.827 |  |
| NARS * Exposure |  | 0.777 |  | 0.475 |  | 87.656 |  | 1.634 |  | 0.106 |  |
| Interest * Exposure |  | 1.674 |  | 0.905 |  | 87.656 |  | 1.851 |  | 0.068 |  |
| Language (1) * Voice (1) * NARS |  | 0.656 |  | 1.416 |  | 87.999 |  | 0.463 |  | 0.644 |  |
| Language (1) * Voice (2) * NARS |  | -0.064 |  | 1.160 |  | 88.000 |  | -0.055 |  | 0.956 |  |
| Language (1) * Voice (1) * Interest |  | 1.026 |  | 2.701 |  | 87.999 |  | 0.380 |  | 0.705 |  |
| Language (1) * Voice (2) * Interest |  | -0.446 |  | 2.213 |  | 88.000 |  | -0.202 |  | 0.841 |  |
| Language (1) * NARS * Interest |  | 1.329 |  | 0.778 |  | 87.656 |  | 1.707 |  | 0.091 |  |
| Voice (1) * NARS * Interest |  | 0.058 |  | 0.725 |  | 87.999 |  | 0.080 |  | 0.936 |  |
| Voice (2) * NARS * Interest |  | -0.095 |  | 0.594 |  | 88.000 |  | -0.160 |  | 0.873 |  |
| Language (1) * Voice (1) * Exposure |  | 0.951 |  | 1.654 |  | 87.999 |  | 0.575 |  | 0.567 |  |
| Language (1) * Voice (2) * Exposure |  | -0.313 |  | 1.355 |  | 88.000 |  | -0.231 |  | 0.818 |  |
| Language (1) * NARS * Exposure |  | 0.623 |  | 0.475 |  | 87.656 |  | 1.310 |  | 0.194 |  |
| Voice (1) * NARS * Exposure |  | -0.158 |  | 0.443 |  | 87.999 |  | -0.357 |  | 0.722 |  |
| Voice (2) * NARS * Exposure |  | 0.033 |  | 0.363 |  | 88.000 |  | 0.091 |  | 0.928 |  |
| Language (1) * Interest * Exposure |  | 1.215 |  | 0.905 |  | 87.656 |  | 1.344 |  | 0.183 |  |
| Voice (1) * Interest * Exposure |  | -0.227 |  | 0.842 |  | 87.999 |  | -0.269 |  | 0.788 |  |
| Voice (2) * Interest * Exposure |  | 0.120 |  | 0.690 |  | 88.000 |  | 0.174 |  | 0.862 |  |
| NARS * Interest * Exposure |  | -0.433 |  | 0.244 |  | 87.656 |  | -1.776 |  | 0.079 |  |
| Language (1) * Voice (1) * NARS * Interest |  | -0.371 |  | 0.725 |  | 87.999 |  | -0.513 |  | 0.610 |  |
| Language (1) * Voice (2) * NARS * Interest |  | 0.048 |  | 0.594 |  | 88.000 |  | 0.081 |  | 0.936 |  |
| Language (1) * Voice (1) * NARS * Exposure |  | -0.257 |  | 0.443 |  | 87.999 |  | -0.581 |  | 0.563 |  |
| Language (1) * Voice (2) * NARS * Exposure |  | 0.026 |  | 0.363 |  | 88.000 |  | 0.071 |  | 0.943 |  |
| Language (1) * Voice (1) * Interest * Exposure |  | -0.531 |  | 0.842 |  | 87.999 |  | -0.631 |  | 0.530 |  |
| Language (1) * Voice (2) * Interest * Exposure |  | 0.241 |  | 0.690 |  | 88.000 |  | 0.349 |  | 0.728 |  |
| Language (1) * NARS * Interest * Exposure |  | -0.333 |  | 0.244 |  | 87.656 |  | -1.364 |  | 0.176 |  |
| Voice (1) * NARS * Interest * Exposure |  | 0.086 |  | 0.227 |  | 87.999 |  | 0.381 |  | 0.704 |  |
| Voice (2) * NARS * Interest * Exposure |  | -0.016 |  | 0.186 |  | 88.000 |  | -0.086 |  | 0.932 |  |
| Language (1) * Voice (1) * NARS * Interest * Exposure |  | 0.157 |  | 0.227 |  | 87.999 |  | 0.694 |  | 0.490 |  |
| Language (1) * Voice (2) * NARS * Interest * Exposure |  | -0.045 |  | 0.186 |  | 88.000 |  | -0.243 |  | 0.809 |  |
|  | | | | | | | | | | | |
| *Note.*  The intercept corresponds to the (unweighted) grand mean; for each factor with k levels, k - 1 parameters are estimated. Consequently, the estimates cannot be directly mapped to factor levels. Significant effects are highlighted in bold. | | | | | | | | | | | |

| Supplementary Table 33  *Results of linear mixed modeling with language, voice type, robots-related factors as predictors and naturalness as outcome* | | | | | | | | | | | |
| --- | --- | --- | --- | --- | --- | --- | --- | --- | --- | --- | --- |
| Factor | | Estimate | | *SE* | | *df* | | *t* | | *p* | |
| Intercept |  | 9.643 |  | 4.848 |  | 87.799 |  | 1.989 |  | 0.050 |  |
| Language (1) |  | 3.965 |  | 4.848 |  | 87.751 |  | 0.818 |  | 0.416 |  |
| Voice (1) |  | 3.237 |  | 5.639 |  | 87.997 |  | 0.574 |  | 0.567 |  |
| Voice (2) |  | -0.785 |  | 4.461 |  | 88.000 |  | -0.176 |  | 0.861 |  |
| NARS |  | -1.412 |  | 1.291 |  | 87.751 |  | -1.094 |  | 0.277 |  |
| Interest |  | -3.107 |  | 2.464 |  | 87.751 |  | -1.261 |  | 0.211 |  |
| Exposure |  | -2.038 |  | 1.508 |  | 87.751 |  | -1.352 |  | 0.180 |  |
| Language (1) * Voice (1) |  | 0.933 |  | 5.639 |  | 87.997 |  | 0.166 |  | 0.869 |  |
| Language (1) * Voice (2) |  | -0.115 |  | 4.461 |  | 88.000 |  | -0.026 |  | 0.980 |  |
| Language (1) * NARS |  | -1.138 |  | 1.291 |  | 87.751 |  | -0.882 |  | 0.380 |  |
| Voice (1) * NARS |  | -0.178 |  | 1.502 |  | 87.997 |  | -0.119 |  | 0.906 |  |
| Voice (2) * NARS |  | 0.090 |  | 1.188 |  | 88.000 |  | 0.076 |  | 0.940 |  |
| Language (1) * Interest |  | -2.511 |  | 2.464 |  | 87.751 |  | -1.019 |  | 0.311 |  |
| Voice (1) * Interest |  | 0.145 |  | 2.866 |  | 87.997 |  | 0.050 |  | 0.960 |  |
| Voice (2) * Interest |  | -0.403 |  | 2.267 |  | 88.000 |  | -0.178 |  | 0.859 |  |
| NARS * Interest |  | 0.748 |  | 0.661 |  | 87.751 |  | 1.131 |  | 0.261 |  |
| Language (1) * Exposure |  | -1.176 |  | 1.508 |  | 87.751 |  | -0.780 |  | 0.438 |  |
| Voice (1) * Exposure |  | 0.462 |  | 1.754 |  | 87.998 |  | 0.264 |  | 0.793 |  |
| Voice (2) * Exposure |  | -0.457 |  | 1.388 |  | 88.001 |  | -0.329 |  | 0.743 |  |
| NARS * Exposure |  | 0.497 |  | 0.404 |  | 87.751 |  | 1.232 |  | 0.221 |  |
| Interest * Exposure |  | 1.103 |  | 0.768 |  | 87.751 |  | 1.436 |  | 0.155 |  |
| Language (1) * Voice (1) * NARS |  | -0.205 |  | 1.502 |  | 87.997 |  | -0.136 |  | 0.892 |  |
| Language (1) * Voice (2) * NARS |  | 0.168 |  | 1.188 |  | 88.000 |  | 0.141 |  | 0.888 |  |
| Language (1) * Voice (1) * Interest |  | 0.077 |  | 2.866 |  | 87.997 |  | 0.027 |  | 0.979 |  |
| Language (1) * Voice (2) * Interest |  | -0.137 |  | 2.267 |  | 88.000 |  | -0.060 |  | 0.952 |  |
| Language (1) * NARS * Interest |  | 0.682 |  | 0.661 |  | 87.751 |  | 1.032 |  | 0.305 |  |
| Voice (1) * NARS * Interest |  | -0.057 |  | 0.769 |  | 87.997 |  | -0.074 |  | 0.941 |  |
| Voice (2) * NARS * Interest |  | 0.023 |  | 0.608 |  | 88.000 |  | 0.037 |  | 0.970 |  |
| Language (1) * Voice (1) * Exposure |  | 0.272 |  | 1.754 |  | 87.998 |  | 0.155 |  | 0.877 |  |
| Language (1) * Voice (2) * Exposure |  | -0.052 |  | 1.388 |  | 88.001 |  | -0.038 |  | 0.970 |  |
| Language (1) * NARS * Exposure |  | 0.328 |  | 0.404 |  | 87.751 |  | 0.813 |  | 0.419 |  |
| Voice (1) * NARS * Exposure |  | -0.164 |  | 0.469 |  | 87.997 |  | -0.350 |  | 0.727 |  |
| Voice (2) * NARS * Exposure |  | 0.064 |  | 0.371 |  | 88.001 |  | 0.172 |  | 0.864 |  |
| Language (1) * Interest * Exposure |  | 0.708 |  | 0.768 |  | 87.751 |  | 0.922 |  | 0.359 |  |
| Voice (1) * Interest * Exposure |  | -0.454 |  | 0.893 |  | 87.997 |  | -0.509 |  | 0.612 |  |
| Voice (2) * Interest * Exposure |  | 0.322 |  | 0.707 |  | 88.001 |  | 0.456 |  | 0.649 |  |
| NARS * Interest * Exposure |  | -0.271 |  | 0.207 |  | 87.751 |  | -1.311 |  | 0.193 |  |
| Language (1) * Voice (1) * NARS * Interest |  | 0.007 |  | 0.769 |  | 87.997 |  | 0.009 |  | 0.993 |  |
| Language (1) * Voice (2) * NARS * Interest |  | -0.049 |  | 0.608 |  | 88.000 |  | -0.080 |  | 0.936 |  |
| Language (1) * Voice (1) * NARS * Exposure |  | -0.056 |  | 0.469 |  | 87.997 |  | -0.119 |  | 0.905 |  |
| Language (1) * Voice (2) * NARS * Exposure |  | -0.039 |  | 0.371 |  | 88.001 |  | -0.104 |  | 0.918 |  |
| Language (1) * Voice (1) * Interest * Exposure |  | -0.314 |  | 0.893 |  | 87.997 |  | -0.352 |  | 0.726 |  |
| Language (1) * Voice (2) * Interest * Exposure |  | 0.152 |  | 0.707 |  | 88.001 |  | 0.214 |  | 0.831 |  |
| Language (1) * NARS * Interest * Exposure |  | -0.199 |  | 0.207 |  | 87.751 |  | -0.961 |  | 0.339 |  |
| Voice (1) * NARS * Interest * Exposure |  | 0.155 |  | 0.241 |  | 87.997 |  | 0.645 |  | 0.520 |  |
| Voice (2) * NARS * Interest * Exposure |  | -0.060 |  | 0.190 |  | 88.001 |  | -0.313 |  | 0.755 |  |
| Language (1) * Voice (1) * NARS * Interest * Exposure |  | 0.066 |  | 0.241 |  | 87.997 |  | 0.274 |  | 0.785 |  |
| Language (1) * Voice (2) * NARS * Interest * Exposure |  | -0.014 |  | 0.190 |  | 88.000 |  | -0.074 |  | 0.942 |  |
|  | | | | | | | | | | | |
| *Note.*  The intercept corresponds to the (unweighted) grand mean; for each factor with k levels, k - 1 parameters are estimated. Consequently, the estimates cannot be directly mapped to factor levels. Significant effects are highlighted in bold. | | | | | | | | | | | |

| Supplementary Table 34  *Results of linear mixed modeling with language, voice type, robots-related factors as predictors and human-likeness (voice) as outcome* | | | | | | | | | | | |
| --- | --- | --- | --- | --- | --- | --- | --- | --- | --- | --- | --- |
| Factor | | Estimate | | *SE* | | *df* | | *t* | | *p* | |
| Intercept |  | 9.196 |  | 5.012 |  | 87.878 |  | 1.835 |  | 0.070 |  |
| Language (1) |  | 3.531 |  | 5.012 |  | 87.829 |  | 0.705 |  | 0.483 |  |
| Voice (1) |  | -3.625 |  | 5.675 |  | 87.995 |  | -0.639 |  | 0.525 |  |
| Voice (2) |  | 2.509 |  | 4.248 |  | 87.978 |  | 0.591 |  | 0.556 |  |
| NARS |  | -1.144 |  | 1.335 |  | 87.829 |  | -0.857 |  | 0.394 |  |
| Interest |  | -2.947 |  | 2.547 |  | 87.830 |  | -1.157 |  | 0.250 |  |
| Exposure |  | -1.572 |  | 1.559 |  | 87.828 |  | -1.008 |  | 0.316 |  |
| Language (1) * Voice (1) |  | -4.782 |  | 5.675 |  | 87.995 |  | -0.843 |  | 0.402 |  |
| Language (1) * Voice (2) |  | 3.520 |  | 4.248 |  | 87.978 |  | 0.829 |  | 0.409 |  |
| Language (1) * NARS |  | -0.956 |  | 1.335 |  | 87.829 |  | -0.716 |  | 0.476 |  |
| Voice (1) * NARS |  | 1.522 |  | 1.511 |  | 87.995 |  | 1.007 |  | 0.317 |  |
| Voice (2) * NARS |  | -0.804 |  | 1.131 |  | 87.978 |  | -0.710 |  | 0.479 |  |
| Language (1) * Interest |  | -2.327 |  | 2.547 |  | 87.829 |  | -0.914 |  | 0.363 |  |
| Voice (1) * Interest |  | 3.750 |  | 2.884 |  | 87.995 |  | 1.300 |  | 0.197 |  |
| Voice (2) * Interest |  | -2.097 |  | 2.159 |  | 87.978 |  | -0.972 |  | 0.334 |  |
| NARS * Interest |  | 0.646 |  | 0.683 |  | 87.829 |  | 0.945 |  | 0.347 |  |
| Language (1) * Exposure |  | -0.762 |  | 1.559 |  | 87.828 |  | -0.489 |  | 0.626 |  |
| Voice (1) * Exposure |  | 2.785 |  | 1.765 |  | 87.991 |  | 1.578 |  | 0.118 |  |
| Voice (2) * Exposure |  | -1.127 |  | 1.321 |  | 87.975 |  | -0.853 |  | 0.396 |  |
| NARS * Exposure |  | 0.335 |  | 0.417 |  | 87.828 |  | 0.804 |  | 0.424 |  |
| Interest * Exposure |  | 0.844 |  | 0.794 |  | 87.828 |  | 1.063 |  | 0.291 |  |
| Language (1) * Voice (1) * NARS |  | 1.271 |  | 1.511 |  | 87.995 |  | 0.841 |  | 0.403 |  |
| Language (1) * Voice (2) * NARS |  | -0.801 |  | 1.131 |  | 87.978 |  | -0.708 |  | 0.481 |  |
| Language (1) * Voice (1) * Interest |  | 2.921 |  | 2.884 |  | 87.995 |  | 1.013 |  | 0.314 |  |
| Language (1) * Voice (2) * Interest |  | -2.031 |  | 2.159 |  | 87.978 |  | -0.941 |  | 0.349 |  |
| Language (1) * NARS * Interest |  | 0.619 |  | 0.683 |  | 87.829 |  | 0.906 |  | 0.367 |  |
| Voice (1) * NARS * Interest |  | -0.944 |  | 0.774 |  | 87.995 |  | -1.220 |  | 0.226 |  |
| Voice (2) * NARS * Interest |  | 0.459 |  | 0.579 |  | 87.978 |  | 0.793 |  | 0.430 |  |
| Language (1) * Voice (1) * Exposure |  | 2.034 |  | 1.765 |  | 87.991 |  | 1.152 |  | 0.252 |  |
| Language (1) * Voice (2) * Exposure |  | -0.929 |  | 1.321 |  | 87.976 |  | -0.703 |  | 0.484 |  |
| Language (1) * NARS * Exposure |  | 0.195 |  | 0.417 |  | 87.828 |  | 0.468 |  | 0.641 |  |
| Voice (1) * NARS * Exposure |  | -0.734 |  | 0.472 |  | 87.991 |  | -1.554 |  | 0.124 |  |
| Voice (2) * NARS * Exposure |  | 0.263 |  | 0.354 |  | 87.975 |  | 0.743 |  | 0.460 |  |
| Language (1) * Interest * Exposure |  | 0.471 |  | 0.794 |  | 87.828 |  | 0.594 |  | 0.554 |  |
| Voice (1) * Interest * Exposure |  | -1.596 |  | 0.899 |  | 87.991 |  | -1.776 |  | 0.079 |  |
| Voice (2) * Interest * Exposure |  | 0.673 |  | 0.673 |  | 87.976 |  | 1.001 |  | 0.320 |  |
| NARS * Interest * Exposure |  | -0.182 |  | 0.214 |  | 87.828 |  | -0.848 |  | 0.399 |  |
| Language (1) * Voice (1) * NARS * Interest |  | -0.748 |  | 0.774 |  | 87.995 |  | -0.967 |  | 0.336 |  |
| Language (1) * Voice (2) * NARS * Interest |  | 0.434 |  | 0.579 |  | 87.979 |  | 0.750 |  | 0.455 |  |
| Language (1) * Voice (1) * NARS * Exposure |  | -0.520 |  | 0.472 |  | 87.991 |  | -1.102 |  | 0.274 |  |
| Language (1) * Voice (2) * NARS * Exposure |  | 0.212 |  | 0.354 |  | 87.975 |  | 0.600 |  | 0.550 |  |
| Language (1) * Voice (1) * Interest * Exposure |  | -1.116 |  | 0.899 |  | 87.991 |  | -1.241 |  | 0.218 |  |
| Language (1) * Voice (2) * Interest * Exposure |  | 0.581 |  | 0.673 |  | 87.976 |  | 0.864 |  | 0.390 |  |
| Language (1) * NARS * Interest * Exposure |  | -0.127 |  | 0.214 |  | 87.828 |  | -0.596 |  | 0.553 |  |
| Voice (1) * NARS * Interest * Exposure |  | 0.435 |  | 0.242 |  | 87.991 |  | 1.794 |  | 0.076 |  |
| Voice (2) * NARS * Interest * Exposure |  | -0.161 |  | 0.181 |  | 87.976 |  | -0.890 |  | 0.376 |  |
| Language (1) * Voice (1) * NARS * Interest * Exposure |  | 0.281 |  | 0.242 |  | 87.991 |  | 1.162 |  | 0.249 |  |
| Language (1) * Voice (2) * NARS * Interest * Exposure |  | -0.134 |  | 0.181 |  | 87.976 |  | -0.739 |  | 0.462 |  |
|  | | | | | | | | | | | |
| *Note.*  The intercept corresponds to the (unweighted) grand mean; for each factor with k levels, k - 1 parameters are estimated. Consequently, the estimates cannot be directly mapped to factor levels. Significant effects are highlighted in bold. | | | | | | | | | | | |

| Supplementary Table 35  *Results of linear mixed modeling with language, voice type, robots-related factors as predictors and likability as outcome* | | | | | | | | | | | |
| --- | --- | --- | --- | --- | --- | --- | --- | --- | --- | --- | --- |
| Factor | | Estimate | | *SE* | | *df* | | *t* | | *p* | |
| Intercept |  | 11.944 |  | 5.993 |  | 87.772 |  | 1.993 |  | 0.049 |  |
| Language (1) |  | 6.173 |  | 5.992 |  | 87.736 |  | 1.030 |  | 0.306 |  |
| Voice (1) |  | 6.406 |  | 5.947 |  | 87.994 |  | 1.077 |  | 0.284 |  |
| Voice (2) |  | -4.045 |  | 4.686 |  | 88.001 |  | -0.863 |  | 0.390 |  |
| NARS |  | -2.108 |  | 1.596 |  | 87.736 |  | -1.321 |  | 0.190 |  |
| Interest |  | -4.274 |  | 3.045 |  | 87.736 |  | -1.404 |  | 0.164 |  |
| Exposure |  | -1.865 |  | 1.864 |  | 87.736 |  | -1.001 |  | 0.320 |  |
| Language (1) * Voice (1) |  | 2.133 |  | 5.947 |  | 87.994 |  | 0.359 |  | 0.721 |  |
| Language (1) * Voice (2) |  | -1.860 |  | 4.686 |  | 88.001 |  | -0.397 |  | 0.692 |  |
| Language (1) * NARS |  | -1.794 |  | 1.596 |  | 87.736 |  | -1.124 |  | 0.264 |  |
| Voice (1) * NARS |  | -1.284 |  | 1.584 |  | 87.994 |  | -0.811 |  | 0.420 |  |
| Voice (2) * NARS |  | 0.970 |  | 1.248 |  | 88.001 |  | 0.778 |  | 0.439 |  |
| Language (1) * Interest |  | -3.571 |  | 3.045 |  | 87.736 |  | -1.173 |  | 0.244 |  |
| Voice (1) * Interest |  | -2.867 |  | 3.022 |  | 87.994 |  | -0.948 |  | 0.345 |  |
| Voice (2) * Interest |  | 1.981 |  | 2.381 |  | 88.001 |  | 0.832 |  | 0.408 |  |
| NARS * Interest |  | 1.130 |  | 0.817 |  | 87.736 |  | 1.383 |  | 0.170 |  |
| Language (1) * Exposure |  | -1.139 |  | 1.864 |  | 87.736 |  | -0.611 |  | 0.543 |  |
| Voice (1) * Exposure |  | -0.722 |  | 1.850 |  | 87.994 |  | -0.390 |  | 0.697 |  |
| Voice (2) * Exposure |  | 1.508 |  | 1.458 |  | 88.001 |  | 1.034 |  | 0.304 |  |
| NARS * Exposure |  | 0.448 |  | 0.499 |  | 87.736 |  | 0.899 |  | 0.371 |  |
| Interest * Exposure |  | 1.062 |  | 0.949 |  | 87.736 |  | 1.119 |  | 0.266 |  |
| Language (1) * Voice (1) * NARS |  | -0.447 |  | 1.584 |  | 87.994 |  | -0.283 |  | 0.778 |  |
| Language (1) * Voice (2) * NARS |  | 0.688 |  | 1.248 |  | 88.001 |  | 0.551 |  | 0.583 |  |
| Language (1) * Voice (1) * Interest |  | -1.061 |  | 3.022 |  | 87.994 |  | -0.351 |  | 0.726 |  |
| Language (1) * Voice (2) * Interest |  | 1.031 |  | 2.381 |  | 88.001 |  | 0.433 |  | 0.666 |  |
| Language (1) * NARS * Interest |  | 1.016 |  | 0.817 |  | 87.736 |  | 1.243 |  | 0.217 |  |
| Voice (1) * NARS * Interest |  | 0.789 |  | 0.811 |  | 87.994 |  | 0.973 |  | 0.333 |  |
| Voice (2) * NARS * Interest |  | -0.607 |  | 0.639 |  | 88.001 |  | -0.950 |  | 0.345 |  |
| Language (1) * Voice (1) * Exposure |  | -0.148 |  | 1.850 |  | 87.994 |  | -0.080 |  | 0.936 |  |
| Language (1) * Voice (2) * Exposure |  | 1.355 |  | 1.458 |  | 88.001 |  | 0.930 |  | 0.355 |  |
| Language (1) * NARS * Exposure |  | 0.316 |  | 0.499 |  | 87.736 |  | 0.634 |  | 0.528 |  |
| Voice (1) * NARS * Exposure |  | 0.190 |  | 0.495 |  | 87.994 |  | 0.383 |  | 0.703 |  |
| Voice (2) * NARS * Exposure |  | -0.457 |  | 0.390 |  | 88.001 |  | -1.173 |  | 0.244 |  |
| Language (1) * Interest * Exposure |  | 0.673 |  | 0.949 |  | 87.736 |  | 0.709 |  | 0.480 |  |
| Voice (1) * Interest * Exposure |  | 0.408 |  | 0.942 |  | 87.994 |  | 0.433 |  | 0.666 |  |
| Voice (2) * Interest * Exposure |  | -0.850 |  | 0.742 |  | 88.001 |  | -1.145 |  | 0.255 |  |
| NARS * Interest * Exposure |  | -0.267 |  | 0.256 |  | 87.735 |  | -1.044 |  | 0.299 |  |
| Language (1) * Voice (1) * NARS * Interest |  | 0.214 |  | 0.811 |  | 87.994 |  | 0.264 |  | 0.793 |  |
| Language (1) * Voice (2) * NARS * Interest |  | -0.394 |  | 0.639 |  | 88.001 |  | -0.617 |  | 0.539 |  |
| Language (1) * Voice (1) * NARS * Exposure |  | 0.021 |  | 0.495 |  | 87.994 |  | 0.043 |  | 0.966 |  |
| Language (1) * Voice (2) * NARS * Exposure |  | -0.428 |  | 0.390 |  | 88.001 |  | -1.097 |  | 0.276 |  |
| Language (1) * Voice (1) * Interest * Exposure |  | 0.113 |  | 0.942 |  | 87.994 |  | 0.120 |  | 0.905 |  |
| Language (1) * Voice (2) * Interest * Exposure |  | -0.708 |  | 0.742 |  | 88.001 |  | -0.954 |  | 0.343 |  |
| Language (1) * NARS * Interest * Exposure |  | -0.189 |  | 0.256 |  | 87.735 |  | -0.739 |  | 0.462 |  |
| Voice (1) * NARS * Interest * Exposure |  | -0.094 |  | 0.254 |  | 87.994 |  | -0.369 |  | 0.713 |  |
| Voice (2) * NARS * Interest * Exposure |  | 0.254 |  | 0.200 |  | 88.001 |  | 1.271 |  | 0.207 |  |
| Language (1) * Voice (1) * NARS * Interest * Exposure |  | -0.011 |  | 0.254 |  | 87.994 |  | -0.045 |  | 0.965 |  |
| Language (1) * Voice (2) * NARS * Interest * Exposure |  | 0.219 |  | 0.200 |  | 88.001 |  | 1.095 |  | 0.276 |  |
|  | | | | | | | | | | | |
| *Note.*  The intercept corresponds to the (unweighted) grand mean; for each factor with k levels, k - 1 parameters are estimated. Consequently, the estimates cannot be directly mapped to factor levels. Significant effects are highlighted in bold. | | | | | | | | | | | |

| Supplementary Table 36  *Results of linear mixed modeling with language, voice type, robots-related factors as predictors and appeal as outcome* | | | | | | | | | | | |
| --- | --- | --- | --- | --- | --- | --- | --- | --- | --- | --- | --- |
| Factor | | Estimate | | *SE* | | *df* | | *t* | | *p* | |
| Intercept |  | 11.409 |  | 5.911 |  | 87.818 |  | 1.930 |  | 0.057 |  |
| Language (1) |  | 5.848 |  | 5.911 |  | 87.792 |  | 0.989 |  | 0.325 |  |
| Voice (1) |  | 2.528 |  | 5.508 |  | 88.001 |  | 0.459 |  | 0.647 |  |
| Voice (2) |  | -0.603 |  | 4.002 |  | 87.997 |  | -0.151 |  | 0.881 |  |
| NARS |  | -1.926 |  | 1.574 |  | 87.792 |  | -1.223 |  | 0.224 |  |
| Interest |  | -3.995 |  | 3.004 |  | 87.792 |  | -1.330 |  | 0.187 |  |
| Exposure |  | -1.531 |  | 1.839 |  | 87.792 |  | -0.833 |  | 0.407 |  |
| Language (1) * Voice (1) |  | 0.787 |  | 5.508 |  | 88.001 |  | 0.143 |  | 0.887 |  |
| Language (1) * Voice (2) |  | 0.818 |  | 4.002 |  | 87.997 |  | 0.204 |  | 0.839 |  |
| Language (1) * NARS |  | -1.671 |  | 1.574 |  | 87.792 |  | -1.062 |  | 0.291 |  |
| Voice (1) * NARS |  | -0.187 |  | 1.467 |  | 88.001 |  | -0.127 |  | 0.899 |  |
| Voice (2) * NARS |  | -0.003 |  | 1.066 |  | 87.997 |  | -0.003 |  | 0.998 |  |
| Language (1) * Interest |  | -3.216 |  | 3.004 |  | 87.792 |  | -1.071 |  | 0.287 |  |
| Voice (1) * Interest |  | -0.923 |  | 2.799 |  | 88.001 |  | -0.330 |  | 0.742 |  |
| Voice (2) * Interest |  | -0.031 |  | 2.034 |  | 87.997 |  | -0.015 |  | 0.988 |  |
| NARS * Interest |  | 1.030 |  | 0.806 |  | 87.792 |  | 1.278 |  | 0.205 |  |
| Language (1) * Exposure |  | -0.779 |  | 1.839 |  | 87.792 |  | -0.424 |  | 0.673 |  |
| Voice (1) * Exposure |  | -0.065 |  | 1.713 |  | 88.001 |  | -0.038 |  | 0.970 |  |
| Voice (2) * Exposure |  | 0.097 |  | 1.245 |  | 87.997 |  | 0.078 |  | 0.938 |  |
| NARS * Exposure |  | 0.345 |  | 0.492 |  | 87.792 |  | 0.701 |  | 0.485 |  |
| Interest * Exposure |  | 0.899 |  | 0.936 |  | 87.792 |  | 0.960 |  | 0.340 |  |
| Language (1) * Voice (1) * NARS |  | -0.026 |  | 1.467 |  | 88.001 |  | -0.018 |  | 0.986 |  |
| Language (1) * Voice (2) * NARS |  | -0.117 |  | 1.066 |  | 87.997 |  | -0.110 |  | 0.913 |  |
| Language (1) * Voice (1) * Interest |  | -0.329 |  | 2.799 |  | 88.001 |  | -0.118 |  | 0.907 |  |
| Language (1) * Voice (2) * Interest |  | -0.367 |  | 2.034 |  | 87.997 |  | -0.180 |  | 0.857 |  |
| Language (1) * NARS * Interest |  | 0.915 |  | 0.806 |  | 87.792 |  | 1.135 |  | 0.259 |  |
| Voice (1) * NARS * Interest |  | 0.222 |  | 0.751 |  | 88.001 |  | 0.295 |  | 0.769 |  |
| Voice (2) * NARS * Interest |  | -0.040 |  | 0.546 |  | 87.997 |  | -0.074 |  | 0.942 |  |
| Language (1) * Voice (1) * Exposure |  | -0.192 |  | 1.713 |  | 88.001 |  | -0.112 |  | 0.911 |  |
| Language (1) * Voice (2) * Exposure |  | 0.103 |  | 1.245 |  | 87.997 |  | 0.083 |  | 0.934 |  |
| Language (1) * NARS * Exposure |  | 0.207 |  | 0.492 |  | 87.792 |  | 0.420 |  | 0.675 |  |
| Voice (1) * NARS * Exposure |  | -0.010 |  | 0.459 |  | 88.001 |  | -0.022 |  | 0.982 |  |
| Voice (2) * NARS * Exposure |  | -0.039 |  | 0.333 |  | 87.997 |  | -0.116 |  | 0.908 |  |
| Language (1) * Interest * Exposure |  | 0.454 |  | 0.936 |  | 87.792 |  | 0.485 |  | 0.629 |  |
| Voice (1) * Interest * Exposure |  | 0.064 |  | 0.873 |  | 88.001 |  | 0.073 |  | 0.942 |  |
| Voice (2) * Interest * Exposure |  | -0.038 |  | 0.634 |  | 87.997 |  | -0.060 |  | 0.952 |  |
| NARS * Interest * Exposure |  | -0.212 |  | 0.252 |  | 87.792 |  | -0.842 |  | 0.402 |  |
| Language (1) * Voice (1) * NARS * Interest |  | -0.021 |  | 0.751 |  | 88.001 |  | -0.027 |  | 0.978 |  |
| Language (1) * Voice (2) * NARS * Interest |  | 0.027 |  | 0.546 |  | 87.997 |  | 0.050 |  | 0.961 |  |
| Language (1) * Voice (1) * NARS * Exposure |  | 0.019 |  | 0.459 |  | 88.001 |  | 0.041 |  | 0.967 |  |
| Language (1) * Voice (2) * NARS * Exposure |  | -0.059 |  | 0.333 |  | 87.997 |  | -0.177 |  | 0.860 |  |
| Language (1) * Voice (1) * Interest * Exposure |  | 0.054 |  | 0.873 |  | 88.001 |  | 0.062 |  | 0.951 |  |
| Language (1) * Voice (2) * Interest * Exposure |  | -0.013 |  | 0.634 |  | 87.997 |  | -0.021 |  | 0.983 |  |
| Language (1) * NARS * Interest * Exposure |  | -0.126 |  | 0.252 |  | 87.792 |  | -0.499 |  | 0.619 |  |
| Voice (1) * NARS * Interest * Exposure |  | 0.013 |  | 0.235 |  | 88.001 |  | 0.057 |  | 0.955 |  |
| Voice (2) * NARS * Interest * Exposure |  | 0.013 |  | 0.171 |  | 87.997 |  | 0.079 |  | 0.937 |  |
| Language (1) * Voice (1) * NARS * Interest * Exposure |  | 0.014 |  | 0.235 |  | 88.001 |  | 0.059 |  | 0.953 |  |
| Language (1) * Voice (2) * NARS * Interest * Exposure |  | 0.018 |  | 0.171 |  | 87.997 |  | 0.108 |  | 0.914 |  |
|  | | | | | | | | | | | |
| *Note.*  The intercept corresponds to the (unweighted) grand mean; for each factor with k levels, k - 1 parameters are estimated. Consequently, the estimates cannot be directly mapped to factor levels. Significant effects are highlighted in bold. | | | | | | | | | | | |

| Supplementary Table 37  *Results of linear mixed modeling with language, voice type, robots-related factors as predictors and credibility as outcome* | | | | | | | | | | | |
| --- | --- | --- | --- | --- | --- | --- | --- | --- | --- | --- | --- |
| Factor | | Estimate | | *SE* | | *df* | | *t* | | *p* | |
| Intercept |  | 7.896 |  | 5.808 |  | 87.735 |  | 1.359 |  | 0.177 |  |
| Language (1) |  | 1.226 |  | 5.807 |  | 87.701 |  | 0.211 |  | 0.833 |  |
| Voice (1) |  | 4.875 |  | 3.363 |  | 1227.836 |  | 1.450 |  | 0.147 |  |
| Voice (2) |  | -4.050 |  | 3.363 |  | 1227.836 |  | -1.204 |  | 0.229 |  |
| NARS |  | -1.020 |  | 1.546 |  | 87.701 |  | -0.659 |  | 0.511 |  |
| Interest |  | -2.012 |  | 2.951 |  | 87.701 |  | -0.682 |  | 0.497 |  |
| Exposure |  | -0.612 |  | 1.806 |  | 87.701 |  | -0.339 |  | 0.736 |  |
| Language (1) * Voice (1) |  | 3.018 |  | 3.363 |  | 1227.836 |  | 0.897 |  | 0.370 |  |
| Language (1) * Voice (2) |  | -2.259 |  | 3.363 |  | 1227.836 |  | -0.672 |  | 0.502 |  |
| Language (1) * NARS |  | -0.510 |  | 1.546 |  | 87.701 |  | -0.330 |  | 0.743 |  |
| Voice (1) * NARS |  | -0.702 |  | 0.896 |  | 1227.836 |  | -0.784 |  | 0.433 |  |
| Voice (2) * NARS |  | 0.802 |  | 0.896 |  | 1227.836 |  | 0.896 |  | 0.370 |  |
| Language (1) * Interest |  | -0.475 |  | 2.951 |  | 87.701 |  | -0.161 |  | 0.872 |  |
| Voice (1) * Interest |  | -2.139 |  | 1.709 |  | 1227.836 |  | -1.252 |  | 0.211 |  |
| Voice (2) * Interest |  | 1.784 |  | 1.709 |  | 1227.836 |  | 1.044 |  | 0.297 |  |
| NARS * Interest |  | 0.509 |  | 0.792 |  | 87.701 |  | 0.642 |  | 0.522 |  |
| Language (1) * Exposure |  | 0.348 |  | 1.806 |  | 87.701 |  | 0.192 |  | 0.848 |  |
| Voice (1) * Exposure |  | -1.067 |  | 1.046 |  | 1227.836 |  | -1.020 |  | 0.308 |  |
| Voice (2) * Exposure |  | 1.295 |  | 1.046 |  | 1227.836 |  | 1.238 |  | 0.216 |  |
| NARS * Exposure |  | 0.129 |  | 0.483 |  | 87.701 |  | 0.266 |  | 0.791 |  |
| Interest * Exposure |  | 0.368 |  | 0.920 |  | 87.701 |  | 0.400 |  | 0.690 |  |
| Language (1) * Voice (1) * NARS |  | -0.709 |  | 0.896 |  | 1227.836 |  | -0.791 |  | 0.429 |  |
| Language (1) * Voice (2) * NARS |  | 0.702 |  | 0.896 |  | 1227.836 |  | 0.783 |  | 0.434 |  |
| Language (1) * Voice (1) * Interest |  | -1.455 |  | 1.709 |  | 1227.836 |  | -0.851 |  | 0.395 |  |
| Language (1) * Voice (2) * Interest |  | 1.085 |  | 1.709 |  | 1227.836 |  | 0.635 |  | 0.526 |  |
| Language (1) * NARS * Interest |  | 0.182 |  | 0.792 |  | 87.701 |  | 0.230 |  | 0.819 |  |
| Voice (1) * NARS * Interest |  | 0.491 |  | 0.458 |  | 1227.836 |  | 1.072 |  | 0.284 |  |
| Voice (2) * NARS * Interest |  | -0.454 |  | 0.458 |  | 1227.836 |  | -0.991 |  | 0.322 |  |
| Language (1) * Voice (1) * Exposure |  | -0.769 |  | 1.046 |  | 1227.836 |  | -0.736 |  | 0.462 |  |
| Language (1) * Voice (2) * Exposure |  | 1.133 |  | 1.046 |  | 1227.836 |  | 1.083 |  | 0.279 |  |
| Language (1) * NARS * Exposure |  | -0.067 |  | 0.483 |  | 87.701 |  | -0.138 |  | 0.890 |  |
| Voice (1) * NARS * Exposure |  | 0.222 |  | 0.280 |  | 1227.836 |  | 0.793 |  | 0.428 |  |
| Voice (2) * NARS * Exposure |  | -0.314 |  | 0.280 |  | 1227.836 |  | -1.121 |  | 0.262 |  |
| Language (1) * Interest * Exposure |  | -0.220 |  | 0.920 |  | 87.701 |  | -0.239 |  | 0.812 |  |
| Voice (1) * Interest * Exposure |  | 0.595 |  | 0.533 |  | 1227.836 |  | 1.117 |  | 0.264 |  |
| Voice (2) * Interest * Exposure |  | -0.680 |  | 0.533 |  | 1227.836 |  | -1.277 |  | 0.202 |  |
| NARS * Interest * Exposure |  | -0.086 |  | 0.248 |  | 87.701 |  | -0.346 |  | 0.730 |  |
| Language (1) * Voice (1) * NARS * Interest |  | 0.368 |  | 0.458 |  | 1227.836 |  | 0.802 |  | 0.423 |  |
| Language (1) * Voice (2) * NARS * Interest |  | -0.368 |  | 0.458 |  | 1227.836 |  | -0.804 |  | 0.422 |  |
| Language (1) * Voice (1) * NARS * Exposure |  | 0.201 |  | 0.280 |  | 1227.836 |  | 0.720 |  | 0.472 |  |
| Language (1) * Voice (2) * NARS * Exposure |  | -0.333 |  | 0.280 |  | 1227.836 |  | -1.189 |  | 0.235 |  |
| Language (1) * Voice (1) * Interest * Exposure |  | 0.344 |  | 0.533 |  | 1227.836 |  | 0.645 |  | 0.519 |  |
| Language (1) * Voice (2) * Interest * Exposure |  | -0.536 |  | 0.533 |  | 1227.836 |  | -1.006 |  | 0.315 |  |
| Language (1) * NARS * Interest * Exposure |  | 0.048 |  | 0.248 |  | 87.701 |  | 0.193 |  | 0.847 |  |
| Voice (1) * NARS * Interest * Exposure |  | -0.109 |  | 0.144 |  | 1227.836 |  | -0.757 |  | 0.449 |  |
| Voice (2) * NARS * Interest * Exposure |  | 0.160 |  | 0.144 |  | 1227.836 |  | 1.115 |  | 0.265 |  |
| Language (1) * Voice (1) * NARS * Interest * Exposure |  | -0.087 |  | 0.144 |  | 1227.836 |  | -0.604 |  | 0.546 |  |
| Language (1) * Voice (2) * NARS * Interest * Exposure |  | 0.158 |  | 0.144 |  | 1227.836 |  | 1.100 |  | 0.272 |  |
|  | | | | | | | | | | | |
| *Note.*  The intercept corresponds to the (unweighted) grand mean; for each factor with k levels, k - 1 parameters are estimated. Consequently, the estimates cannot be directly mapped to factor levels. Significant effects are highlighted in bold. | | | | | | | | | | | |

| Supplementary Table 38  *Results of linear mixed modeling with language, voice type, robots-related factors as predictors and human-likeness (personality) as outcome* | | | | | | | | | | | |
| --- | --- | --- | --- | --- | --- | --- | --- | --- | --- | --- | --- |
| Factor | | Estimate | | *SE* | | *df* | | *t* | | *p* | |
| Intercept |  | 9.300 |  | 4.732 |  | 87.844 |  | 1.965 |  | 0.053 |  |
| Language (1) |  | 3.224 |  | 4.731 |  | 87.790 |  | 0.681 |  | 0.497 |  |
| Voice (1) |  | -1.194 |  | 5.951 |  | 88.005 |  | -0.201 |  | 0.841 |  |
| Voice (2) |  | 0.096 |  | 4.396 |  | 88.001 |  | 0.022 |  | 0.983 |  |
| NARS |  | -1.306 |  | 1.260 |  | 87.790 |  | -1.036 |  | 0.303 |  |
| Interest |  | -2.918 |  | 2.404 |  | 87.790 |  | -1.214 |  | 0.228 |  |
| Exposure |  | -1.521 |  | 1.472 |  | 87.790 |  | -1.033 |  | 0.304 |  |
| Language (1) * Voice (1) |  | -3.328 |  | 5.951 |  | 88.005 |  | -0.559 |  | 0.577 |  |
| Language (1) * Voice (2) |  | 0.569 |  | 4.396 |  | 88.001 |  | 0.130 |  | 0.897 |  |
| Language (1) * NARS |  | -0.970 |  | 1.260 |  | 87.790 |  | -0.770 |  | 0.443 |  |
| Voice (1) * NARS |  | 1.036 |  | 1.585 |  | 88.005 |  | 0.654 |  | 0.515 |  |
| Voice (2) * NARS |  | -0.235 |  | 1.171 |  | 88.001 |  | -0.201 |  | 0.841 |  |
| Language (1) * Interest |  | -2.114 |  | 2.404 |  | 87.790 |  | -0.879 |  | 0.382 |  |
| Voice (1) * Interest |  | 2.301 |  | 3.024 |  | 88.005 |  | 0.761 |  | 0.449 |  |
| Voice (2) * Interest |  | -0.778 |  | 2.234 |  | 88.001 |  | -0.348 |  | 0.729 |  |
| NARS * Interest |  | 0.704 |  | 0.645 |  | 87.790 |  | 1.092 |  | 0.278 |  |
| Language (1) * Exposure |  | -0.643 |  | 1.472 |  | 87.790 |  | -0.437 |  | 0.663 |  |
| Voice (1) * Exposure |  | 1.776 |  | 1.851 |  | 88.005 |  | 0.960 |  | 0.340 |  |
| Voice (2) * Exposure |  | -0.627 |  | 1.367 |  | 88.001 |  | -0.459 |  | 0.648 |  |
| NARS * Exposure |  | 0.358 |  | 0.394 |  | 87.790 |  | 0.908 |  | 0.366 |  |
| Interest * Exposure |  | 0.767 |  | 0.750 |  | 87.790 |  | 1.023 |  | 0.309 |  |
| Language (1) * Voice (1) * NARS |  | 0.927 |  | 1.585 |  | 88.005 |  | 0.585 |  | 0.560 |  |
| Language (1) * Voice (2) * NARS |  | -0.032 |  | 1.171 |  | 88.001 |  | -0.027 |  | 0.978 |  |
| Language (1) * Voice (1) * Interest |  | 2.399 |  | 3.024 |  | 88.005 |  | 0.793 |  | 0.430 |  |
| Language (1) * Voice (2) * Interest |  | -0.485 |  | 2.234 |  | 88.001 |  | -0.217 |  | 0.829 |  |
| Language (1) * NARS * Interest |  | 0.605 |  | 0.645 |  | 87.790 |  | 0.937 |  | 0.351 |  |
| Voice (1) * NARS * Interest |  | -0.637 |  | 0.811 |  | 88.005 |  | -0.785 |  | 0.435 |  |
| Voice (2) * NARS * Interest |  | 0.137 |  | 0.599 |  | 88.001 |  | 0.229 |  | 0.820 |  |
| Language (1) * Voice (1) * Exposure |  | 1.434 |  | 1.851 |  | 88.005 |  | 0.774 |  | 0.441 |  |
| Language (1) * Voice (2) * Exposure |  | -0.109 |  | 1.367 |  | 88.001 |  | -0.079 |  | 0.937 |  |
| Language (1) * NARS * Exposure |  | 0.190 |  | 0.394 |  | 87.790 |  | 0.482 |  | 0.631 |  |
| Voice (1) * NARS * Exposure |  | -0.525 |  | 0.495 |  | 88.005 |  | -1.059 |  | 0.292 |  |
| Voice (2) * NARS * Exposure |  | 0.156 |  | 0.366 |  | 88.001 |  | 0.427 |  | 0.670 |  |
| Language (1) * Interest * Exposure |  | 0.407 |  | 0.750 |  | 87.790 |  | 0.543 |  | 0.589 |  |
| Voice (1) * Interest * Exposure |  | -1.043 |  | 0.943 |  | 88.005 |  | -1.107 |  | 0.271 |  |
| Voice (2) * Interest * Exposure |  | 0.367 |  | 0.696 |  | 88.001 |  | 0.527 |  | 0.600 |  |
| NARS * Interest * Exposure |  | -0.181 |  | 0.202 |  | 87.790 |  | -0.898 |  | 0.372 |  |
| Language (1) * Voice (1) * NARS * Interest |  | -0.602 |  | 0.811 |  | 88.005 |  | -0.743 |  | 0.460 |  |
| Language (1) * Voice (2) * NARS * Interest |  | 0.018 |  | 0.599 |  | 88.001 |  | 0.031 |  | 0.976 |  |
| Language (1) * Voice (1) * NARS * Exposure |  | -0.373 |  | 0.495 |  | 88.005 |  | -0.753 |  | 0.453 |  |
| Language (1) * Voice (2) * NARS * Exposure |  | -0.003 |  | 0.366 |  | 88.001 |  | -0.008 |  | 0.994 |  |
| Language (1) * Voice (1) * Interest * Exposure |  | -0.888 |  | 0.943 |  | 88.005 |  | -0.941 |  | 0.349 |  |
| Language (1) * Voice (2) * Interest * Exposure |  | 0.168 |  | 0.696 |  | 88.001 |  | 0.241 |  | 0.810 |  |
| Language (1) * NARS * Interest * Exposure |  | -0.123 |  | 0.202 |  | 87.790 |  | -0.610 |  | 0.543 |  |
| Voice (1) * NARS * Interest * Exposure |  | 0.316 |  | 0.254 |  | 88.005 |  | 1.245 |  | 0.217 |  |
| Voice (2) * NARS * Interest * Exposure |  | -0.089 |  | 0.188 |  | 88.001 |  | -0.472 |  | 0.638 |  |
| Language (1) * Voice (1) * NARS * Interest * Exposure |  | 0.219 |  | 0.254 |  | 88.005 |  | 0.864 |  | 0.390 |  |
| Language (1) * Voice (2) * NARS * Interest * Exposure |  | -0.024 |  | 0.188 |  | 88.001 |  | -0.127 |  | 0.899 |  |
|  | | | | | | | | | | | |
| *Note.*  The intercept corresponds to the (unweighted) grand mean; for each factor with k levels, k - 1 parameters are estimated. Consequently, the estimates cannot be directly mapped to factor levels. Significant effects are highlighted in bold. | | | | | | | | | | | |

| Supplementary Table 39  *Results of linear mixed modeling with language, voice type, robots-related factors as predictors and eeriness as outcome* | | | | | | | | | | | |
| --- | --- | --- | --- | --- | --- | --- | --- | --- | --- | --- | --- |
| Factor | | Estimate | | *SE* | | *df* | | *t* | | *p* | |
| Intercept |  | -5.214 |  | 7.814 |  | 87.962 |  | -0.667 |  | 0.506 |  |
| Language (1) |  | -7.793 |  | 7.813 |  | 87.955 |  | -0.997 |  | 0.321 |  |
| Voice (1) |  | 5.579 |  | 3.898 |  | 1227.785 |  | 1.431 |  | 0.153 |  |
| Voice (2) |  | 1.505 |  | 3.898 |  | 1227.785 |  | 0.386 |  | 0.699 |  |
| NARS |  | 2.295 |  | 2.081 |  | 87.955 |  | 1.103 |  | 0.273 |  |
| Interest |  | 3.949 |  | 3.971 |  | 87.955 |  | 0.995 |  | 0.323 |  |
| Exposure |  | 1.880 |  | 2.430 |  | 87.955 |  | 0.774 |  | 0.441 |  |
| Language (1) * Voice (1) |  | 7.565 |  | 3.898 |  | 1227.785 |  | 1.941 |  | 0.053 |  |
| Language (1) * Voice (2) |  | 0.823 |  | 3.898 |  | 1227.785 |  | 0.211 |  | 0.833 |  |
| Language (1) * NARS |  | 2.186 |  | 2.081 |  | 87.955 |  | 1.050 |  | 0.296 |  |
| **Voice (1) * NARS** |  | **-2.296** |  | **1.038** |  | **1227.785** |  | **-2.212** |  | **0.027** |  |
| Voice (2) * NARS |  | 0.037 |  | 1.038 |  | 1227.785 |  | 0.036 |  | 0.972 |  |
| Language (1) * Interest |  | 4.599 |  | 3.971 |  | 87.955 |  | 1.158 |  | 0.250 |  |
| Voice (1) * Interest |  | -3.194 |  | 1.981 |  | 1227.785 |  | -1.612 |  | 0.107 |  |
| Voice (2) * Interest |  | -1.084 |  | 1.981 |  | 1227.785 |  | -0.547 |  | 0.584 |  |
| NARS * Interest |  | -1.129 |  | 1.065 |  | 87.955 |  | -1.060 |  | 0.292 |  |
| Language (1) * Exposure |  | 1.490 |  | 2.430 |  | 87.955 |  | 0.613 |  | 0.541 |  |
| Voice (1) * Exposure |  | -1.731 |  | 1.213 |  | 1227.785 |  | -1.428 |  | 0.154 |  |
| Voice (2) * Exposure |  | -0.556 |  | 1.213 |  | 1227.785 |  | -0.459 |  | 0.646 |  |
| NARS * Exposure |  | -0.470 |  | 0.650 |  | 87.955 |  | -0.723 |  | 0.471 |  |
| Interest * Exposure |  | -0.930 |  | 1.238 |  | 87.955 |  | -0.751 |  | 0.454 |  |
| Language (1) * Voice (1) * NARS |  | -2.205 |  | 1.038 |  | 1227.785 |  | -2.124 |  | 0.034 |  |
| Language (1) * Voice (2) * NARS |  | -0.087 |  | 1.038 |  | 1227.785 |  | -0.084 |  | 0.933 |  |
| Language (1) * Voice (1) * Interest |  | -3.915 |  | 1.981 |  | 1227.785 |  | -1.976 |  | 0.048 |  |
| Language (1) * Voice (2) * Interest |  | -0.589 |  | 1.981 |  | 1227.785 |  | -0.297 |  | 0.766 |  |
| Language (1) * NARS * Interest |  | -1.290 |  | 1.065 |  | 87.955 |  | -1.211 |  | 0.229 |  |
| **Voice (1) * NARS * Interest** |  | **1.151** |  | **0.531** |  | **1227.785** |  | **2.165** |  | **0.031** |  |
| Voice (2) * NARS * Interest |  | 0.138 |  | 0.531 |  | 1227.785 |  | 0.261 |  | 0.794 |  |
| Language (1) * Voice (1) * Exposure |  | -1.639 |  | 1.213 |  | 1227.785 |  | -1.351 |  | 0.177 |  |
| Language (1) * Voice (2) * Exposure |  | -0.822 |  | 1.213 |  | 1227.785 |  | -0.678 |  | 0.498 |  |
| Language (1) * NARS * Exposure |  | -0.422 |  | 0.650 |  | 87.955 |  | -0.649 |  | 0.518 |  |
| **Voice (1) * NARS * Exposure** |  | **0.645** |  | **0.325** |  | **1227.785** |  | **1.989** |  | **0.047** |  |
| Voice (2) * NARS * Exposure |  | 0.039 |  | 0.325 |  | 1227.785 |  | 0.121 |  | 0.904 |  |
| Language (1) * Interest * Exposure |  | -0.887 |  | 1.238 |  | 87.955 |  | -0.717 |  | 0.475 |  |
| Voice (1) * Interest * Exposure |  | 1.031 |  | 0.617 |  | 1227.785 |  | 1.670 |  | 0.095 |  |
| Voice (2) * Interest * Exposure |  | 0.353 |  | 0.617 |  | 1227.785 |  | 0.572 |  | 0.568 |  |
| NARS * Interest * Exposure |  | 0.245 |  | 0.334 |  | 87.955 |  | 0.736 |  | 0.464 |  |
| **Language (1) * Voice (1) * NARS * Interest** |  | **1.124** |  | **0.531** |  | **1227.785** |  | **2.115** |  | **0.035** |  |
| Language (1) * Voice (2) * NARS * Interest |  | 0.121 |  | 0.531 |  | 1227.785 |  | 0.229 |  | 0.819 |  |
| Language (1) * Voice (1) * NARS * Exposure |  | 0.476 |  | 0.325 |  | 1227.785 |  | 1.468 |  | 0.142 |  |
| Language (1) * Voice (2) * NARS * Exposure |  | 0.174 |  | 0.325 |  | 1227.785 |  | 0.537 |  | 0.591 |  |
| Language (1) * Voice (1) * Interest * Exposure |  | 0.923 |  | 0.617 |  | 1227.785 |  | 1.494 |  | 0.135 |  |
| Language (1) * Voice (2) * Interest * Exposure |  | 0.397 |  | 0.617 |  | 1227.785 |  | 0.642 |  | 0.521 |  |
| Language (1) * NARS * Interest * Exposure |  | 0.259 |  | 0.334 |  | 87.955 |  | 0.776 |  | 0.440 |  |
| **Voice (1) * NARS * Interest * Exposure** |  | **-0.405** |  | **0.166** |  | **1227.785** |  | **-2.431** |  | **0.015** |  |
| Voice (2) * NARS * Interest * Exposure |  | -0.021 |  | 0.166 |  | 1227.785 |  | -0.124 |  | 0.901 |  |
| Language (1) * Voice (1) * NARS * Interest * Exposure |  | -0.265 |  | 0.166 |  | 1227.785 |  | -1.593 |  | 0.111 |  |
| Language (1) * Voice (2) * NARS * Interest * Exposure |  | -0.081 |  | 0.166 |  | 1227.785 |  | -0.490 |  | 0.625 |  |
|  | | | | | | | | | | | |
| *Note.*  The intercept corresponds to the (unweighted) grand mean; for each factor with k levels, k - 1 parameters are estimated. Consequently, the estimates cannot be directly mapped to factor levels. Significant effects are highlighted in bold. | | | | | | | | | | | |
