# Supplementary material for: The Human Takes It All: Humanlike Synthesized Voices Are Perceived as Less Eerie and More Likable. Evidence From a Subjective Ratings Study
Source: Front Neurorobot. 2020 Dec 16;14:593732. doi: 10.3389/fnbot.2020.593732 (PMC7772241; doi:10.3389/fnbot.2020.593732)
Supplement: Supplementary file 2 [file Table_2.DOCX]

Supplementary Figure Caption

*Supplementary Figure 1.* Voice and speaker ratings in accordance with gender and native language: (A) *Intelligibility* (B) *Prosody* (C) *Trustworthiness* (D) *Confidence* (E) *Enthusiasm* (F) *Pleasantness* (G) *Naturalness* (H) *Human-likeness* (voice) (I) *Likability* (J) *Appeal* (K) *Credibility* (L) *Human-likeness (personality)* (M) *Eeriness*.
